# Vaginal-spray *Bacillus* spore probiotics as a potential treatment and reducing recurrence of bacterial vaginosis: randomized, double-blind, and controlled pilot study

**DOI:** 10.1038/s43856-025-01236-4

**Published:** 2025-11-18

**Authors:** Huyen Thi Bui, Anh Thi Phuong Bui, Hong Thi Ngo, Xuan Thi Ngo, Huyen Thi Nguyen, Anh Hoa Nguyen, Van Cam Tran, Tung Dinh Pham, Anh Thi Van Nguyen

**Affiliations:** 1 Spobiotic Research Center, ANABIO R&D Ltd. Company, Ha Dong, Hanoi Vietnam; 2 Bac Ninh Center for Disease Control and Prevention, Nguyen Quyen, Vo Cuong, Bac Ninh, Vietnam; 3 LiveSpo Pharma Ltd. Company, N03T5, Ngoai Giao Doan Urban, Xuan Dinh, Hanoi Vietnam; 4 National Hospital of Dermatology and Venereology, Kim Liên, Hanoi Vietnam; 5 The National Center for Health Information, Ministry of Health, Giang Vo, Hanoi Vietnam; 6 Faculty of Mathematics - Mechanics - Informatics, VNU University of Science, Vietnam National University_Hanoi, Thanh Xuan, Hanoi Vietnam

**Keywords:** Randomized controlled trials, Clinical microbiology, Microbiome, Bacterial infection

## Abstract

**Background:**

Bacterial vaginosis (BV) is a common vaginal disorder characterized by reduced beneficial species and overgrowth of pathogens. Probiotics, especially direct vaginal sprays, offer a promising alternative to antibiotics by restoring healthy vaginal microbiota, relieving symptoms, and preventing recurrence.

**Methods:**

We conducted a clinical trial (ClinicalTrials.gov: NCT06165354; 8/12/2023) to evaluate the effectiveness of the vaginal-spray probiotic LiveSpo X-Secret, containing *Bacillus subtilis*, *B. clausii*, and *B. coagulans* spores (≥1 billion CFU/mL). A total of 120 women were randomized equally into Control and X-Secret groups at Bac Ninh CDC. Outcomes were assessed at day 7 (end of standard treatment) for symptom resolution and day 28 (21 days post-treatment) for recurrence. Both Per-Protocol and Intention-to-Treat analyses were performed to assess efficacy and account for attrition.

**Results:**

Here we show that no adverse events occur in either group. Compared to the Control, the X-Secret group exhibits a faster reduction in BV symptoms (odor, discharge, pH >4.5, and itching) by days 7 and 28, with odds ratios at day 28 of 3.61, 4.87, 2.95, and 3.34, respectively. Vaginal swab analysis reveals a 3.7–225.3-fold greater reduction in *Gardnerella vaginalis*. By day 28, metagenomic profiling indicates increases in beneficial species *Lactobacillus crispatus* (3.71-fold) and *Streptococcus salivarius* (from non-detectable to 2.1%), along with reductions in harmful species *G. vaginalis* (14.29-fold) and *Sneathia sanguinegens* (806-fold).

**Conclusions:**

The vaginal-spray *Bacillus* spore probiotic (LiveSpo X-Secret) provides a safe, convenient, and effective approach for BV treatment and reducing recurrence, supporting its potential as an adjunctive therapy for maintaining vaginal health.

**Clinical trial number:**

ClinicalTrials.gov NCT06165354, first posted 8/12/2023.

## Plain language summary

Bacterial vaginosis (BV) is a common vaginal infection affecting women worldwide. Current antibiotic treatments often fail to prevent recurrence. Probiotics offer a promising alternative by restoring the natural balance of vaginal bacteria, but oral probiotics are slow, and suppositories can be inconvenient. We tested a fast, easy-to-use probiotic spray applied around the vagina (LiveSpo X-Secret), containing billions of *Bacillus* spores—beneficial bacteria that survive harsh conditions. Our clinical study shows that the LiveSpo X-Secret quickly and effectively reduces BV symptoms (odor, abnormal discharge, high pH, and itching) by lowering overgrowth of some bacteria, while supporting beneficial bacteria growth to minimize symptom recurrence. This suggests that LiveSpo X-Secret is a safe, fast, and effective supportive treatment for BV and in reducing recurrence.

## Abstract (web summary)

Bui et al. conduct a clinical trial evaluating a vaginal-spray *Bacillus* spore probiotic (LiveSpo X-Secret) as adjunctive therapy for bacterial vaginosis. When combined with standard treatment, it rapidly relieves symptoms within 7 days and alone prevent recurrence over 28 days by reducing *Gardnerella vaginalis* and improving vaginal microbiota.

## Introduction

Bacterial vaginosis (BV) is a prevalent vaginal infection affecting women worldwide, particularly in tropical and underdeveloped regions, where over 20% of women of reproductive age are impacted^1^. This condition poses a significant public health concern, with an annual global economic burden estimated in the billions USD, due to its general asymptomatic nature, which can lead to it being overlooked. Moreover, if left untreated, BV can lead to adverse outcomes such as preterm birth, pelvic inflammatory disease, and increased susceptibility to sexually transmitted infections (STIs)^1,2^. BV is normally induced by alterations in the vaginal microbiota, particularly a reduction in beneficial genera and species such as *Lactobacillus* sp. and *Bifidobacterium* sp., and an increase of pathogenic genus/species like *Gardnerella vaginalis, Prevotella* sp., *Atopobium sp., Mobiluncus sp., Mycoplasma hominis*, and *Sneathia* sp^3–7^. Imbalance in the vaginal microbiota is sometime associated with co-infection with other pathogenic microorganisms such as *Candida albicans*, *Trichomonas vaginalis*^8,9^. Despite its high incidence and potential complications, the precise etiology of BV remains multifactorial, with contributing factors including sexual activity, douching, and hormonal fluctuations^10–12^. Therefore, addressing the complex interplay between host and microbial factors necessitates innovative therapeutic approaches. Antibiotics containing metronidazole and clindamycin are often recommended for the treatment of pathogenic bacteria causing BV. However, the treatment fails to prevent recurrence of bacterial vaginosis (BV) or abnormal vaginal flora in most women. Factors linked to recurrence suggest that sexual transmission may play a role in the development of recurrent BV. It is also important to note that the use of broad-spectrum antibiotics can lead to side effects and contribute to the emergence of antibiotic resistance, which can increase the risk of recurrent infections^13–15^.

One promising approach involves the use of probiotics, which aim to restore the vaginal microbiota’s balance and bolster its protective mechanisms. By introducing beneficial bacteria that maintain a healthy balance in the body, especially in the intestinal and vaginal tracts, probiotic treatments hold the potential to mitigate BV symptoms, prevent recurrence, and improve overall vaginal health^16^. Previous studies have found that oral-administrative probiotics containing strains of *Lactobacillus* sp. can help reduce the incidence and severity of vaginal infections, including bacterial vaginosis and yeast infections. The mechanism involves the ability of *Lactobacillus* sp. to transit through the intestinal tract to the anus and subsequently move to the vaginal tract^17^. The bacterium naturally secretes lactic acid, which helps to maintain an acidic pH environment in the vagina. The acidic environment creates an inhospitable condition for the growth of pathogenic microorganisms, such as *Candida albicans* and *T. vaginalis*, which thrive in alkaline environments. Additionally, *Lactobacillus* sp. produce hydrogen peroxide and other antimicrobial substances, further inhibiting the growth of harmful bacteria and fungi^17–20^. On the other hand, other studies have shown that orally administered probiotics do not effectively improve BV and suggest that alternative routes, directly in the vagina, may yield better results^21,22^. In fact, vaginal suppository *Lactobacillus* probiotics have emerged as a promising complementary therapy for treating vaginal infections^23^. However, formulating liquid *Lactobacillus* probiotics remains a technical challenge, as these bacteria are generally unstable in aqueous suspensions at room temperature, limiting their viability during storage. While oil-based suspensions can enhance *Lactobacillus* stability, they are not suitable for direct vaginal administration. Moreover, suppositories, while potentially more effective than oral administration-which has delayed efficacy due to gastrointestinal transit-may be inconvenient for daily use. Therefore, a vaginal spray probiotic offers an alternative approach, allowing direct and convenient application without interfering with daily activities.

*Bacillus* spore probiotics, such as *B. coagulans*, present a compelling alternative for vaginal health maintenance with similar objectives as *Lactobacillus-*based probiotics. The clinical trial by Ratna et al. (2012) showed that 80% of women in the group taking 10^9^ CFU of *B. coagulans* Unique IS-2 (ATCC PTA-11748) per oral-capsule daily significantly reduced bacterial vaginosis symptoms compared to the control group (only 45% of subjects showed reduction)^24^. Tsimaris et al. (2019) also demonstrated that *B. coagulans* suppository-probiotics helped alleviate typical symptoms of vaginitis such as itching, vaginal burning, vaginal irritation, and discharge^25^. One notable advantage of *Bacillus* species is their ability to form spores, which are heat-stable and acid-resistant^26,27^. This characteristic allows for long-time storage and convenient administration in vaginal-spray liquid-form suspension. The heat and acid stability of *Bacillus* spores ensure their survival through various environmental conditions during manufacturing, storage, and in the vaginal tract. Here, we explored the safety and efficacy of direct vaginal-spray *Bacillus* spore probiotics as a fast and effective symptomatic treatment for BV. The probiotic product (LiveSpo X-Secret) tested in this study is a physiological saline NaCl 0.9% suspension at high concentration ≥1 billion CFU/mL of three strains: *B. subtilis* ANA46, *B. clausii* ANA39, and *B. coagulans* ANA40. This product is conveniently administered two times a day to patients through direct vaginal spraying, in addition to the standard 7-day antibiotic treatment.

In this study, we show that LiveSpo X-Secret is safe and effective in reducing clinical symptoms of BV, including bad odor, abnormal vaginal discharge, vaginal pH > 4.5, itching, burning micturition, and abdominal pain, as well as subclinical indices, such as changes in *G. vaginalis* concentration and vaginal microbiota by day 7 (at the end of standard treatment) and day 28 (21 days post treatment). These preliminary results demonstrate that vaginal-spray *Bacillus* spores provide a safe, convenient, and effective adjunctive approach for reducing BV recurrence.

## Methods

### Materials

Vaginal-spray probiotics LiveSpo X-Secret (LiveSpo Pharma, Hanoi, Vietnam) was formulated as a 0.9% NaCl physiological saline suspension containing *Bacillus subtilis* ANA46, *B. clausii* ANA39 and *B. coagulans* ANA40 spores at ≥ 5 × 10^9^ CFU/ 5 mL. The suspension was filled into a plastic sprayer featuring a 360-degree rotating nozzle, allowing for approximately 3 cm intravaginal insertion before spraying, as well as application to the surrounding external areas, in accordance with the manufacturer’s instructions. The product was manufactured under ISO 13485:2016 standard and patent No. 40128 granted by Vietnam Intellectual Property Office. Prior to manufacturing and conducting clinical studies, the strains underwent rigorous *in-vitro* physiological, biochemical, and genetic testing to guarantee their safety and probiotic properties. The relevant information can be accessed from Supplementary Tables 1-2, Supplementary Figs. 1–6 and Supplementary Tables 3–5. The results also indicated that these strains have high efficiency in spore formation (>90%) and are heat-stable (>65 °C) (Supplementary Table 1), facilitating cost-effective production of liquid-form spores and ensuring stable viable *Bacillus* counts during room temperature storage for 24 months (Supplementary Fig. 7). Sub-acute toxicity and vaginal mucosal irritation studies in rabbits, using doses 5-fold and 1.7-fold higher than the typical human dose, respectively, confirmed that LiveSpo X-Secret is non-toxic (Supplementary Data 1: Test Results – Sub-acute Toxicity and Supplementary Data 2: Vaginal Mucosal Irritation). The taste and smell of LiveSpo X-Secret are indistinguishable from 0.9% NaCl physiological saline (B.Braun, Germany). Contained in an opaque plastic container, the color and turbidity of the LiveSpo X-Secret suspension are indiscernible.

### Study design and patient collection

This was a double-blind, randomized, controlled intervention pilot study, with the control group (named “Control” group) received 0.9% NaCl and an experimental group (named “X-Secret” group) received the probiotics LiveSpo X-Secret. The study was implemented at Bac Ninh Center for Disease Control (CDC) from 15 December 2023 to 15 June 2024.

Women between the ages of 18 and 60 who met the inclusion criteria, including a diagnosis of bacterial vaginosis according to Amsel’s criteria: (i) a thin, homogeneous vaginal discharge; (ii) vaginal pH exceeding 4.5; (iii) a ‘fishy’ odor of vaginal fluid upon the addition of 10% KOH (whiff test); and (iv) the presence of clue cells in saline wet mount preparations observed microscopically. Eligible patients could exhibit additional symptoms such as vaginal odor, itching, burning during urination, or lower abdominal pain, and could also test positive for *Gardnerella vaginalis* (GV) either as a single infection or co-infected with either *Mycoplasma hominis* (MH) or *M. genitalium* (MG). Exclusion criteria included (i) pregnant women, diabetics, taking antibiotics, antimicrobials, or probiotics in the preceding 14 days, (ii) positive tests for sexually transmitted infections (STIs) including HSV-1/2, *Candida albicans* (CA), *Trichomonas vaginalis* (TV)*, Neisseria gonorrhoeae* (NG)*, and Chlamydia trachomatis* (CT) (iii) unexplained vaginal bleeding or cancer, (iv) a history of drug allergies or hypersensitivity to any ingredient in probiotics or placebo, (v) meeting the criteria for mental, cognitive, depressive, or anxiety disorders, (vi) simultaneous participation in another clinical trial, (vii) unwillingness to provide informed consent, or (viii) withdrawal from the study before day3 at Bac Ninh CDC.

Sample size of participants was calculated based on a hypothesis is that LiveSpo X-Secret alleviates vaginal-infection symptoms about 25% more effectively to be 49 per group at day 7 (end of antibiotic treatment) (alpha = 0.05; power level = 0.8). This was deduced based on the anticipation that 85% of patients in the X-Secret group would be symptom-free from two among the three typical symptoms of BV, including vaginal odor, abnormal vaginal discharge, and vaginal pH >4.5, at day 7 of the follow-up, in contrast to 60% of patients in the Control group. In fact, a total of 223 participants were screened for eligibility and 120 eligible participants (*n* = 60 per group) were randomly assigned by lottery to Control and X-Secret group to reduce the risk of about 20% patient’s drop out during follow-up treatment.

**Fig. 1:**
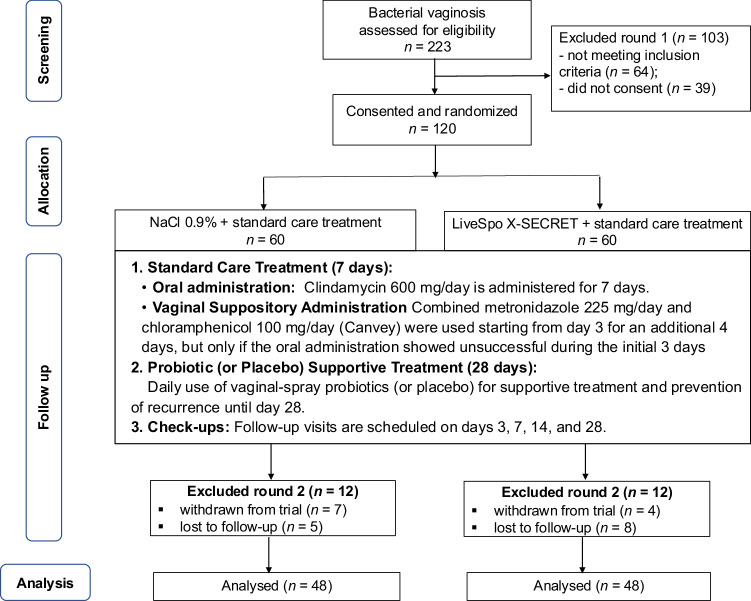
Diagram displaying the flow of participants involved in the clinical study. From the clinical database, participants were screened for eligibility. Eligible participants who provided consent were randomized in either Control or X-Secret group. Measurements took place at days 0, 3, 7, 14, and 28 of treatment period. Participants were recruited from December 2023 to May 2024. Clinical and subclinical data collection and analysis were conducted from December 2023 to June 2024.

Random allocation by the principal investigator was conducted using a sealed-box draw of pre-prepared opaque envelopes with equally distributed coded sheets (1:1 ratio), supervised by a study nurse and an independent monitor, with immediate documentation in participant records, ensuring transparency and minimizing bias. The participants were randomly assigned to two groups: the Control or X-Secret group. The Control and X-Secret groups were assigned the numbers 1 or 2, respectively, and this information was also confidential to all patients, nurses and investigators, except for the principal investigator and the data analyst. The study flow was presented in Fig. 1.

### Questionnaires, treatment procedures, and clinical observation

Patients provided personal information related to their living habits. Regarding the standard treatment of carriers, clindamycin 600 mg/day was initially prescribed for 7 days via oral administration only. If symptoms did not improve within the first 3 days, patients were prescribed a combination of metronidazole 225 mg/day and chloramphenicol 100 mg/day (Canvey) from day 3 for an additional 4 days via suppository administration (Fig. 1). In the trial, in addition to the standard care, the patients were provided with coded sprayers in the form of blind samples and were trained to administer them with dosages of 3 sprays (equivalent to 300 µl) per application of either placebo 0.9% NaCl physiological saline or LiveSpo X-Secret (equivalent to 3 × 10^8^ *Bacillus* spores) into the vaginal area, 2 times/day (before a short snap or rest at noon between 12 pm −2 pm, and before night sleep, between 9 pm and 11 pm). Participants were instructed to self-administer vaginal spray every day for 28 consecutive days with either LiveSpo or placebo. Participants were scheduled for additional four visits at day 3 ± 1 (2nd visit), day 7 ± 2 (3rd visit), day 14 ± 2 (4th visit), and day 28 ± 2 (5th visit) to check symptoms and assess adverse events (if any). We evaluated the primary outcome-symptom reduction at two key time points (day 7 and day 28), corresponding to the end of antibiotic treatment and the end of post-treatment follow-up. The study’s primary outcomes included three typical BV symptoms-vaginal odor, abnormal vaginal discharge, and vaginal pH >4.5-along with three additional BV-associated symptoms-itching, burning during urination, and abdominal pain-assessed at days 3, 7, 14, and 28, compared to baseline (day 0).

### Real-time PCR detection assay

The vaginal discharge samples were collected at 5 time points (days 0, 3, 7, 14, and 28) using polyurethane foam swabs (Henso Medical, Hangzhou, China) brushed against the lateral wall of the vagin. All vaginal specimens were pre-treated with 1 mL of 0.9% physiological saline solution and vortexed vigorously for 30 s. Total DNA was extracted from 200 µL vaginal specimens (repeated twice) using the QIAamp DNA Mini Kit (Qiagen, Hilden, Germany) according to the manufacturer’s instructions. 100 µl of the purified DNA/RNA was aliquoted into three PCR tubes (approximately 30 µl/tube) for storage at −80 °C. The Real-time PCR screening assay covers nine microorganism, including three species associated with BV: GV, MH, MG, and six species associated with other STIs: HSV-1, HSV-2, CA, TV, NG, and CT, under following conditions: 95 °C for 10 min, amplification for 45 cycles at 95 °C for 15 s, 60 °C for 45 s (Bui et al., 2023)^28^. The read-out standardization for pathogen analysis was set at C_t_ < 40 to confirm true positives. Simultaneously, another real-time PCR SYBR Green assay was carried out on vaginal swab samples on the same time points to confirm the presence of *B. subtilis, B. clausii*, and *B. coagulans*. This cross-check was conducted to ensure the proper usage of probiotics or placebo in the experimental and control groups, respectively. The real-time PCR SYBR Green conditions were as follows: 95 °C for 10 min, amplification for 40 cycles at 95 °C for 15 s, 60 °C for 20 s, 72 °C for 30 s. The read-out standardization for *Bacillus* analysis was defined as C_t_ < 35 combined with a melting temperature (T_m_) of 85 ± 1 °C to confirm true positives.

### 16S rRNA vaginal metagenome analysis

The analysis of vaginal microbiome 16S rRNA was performed using next-generation sequencing (NGS) at Macrogene (Seoul, Korea) on the Illumina MiSeq (Illumina, San Diego, CA, USA) using a 2 × 250 bp run configuration. Five representative vaginal samples were collected from each group for 3 time points (*n* = 5 per group × 3 time points at days 0, 7 and 28, totalling 15 samples per group), resulting in a total of 30 samples for the two groups. The vaginal swab samples were selected randomly in a stratified manner based on age (≤30, >30), severity of bacterial vaginosis (mild/moderate and severe), and reproductive status (not yet given birth and has given birth). Total DNA was extracted from the vaginal swab samples using a Dneasy Mini Kit (Qiagen, Stockach, Germany). The V3–V4 hypervariable region of the 16S rRNA gene was amplified using the Herculase II Fusion DNA Polymerase Nextera XT Index Kit V2, and 300 bp paired-end DNA libraries were constructed using the 16S Metagenomic Sequencing Library Preparation kit. Sequencing of the 16S rRNA libraries was conducted by Macrogene Inc. (Seoul, Republic of Korea) on an Illumina MiSeq platform (Illumina, San Diego, CA, USA), with subsequent quality checking and base calling using Real Time Analysis (RTA). The CD-HIT-out-Miseq package was utilized to trim fastq reads, filter out short reads, and remove noise sequences. Ambiguous and chimera reads were identified and removed using rDnaTools (PacBio, USA). The remaining reads were clustered into operational taxonomic units (OTUs) using a greedy algorithm with a cut-off level of 97% sequence similarity. Qiime version 1.9.1.1 package was employed for diversity analysis, including assessment of OTUs abundance and alpha rarefaction, taxonomy diversity, and beta diversity. A minimum of 50,000 reads per individual sample, along with QV20 quality scores exceeding 99%, was ensured to maintain robustness of subsequent analyses. All bioinformatic procedures were conducted by the technical service team at Macrogene Inc. (Seoul, Republic of Korea). Further microbiota analysis and visualization were performed using the R package microbiome.

### pH measurement of vaginal swab samples

The vaginal pH was measured using a calibrated hand-held pH meter (Hanna Instrument, Japan). 100 µL of the collected vaginal fluid was applied onto the pH meter electrode, and the pH value was recorded. The measurement was performed in triplicate to ensure accuracy, and the average value was calculated for analysis.

### Data analysis

The safety and efficacy of LiveSpo X-Secret were evaluated and compared to 0.9% NaCl physiological saline using various clinical and sub-clinical criteria in both the X-Secret and Control groups. The criteria were as follows: (i) the percentage of patients experiencing typical symptoms and associated symptoms of BV at check-up time points, (ii) reduction levels (2^∆Ct^) of pathogen load, where ∆C_t_ was calculated as C_t_ (threshold cycle) at days 3, 7, 14, and 28 minus C_t_ at day 0. The C_t_ of the internal control was adjusted to be equal among all samples, and (iii) changes in the density of phyla and genera taxonomy in the vaginal 16S rRNA metagenome.

The statistical analysis incorporated both Intention‑to‑Treat (ITT) and Per‑Protocol (PP) approaches to comprehensively assess treatment efficacy and account for potential attrition during follow‑up. The ITT analysis was pre‑specified to compare outcomes between the two randomized groups, including all participants regardless of protocol adherence or study completion, thereby preserving the benefits of randomization and minimizing bias from post‑randomization exclusions. Covariates included in adjusted analyses—such as age, history of prior episodes, and baseline disease severity—were pre-specified in the study protocol and Statistical Analysis Plan (SAP) prior to database lock. These variables were selected based on clinical relevance and the feasibility of consistent data collection across study sites, with the aim of reducing confounding while preserving the integrity of the randomized design. No post hoc variable selection was performed.

Individual medical records were collected, and the patient’s information was then gathered in a data set. The tabular analysis is performed on dichotomous variables using the χ2 test or Fisher’s exact test when the expected value of any cell is below five or continuity correction in case of zero frequency of any cell. Continuous variables were analysed using non‑parametric tests (Mann‑Whitney U for between‑group comparisons, Wilcoxon signed‑rank for within‑group comparisons) when normality assumptions were not met. For normally distributed continuous variables, unpaired t‑tests (between groups) and paired t‑tests (within groups) were applied. For between-group comparisons, a significance threshold of two-sided *p* < 0.05 was used. For within-group comparisons at two pre-specified primary timepoints (Day 7 and Day 28), a Bonferroni-adjusted significance level of two-sided *p* < 0.025 was applied to account for multiple comparisons. Specific *p*-value and other details for each test are described in the figure legends. Statistical and graphical analyses are performed on GraphPad Prism v8.4.3 software (GraphPad Software, CA, USA), while advanced modeling (mITT) and microbiome diversity profiling were conducted in RStudio v4.0 (Vienna, Austria).

### Ethical issues

This study received ethics approval by Hanoi Obstetrics and Gynecology Hospital under Decision No 2257CNPS on November 17, 2023 and was conducted with the ethical principles in accordance with the Helsinki statement and the ICH GCP guidelines, in accordance with the Health Department’s current ethical regulations and standards on research on subject’s human. All patients who volunteered to participate in the study were given information about the study and signed an informed consent form. The study was registered with ClinicalTrials.gov in the US. National Library of Medicine, with Identifier No: NCT06165354 on December 08, 2023.

## Results

### Trial design and baseline demographic, clinical, and sub-clinical characteristics

A total of 223 participants were screened for eligibility from December 2023 to May 2024. Of these, 120 eligible women diagnosed with bacterial vaginosis were randomly assigned to one of two groups (*n* = 60 per each group): the standard care (Control) group and the probiotic-intervention (X-Secret) group (Fig. 1 – exclusion round 1). During the trial, participants in both groups received the same standard care treatment for bacterial vaginosis (Fig. 1 – follow-up, standard care treatment). This included oral administration of clindamycin for the initial 7 days to treat co-infection bacteria. If the oral treatments were unsuccessful at the visit check-up on day 3, a vaginal suppository administration of combined metronidazole and chloramphenicol was applied starting from day 3 for an additional 4 days. The distribution of patients receiving oral antibiotics or a combination of oral and vaginal suppository antibiotics was not significantly different between the X-Secret and Control groups (79.17% in Control vs. 87.5% in X-Secret for oral clindamycin; 20.83% in Control vs. 12.5% in X-Secret for combined oral clindamycin with vaginal suppository metronidazole and chloramphenicol (Canvey); *p* = 0.2733) (Supplementary Table 6). The only difference between the two groups was that patients in the Control group received a vaginal spray containing only physiological saline (NaCl 0.9%), while those in the LiveSpo X-Secret group received a vaginal spray containing physiological saline (NaCl 0.9%) plus *Bacillus* spores at 1 billion CFU/mL of *B. subtilis*, *B. clausii*, and *B. coagulans* (LiveSpo X-Secret). The frequency of vaginal-spray was three times daily for continuous 28 days of follow-up. During the follow-up period, which included five check-ups for clinical symptoms and sub-clinical assessments on days 0, 3, 7, 14, and 28, 12 participants from both the Control and X-Secret groups were excluded. This left 48 participants in each of the Control and X-Secret groups for the final analysis (Fig. 1 – exclusion round 2).

As detailed in Table 1, there were no significant differences in any indices regarding the demographic, clinical, and sub-clinical characteristics of women with BV between the Control and X-Secret groups at baseline for either the ITT population (all enrolled participants) or the PP population (participants completing the study without major protocol deviations). Baseline demographic characteristics such as age, education level, marital status, background BV history, intimate history (male partners), obstetric history, vaccination history, vaginal douching habits, and use of feminine hygiene products were comparable between the two groups (all *p*-values > 0.05). Additionally, baseline clinical symptoms including odor, itching, burning micturition, lower abdominal pain, abnormal vaginal discharge, and vaginal pH were generally similar (all *p*-values > 0.05). Furthermore, real-time PCR results showed a slight difference, albeit not statistically significant (all *p*-values > 0.05) in the presence of BV pathogens, such as *Gardnerella vaginalis* and *Mycoplasma hominis*.

**Table 1:** Demographic, clinical and sub-clinical characteristics of women with BV before treatment

Baseline characteristics	Intention-To-Treat (ITT) analysis		Per-protocol (PP) analysis	
	Control (*N* = 60)	X-Secret (*N *= 60)	Control (*N *= 48)	X-Secret (*N* = 48)
***Age, mean (SD)***	36.72 ± 9.87	36.3 ± 10.16	36.98 ± 10.23	37.67 ± 9.72
***Education level***, *n* (%)				
Secondary	16 (26.67)	12 (20.00)	16 (33.33)	9 (18.75)
University	35 (58.33)	39 (65.00)	25 (52.08)	32 (66.67)
Other	9 (15.00)	9 (15.00)	7 (14.58)	7 (14.58)
***Marital status***, *n* (%)				
Single	9 (15.00)	8 (13.33)	8 (16.67)	6 (12.5)
Married	50 (83.33)	50 (83.33)	39 (81.25)	40 (83.33)
Divorced	1 (1.67)	2 (3.33)	1 (2.08)	2 (4.17)
***Background BV history****, n* (%)				
Yes	6 (10.00)	6 (10.00)	4 (8.33)	5 (10.42)
No	54 (90.00)	54 (90.00)	44 (91.67)	43 (89.58)
***Intimate history*** (Male partners) *n* (%)				
0	6 (10.00)	7 (11.67)	5 (10.42)	5 (10.42)
1	54 (90.00)	53 (88.33)	43 (89.48)	43 (89.58)
***Obstetric history***, *n* (%)				
Haven’t given birth yet	12 (20.00)	9 (15.00)	10 (20.83)	5 (10.42)
1 child	9 (15.00)	12 (20.00)	4 (8.33)	7 (14.58)
2 children	32 (53.33)	34 (56.67)	28 (58.33)	31 (64.58)
>2 children	7 (11.67)	5 (8.33)	6 (12.5)	5 (10.42)
***History of hormonal drugs***, *n* (%)				
Yes	6 (10.00)	4 (6.67)	6 (12.5)	4 (8.33)
No	54 (90.00)	56 (93.33)	42 (97.5)	44 (91.76)
***Vaccination history (HPV vaccine)***, *n* (%)				
Yes	4 (6.67)	3 (5.00)	2 (4.17)	3 (6.25)
No	56 (93.33)	57 (95.00)	46 (95.83)	45 (93.75)
***Contraceptive***, *n* (%)				
None	37 (61.67)	36 (60.00)	25 (52.08)	27 (56.25)
Condoms	8 (13.33)	8 (13.33)	8 (16.67)	5 (10.42)
Birth control pills	7 (11.67)	9 (15.00)	7 (14.58)	9 (18.75)
Contraceptive implant	4 (6.67)	5 (8.33)	4 (8.33)	5 (10.42)
Intrauterine device	4 (6.67)	2 (3.33)	4 (8.33)	2 (4.17)
***Vaginal douching habit***, *n* (%)				
Yes	11 (18.33)	8 (13.33)	8 (16.67)	5 (10.42)
No	49 (81.67)	52 (86.67)	40 (83.33)	43 (89.58)
***Use feminine hygiene****, n* (%)				
Yes	42 (70.00)	40 (66.67)	30 (62.5)	28 (58.33)
No	18 (30.00)	20 (33.33)	18 (37.5)	20 (41.67)
***Clinical symptoms****, n* (%)				
Odor	41 (68.33)	44 (73.33)	29 (60.42)	32 (66.67)
Abnormal vaginal discharge	46 (76.67)	50 (83.33)	34 (70.83)	38 (79.17)
Vaginal pH*, n* (%)				
> 4.5	37 (61.67)	36 (60.00)	28 (58.33)	28 (58.33)
3.8 – 4.5	23 (38.33)	24 (40.00)	20 (41.67)	20 (41.67)
Itching	33 (55.00)	41 (68.33)	24 (50)	32 (66.67)
Burning micturition	6 (10.00)	7 (11.67)	5 (10.42)	6 (12.5)
Lower abdominal pain	6 (10.00)	6 (10.00)	6 (12.5)	6 (12.5)
***BV-associated bacteria****, n* (%)				
*G. vaginalis*	60 (100)	58 (96.67)	48 (100)	46 (95.83)
*G. vaginalis & M. hominis*	0 (0.00)	2 (3.33)	0 (0.00)	2 (4.17)

### Safety and symptomatic-relieving effects of vaginal-spray *Bacillus* Spores

Safety of the vaginal-spray probiotics was evaluated in patients on days 3, 7, 14, and 28 and patients in both groups were required to report any adverse reactions. During the 28-day follow-up period, no cases of allergic reactions or other complications were recorded with X-Secret group. No patient reported any abnormalities in their daily activities or experienced discomfort during the application process. The data indicated that vaginal-spray *Bacillus* spores LiveSpo X-Secret is safe and comfortable for patients.

**Fig. 2:**
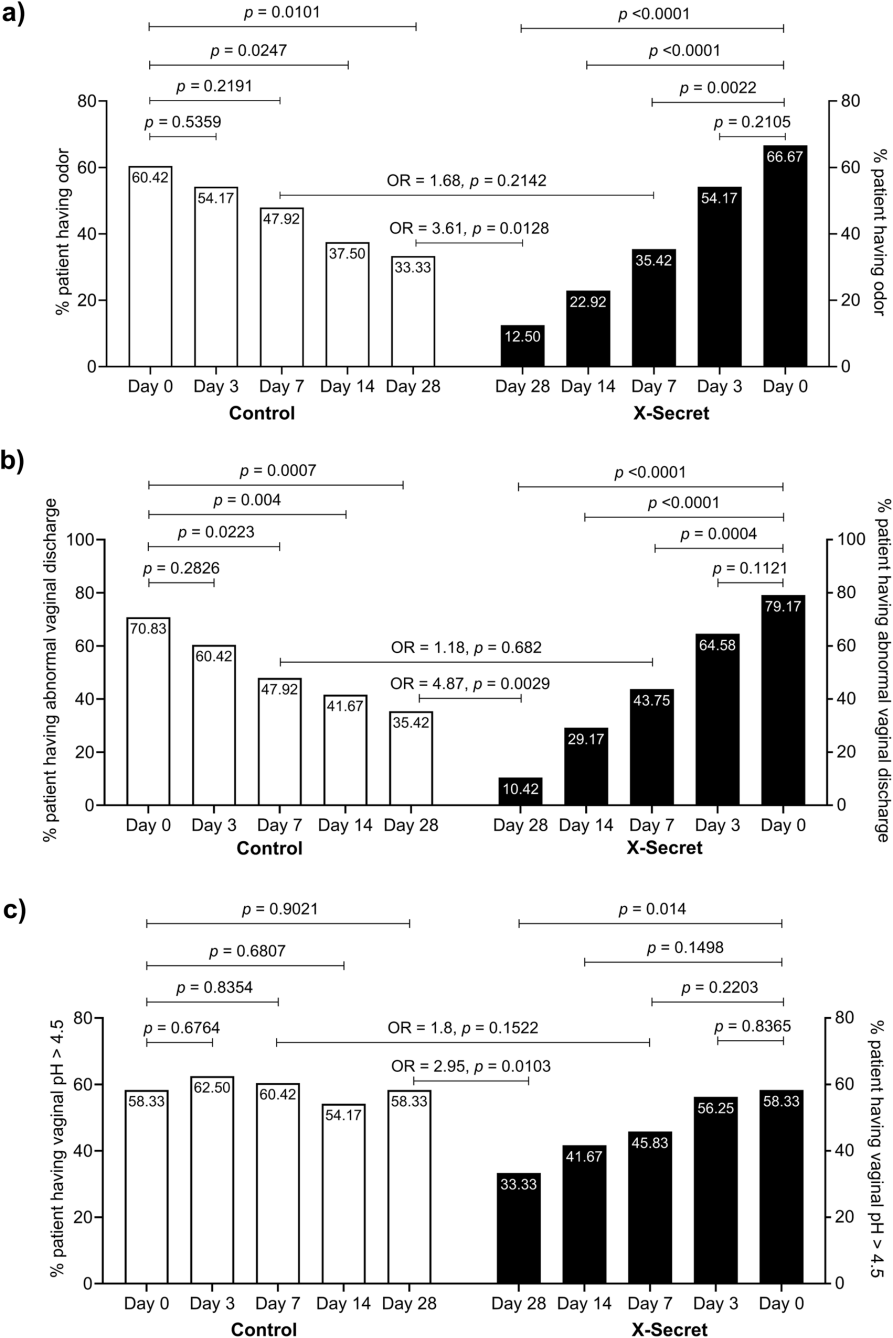
Percentage of patients (%) in the Control and X-Secret groups still experiencing typical BV symptoms at check-up time points at days 0, 3, 7, 14, and 28. **a** Bad odor, **b**Abnormal discharge and **c** Vaginal pH > 4.5. The two-sided Mann–Whitney test or Wilcoxon test was used to calculate the median difference of these symptoms in the two groups/ each group. 95% CI for median in each group and the median difference between the two groups were shown in (**a**), (**b**), and (**c**). The significance level of all analyses was set at the *p* < 0.05 and adjusted to 0.025 for two main time points (days 7 and 28) within the same group. Sample size: *n*  =  48 biologically independent samples per group (Control and X-Secret).

To examine the effectiveness of LiveSpo X-Secret, we conducted PP analysis to evaluate typical symptoms of bacterial vaginosis, including vaginal odor, abnormal vaginal discharge, and vaginal pH >4.5, in patients on days 3, 7, 14, and 28. We observed significant differences between the X-Secret and Control groups across these indications (Fig. 2). A reduction in the proportion of patients experiencing the three primary symptoms was observed as early as day 3 in both groups. However, this difference was not statistically significant (all *p*-values > 0.05) and only reached statistical significance from day 7 onward. The percentage of patients with vaginal odor in the X-Secret group was significantly reduced by 1.88-, 2.91- and 5.33-fold on days 7, 14, and 28, respectively, compared to day 0 (Fig. 2a - all *p*-values < 0.01). On the other hand, only 1.61- fold (*p* = 0.0247) and 1.81-fold (*p* = 0.0101) reductions on days 14 and 28, respectively, were observed in the Control group. At day 28, the likelihood of patients experiencing vaginal odor was significantly lower in the X-Secret group compared to the Control group, with an odds ratio of 0.28 (*p* = 0.0128), indicating a 3.61-fold reduction. The results indicate the effectiveness of LiveSpo X-Secret in reducing vaginal odor, with significant improvements obtained as early as 7 days after use and the most pronounced on day 28—three weeks after antibiotic discontinuation. Regarding abnormal vaginal discharge (Fig. 2b), X-Secret treatment was more effective in reducing the number of patients experiencing the symptom, with 1.81-, 2.71-, and 7.60-fold decreases on days 7, 14, and 28, respectively (all *p*-values < 0.001), compared to the Control group, which showed 1.48-, 1.70-, and 2.00-fold reductions on the same days (all *p*-values < 0.05). By day 28, the odds of patients having abnormal vaginal discharge were 4.87 folds lower in the X-Secret group compared to the Control group (*p* = 0.0029). Odds ratios were also calculated for other time points (days 3, 7, and 14) but the differences were not statistically significant (all *p*-values > 0.05). These data further indicate that the effectiveness of vaginal spray in improving the symptoms of abnormal vaginal discharge becomes evident after 7 days of use, with the most significant difference observed on day 28. Lastly, for the indicator of vaginal pH >4.5 (Fig. 2c), the X-Secret group showed an obvious decreasing trend over the treatment period, achieving a significant 1.75-fold reduction in the percentage of cases with vaginal pH >4.5 by day 28 compared to day 0 (*p* = 0.014). In contrast, the Control group did not show a decrease in pH levels. After 28 days, patients in the X-Secret group were 2.95 folds (OR = 2.95) less likely to have a vaginal pH > 4.5 compared to those in the Control group (*p* = 0.0103). Overall, these primary outcomes demonstrated that LiveSpo X-Secret reduces typical symptoms of BV, with noticeable efficacy observed from day 7 of antibiotic treatment and becoming more pronounced after day 28, 3 weeks after completing the standard antibiotic regimen, even after applying alpha adjustment (*p* < 0.025).

**Fig. 3:**
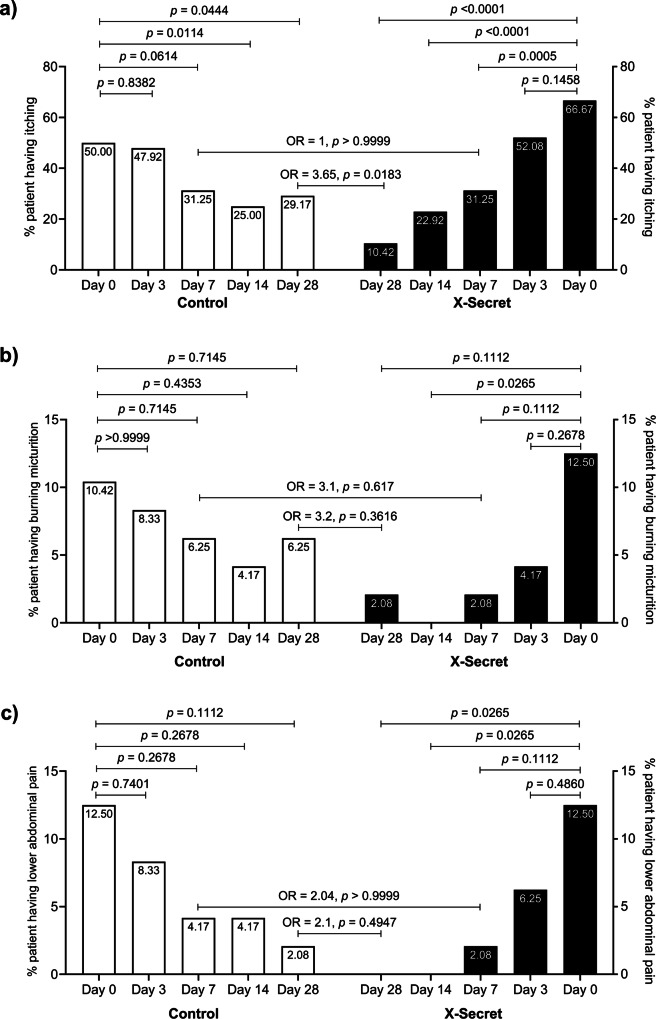
Percentage of patients (%) in the Control and X-Secret groups still experiencing symptoms associated with BV at check-up time points at days 0, 3, 7, 14, and 28. **a** Itching, **b** Burning micturition, and **c** Abdominal pain. The two-sided Mann–Whitney test or Wilcoxon test was used to calculate the median difference of these symptoms in the two groups/ each group. 95% CI for median in each group and the median difference between the two groups were shown in (**a**), (**b**), and (**c**). The significance level of all analyses was set at the *p* < 0.05 and adjusted to 0.025 for two main time points (days 7 and 28) within the same group. Sample size: *n*  =  48 biologically independent samples per group (Control and X-Secret).

In addition to the clinical symptoms, we also conducted PP analysis to evaluate the impact of vaginal-spray probiotics on improving the other discomforts associated with BV, including itching, burning micturition, and lower abdominal pain. We noted a consistent decrease in the percentage of patients exhibiting these three symptoms at days 3, 7, 14, and 28 compared to day 0. Nonetheless, this difference became statistically significant only in the X-Secret group from day 7 onwards, whereas the Control group did not show any statistically significant difference at any time point. First, as presented in Fig. 3a, the percentage of patients experiencing itching symptom in the X-Secret group was significantly reduced by 2.13-, 2.91-, and 6.40-fold, respectively, at days 7, 14, and 28 compared to day 0 (all *p*-values < 0.001). The Control group showed a significant reduction in itching symptoms only at days 14 and 28, but with a lower level of 2.00- and 1.71-fold decreases, respectively, compared to day 0 (all *p*-values < 0.05). Notably, the percentage of patients with itching in the Control group at day 28 (21 days post-antibiotic treatment) increased by nearly 4.17% compared to day 14 (7 days post-antibiotic treatment), indicating a small rate of reinfection. Odds ratios were analysed at all time points, but no significant differences were observed at days 3, 7, and 14 (all *p*-values > 0.05). However, at day 28, the odds ratio of patients in the X-Secret group experiencing itching were 3.65 folds lower compared to those in the Control group (*p* = 0.0183), highlighting the effectiveness of LiveSpo X-Secret in preventing re-occurrence of this symptom. Secondly, we observed a trend of reducing the percentage of patients experiencing burning micturition in both groups over follow-up 28 days (Fig. 3b). Although the percentage of patients with this symptom at specific time points, including days 3, 7, 14, and 28 was noticeably lower in the X-Secret group compared to the Control group, this difference was not statistically significant. A difference was observed only on day 14 compared to day 0 in the X-Secret group, with no patients experiencing the symptom on day 14, vs. 12.5% patients on day 0 (*p* = 0.0265). Similarly, we observed a reduction in the percentage of patients experiencing abdominal pain in both groups over the 28-day follow-up period. As shown in Fig. 3c, the percentage of patients with this symptom in the X-Secret group decreased rapidly, showing a 2-fold and 6-fold reduction on days 3 and 7, respectively, with no patients experiencing abdominal pain from day 14 onwards. In contrast, the Control group showed a slower reduction rate, with corresponding decreases of 1.5-, 3.0-, 3.0-, and 6.0-fold on days 3, 7, 14, and 28, respectively. For the two symptoms of burning micturition and abdominal pain, we found the odds ratios of experiencing these symptoms in the X-Secret group were lower compared to the Control group at all time points. Specifically, by day 28, the odds ratios were 3.2 and 2.1 folds lower, respectively. However, these differences were not statistically significant (*p* = 0.3616 and 0.4947).

In summary, the data (Figs. 2 and 3) supported the effectiveness of vaginal-spray probiotics LiveSpo X-Secret in alleviating typical BV symptoms within 7 days of use and preventing the recurrence of symptoms with continued use for up to 28 days. This highlights the advantages of LiveSpo X-Secret as a supportive treatment for the standard of care in reducing vaginal odor, abnormal vaginal discharge, vaginal pH, itching, burning micturition, and abdominal pain.

In addition to the above PP analyses, an ITT analysis using baseline‑carried‑forward (BCF) imputation for missing day‑3 symptom data was performed. Of 24 patients excluded from the PP set, only three had day‑3 data, and all were lost to follow‑up by day 7. Logistic regression showed no significant group differences for any of the five symptoms (all *p* > 0.3, Supplementary Table 7). Due to small sample size and high early dropout, ITT results were less reliable than PP estimates; therefore, ITT analysis was not applied to subsequent sub‑clinical outcomes.

### Reducing concentration of *Gardnerella vaginalis* with vaginal-spray *Bacillus* spore probiotics

**Fig. 4:**
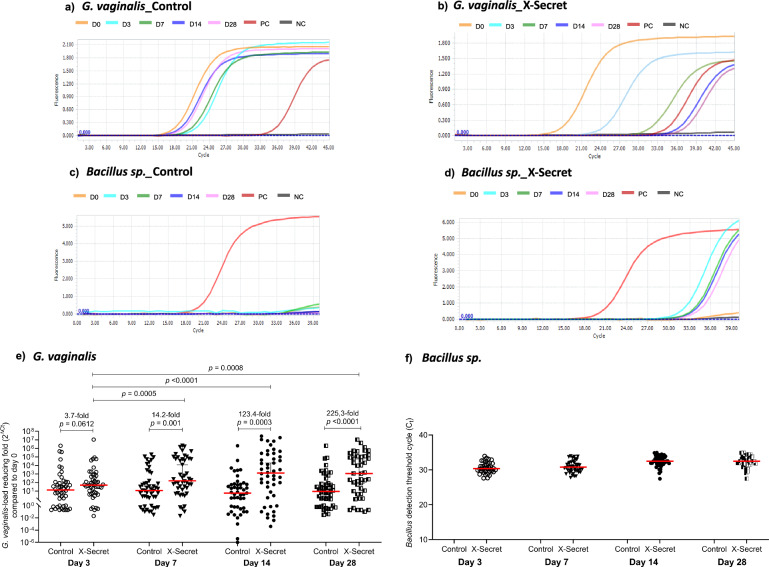
Changes in *G. vaginalis* concentrations and detection of *Bacillus* in vaginal swab samples of the Control and X-Secret groups at days 3, 7, 14, and 28 compared to day 0. **a–d** Multiplex real-time PCR TaqMan probe amplification curves specifically for *G. vaginalis* **a, b** and real-time PCR SYBR Green amplification curves targeted *Bacillus* sp. (*B. subtilis, B. clausii*, and *B. coagulans*) (**c, d**) taken from representative vaginal swab sample of Control and X-Secret groups at day 0 (D0), day 3 (D3), day 7 (D7), day 14 (D14) and day 28 (D28) of treatment. PC and NC represent positive and negative controls, respectively. (**e**) Reduction in *G. vaginalis* load, expressed as fold-change (2^△Ct^), in vaginal swab samples from Control and X-Secret groups at D3, D7, D14, and D28 compared to D0. **f** *Bacillus* sp. detection based on C_t_ values in vaginal swab samples of Control and X-Secret groups over the time. Statistical significance was assessed using the two-sided Mann–Whitney or Wilcoxon test. The 95% confidence interval (CI) for the median within each group and the median difference between groups are shown in (**e**). A significance level of *p* < 0.05 was applied to all analyses and adjusted to 0.025 for two main time points (days 7 and 28) within the same group (**e**). Sample size: *n*  =  48 biologically independent samples per group (Control and X-Secret) (**e**, **f**).

To evaluate the effectiveness of LiveSpo X-Secret in reducing anaerobic microorganisms associated with BV, we performed real-time PCR using TaqMan probes to semi-quantify changes in *Gardnerella vaginalis* concentration in vaginal swab samples. The assessment serves as a secondary outcome of this study. The fold change was calculated using 2^△Ct^ values, where ΔC_t_ represents the difference between the threshold cycle (C_t_) at days 3, 7, 14, and 28 compared to the C_t_ at day 0. Significant reductions were observed in the X-Secret group compared to the Control group at all time points. By day 3, the bacterial load in the X-Secret group showed a substantial decrease, which continued to improve over days 7, 14, and 28. Specifically, as shown in the representative amplification curves, the Control group exhibited only minor reductions in *G. vaginalis* concentration over time (Fig. 4a), whereas the X-Secret group showed a marked decrease at each time point compared to day 0 (Fig. 4b). The 2^△Ct^ values demonstrated that the bacterial load in the X-Secret group was reduced by 49-, 158.5-, 1117-, and 1284-fold at days 3, 7, 14, and 28, respectively, compared to a 13.2-, 11.15-, 9.05-, and 5.7-fold reduction in the Control group (Fig. 4e). Considering the comparative effectiveness of reducing *G. vaginalis* concentration, LiveSpo X-Secret exhibited 3.7 folds more effective than physiological saline just after 3 days of treatment. This effectiveness increased over time, significantly reaching 14.2-fold by day 7, 123.4-fold by day 14, and 225.3-fold by day 28. This indicates that the vaginal-spray probiotics significantly enhanced the clearance of *Gardnerella vaginalis* in the vagina during and after the standard care treatment. For *Mycoplasma hominis*, we could not assess changes in their concentration due to the limited number of infected cases detected at day 0, which prevented statistical analysis.

To ensure the accurate administration of LiveSpo X-Secret and the control treatment, we confirmed the presence of *Bacillus* species, including *B. subtilis, B. clausii*, and *B. coagulans*, in vaginal samples at the five time points using real-time PCR SYBR Green detection. While there were no detectable signals in the Control group at any time points (Fig. 4c), fluorescent signals corresponding to *Bacillus* sp. (including *B. subtilis, B. clausii*, and *B. coagulans*) genomic DNA were readily detected in the X-Secret group at days 7, 14, and 28, but not at day 0 (Fig. 4d). As a result, C_t_ values for *Bacillus* fluorescence signals were undetectable in the Control group at all time points and in the X-Secret group at day 0. However, in the X-Secret group, *Bacillus* was detectable at days 3, 7, 14, and 28, with median C_t_ values ranging from 30 to 34 (Fig. 4f). These findings confirm the compliance of participants with placebo and probiotic administration in our trial, and the presence of *Bacillus* in vaginal samples of participants receiving LiveSpo X-Secret.

### Enhancement of vaginal microbiota upon vaginal-spray *Bacillus* spore probiotics

The vaginal microbiota plays a crucial role in maintaining vaginal health. To evaluate the impact of the *Bacillus* spore probiotic LiveSpo X-Secret on vaginal microbiota, we measured the changes in alpha and beta diversity, and representation of the phylum, genus, and species levels of 16S rRNA metagenome of vaginal fluid. In each group, representative patient samples were randomly selected from 48 patients based on stratified criteria including age, severity of vaginitis, and reproductive status (*n* = 5 for each group x 3 time points at day 0, day 7, and day 28). Both days 7 (end of the antibiotic regimen) and 28 (21 days post-treatment) were key follow-up time points when significant differences between the Control and X-Secret groups in reducing BV symptoms and *G. vaginalis* concentration were observed.

Regarding the alpha diversity of vaginal discharge samples before and after the intervention, we did not observe any consistent trends in the four indices: Observed OTU, Chao1, Shannon, and Simpson (Supplementary Fig. 8a). Similarly, beta diversity analysis using PCoA revealed significant overlap among all tested samples (Supplementary Fig. 8b). The alpha and beta analysis data did not indicate a clear enhancement in the richness and diversity of the vaginal microbiota during the probiotic treatment time, and showed no substantial difference in the vaginal microbiota composition between the Control and X-Secret groups.

**Fig. 5:**
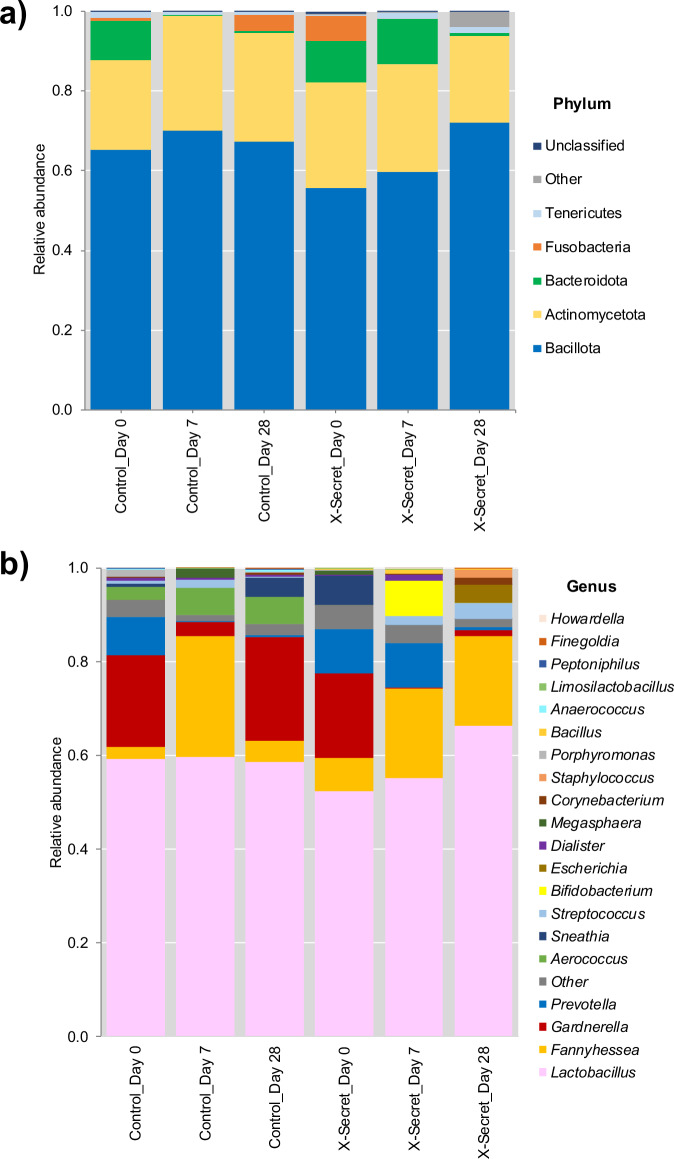
Analysis of 16S rRNA vaginal microbiota showing the distribution of major phyla and genera in the Control and X-Secret groups at day 7 and day 28 compared to day 0. **a** Relative abundance of phyla, **b** Relative abundance of genera.

We then further examined the distribution of 16S rRNA microbial components to comprehensively characterize the phylum, genus, and species of microorganisms present in vaginal discharge samples from the Control and X-Secret groups. The Fig. 5 illustrates the relative abundance of bacterial phyla (a) and genera (b) in patients with bacterial vaginosis before intervention and after 7 and 28 days of treatment. The results identified three main phyla: Actinomycetota, Bacillota, and Bacteroidota. Among these, Bacillota exhibited the highest relative abundance, with a consistent increase observed only in the X-Secret group at days 7 and 28-showing 1.07- and 1.29-fold increases, respectively, compared to day 0. The Control group showed only minor increases of 1.07 and 1.03 folds at days 7 and 28 compared to day 0. Beside Bacillota, Actinomycetota emerged as one of the dominant phylum in the vaginal microbiota at days 7 and 28 in both groups. While the density of this phylum increased by about 1.2 folds in the Control group at both days 7 and 28, it just slightly increased at day 7 (1.03 folds) and even decreased to 0.8-fold at day 28 in the X-Secret group. On the other hand, the relative abundance of Bacteroidota decreased in both groups to a density of 10%, which was maintained solely in the X-Secret group on day 7. Significant phylum differentiation was observed on day 28, where the Control group showed an increase in Fusobacteria from 0.8% to 4.1%, while the X-Secret group exhibited an increase in Tenericutes from undetectable levels to 2%.

At the genus level, four predominant genera were identified: *Lactobacillus, Gardnerella, Prevotella*, and *Sneathia*. *Lactobacillus* showed no significant change in the Control group across days 0, 7, and 28, with proportions of 59%, 60%, and 59%, respectively. However, the X-Secret group demonstrated a gradual increase, with increments of 6% and 27% on day 28 compared to day 0. Other genera, *Gardnerella, Sneathia*, and *Prevotella*, known for containing many harmful species in the vaginal microbiota, showed a dramatic decrease on day 28 in the X-Secret group, specifically decreasing 14.3, 806.6, and 13.3 folds, respectively. There was also an increase in the potentially harmful genus *Fannyhessea* from 7% to 19%, but this increment remains within the normal range (≤25%) according to previous studies^29^. Conversely, the Control group experienced a more disadvantageous outcome on day 28, with the recurrence of *Gardnerella* increasing 1.13 folds (from 19.6% to 22.3%), despite a 6.4-fold decrease on day 7 compared to day 0. Similarly, the recurrence of *Sneathia* on day 28 increased 5.4 folds compared to day 0, despite being undetectable on day 7. *Prevotella* decreased 26.7 folds on day 28 compared to day 0. Additionally, an abnormal increase in *Fannyhessea* to over 25% was noted in the Control group on day 7, but this proportion decreased to 4% on day 28. The level was below the normal range and no studies has reported the impact of such low relative abundance. In the X-Secret group, we observed positive changes in the genera *Streptococcus, Bifidobacterium*, and *Staphylococcus*. *Streptococcus* increased 58.2 and 109.9 folds on days 7 and 28, respectively, compared to day 0, while *Staphylococcus* increased 43.6 folds on day 28. *Bifidobacterium* increased 615.4 folds on day 7, although it decreased sharply on day 28. These beneficial changes were not observed in the Control group. The X-Secret group exhibited a significant increase in beneficial bacteria and a decrease in harmful bacteria phyla and genera, suggesting that the treatment effectively promoted a healthier vaginal microbiota.

**Fig. 6:**
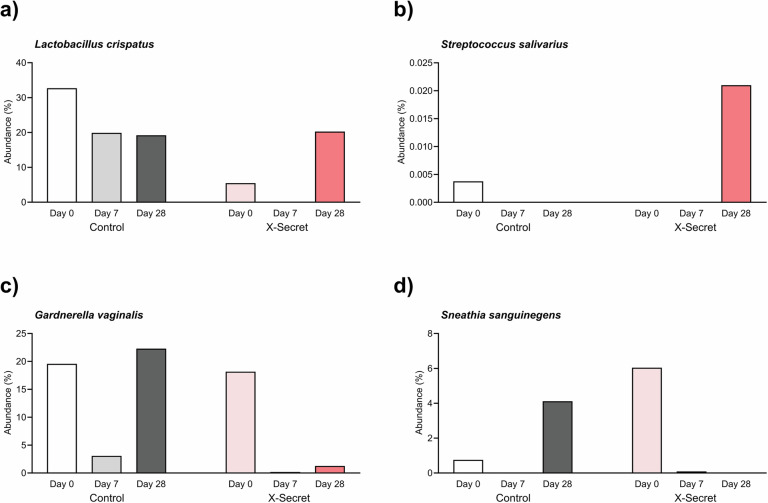
Average relative abundance of representative species in vaginal samples of Control and X-Secret groups at day 7 and day 28 compared to day 0. **a, b** Beneficial species. **c, d** Harmful species.

The relative abundance of four high-density vaginal microbiota species in patients with BV was assessed at three time points: day 0, day 7, and day 28, and the data was presented in Fig. 6. First, we focused on analysing beneficial species. For *L. crispatus*, the Control group showed a 1.7-fold decrease on days 7 and 28. In contrast, the X-Secret group exhibited an initial decrease on day 7, followed by a 3.71-fold increase on day 28. *Streptococcus salivarius* was undetectable in the Control group on days 7 and 28, starting from an initial 0.38%, whereas the X-Secret group showed an increase, reaching 2.1% on day 28 compared to day 0. Regarding harmful species, *G. vaginalis* demonstrated a trend of reduction, decreasing 95-fold at day 7 (*p* = 0.0556) and 14.29-fold at day 28 (*p* = 0.0635) in the X-Secret group compared to day 0. The Control group, treated with conventional antibiotics, showed a 6.4-fold decrease on day 7, but the abundance increased 1.14-fold on day 28 compared to day 0. *Sneathia sanguinegens* followed a similar pattern to *G. vaginalis* in the Control group, with a 5.44-fold increase on day 28 compared to day 0, whereas the X-Secret group showed a dramatic reduction of 806-fold on day 28. Taken together, these findings indicate that the X-Secret treatment significantly improves the vaginal microbiota, promoting the recovery and growth of beneficial species while substantially reducing harmful species, in contrast to the Control group which showed mixed results post-antibiotic treatment.

## Discussion

*Bacillus* spore probiotics can be formulated into a highly purified and concentrated liquid suspension, suitable for direct spraying into the vaginal tract. Data from this clinical trial demonstrated the safety of LiveSpo X-Secret for all participants. No adverse reaction was reported in the X-Secret group throughout the 28-day follow-up period. This finding is consistent with previous studies that have shown the safety profile of probiotic treatments in gynecological applications^17–20,23–25,30,31^.

Administration of LiveSpo X-Secret via vaginal spraying three times daily resulted in improved treatment efficacy and a reduction in symptom recurrence compared to the Control group. The proportion of patients still experiencing BV symptoms, including bad odor, abnormal vaginal discharge, and vaginal pH > 4.5, itching, burning micturition, and abdominal pain, generally tended to be lower in the X-Secret group compared to the Control group at the same follow-up time points. This difference was noticeable as early as day 7 of treatment and became significantly pronounced in preventing the recurrence of the four symptoms after 28 days of use. Specifically, the odds ratios were 3.61 for vaginal odor, 4.87 for abnormal vaginal discharge, 2.95 for vaginal pH > 4.5, and 3.34 for itching. When compared to previous clinical studies using both oral and suppository probiotics, whether they utilized *Lactobacillus* or *Bacillus* strains^17–19,23–25,30,32,33^, our study stands out for its faster effectiveness within initial 7 days during antibiotic treatment and even more pronounced improvement over 28 days follow-up. For example, in the most recent and well-designed clinical study by Vivekanandan et al. (2024)^23^, there was only a 76.1–82.6% improvement in the number of cases cured of symptoms such as vaginal odor, abnormal vaginal discharge, and vaginal pH > 4.5 after 28 days of treatment with suppository probiotics containing combined strains of *Lactobacillus crispatus* Bi16, *L. gasseri* Bi19, *B. coagulans* Bi34, and *L. acidophilus* Bi14 (VagiBIOM). In addition to the aforementioned typical symptoms of BV, the efficacy in improving other BV-associated symptoms, including itching, burning micturition, and abdominal pain, in the probiotic intervention group was achieved very quickly, with statistically significant differences observed from day 7 (for itching) or day 14 (for burning micturition and abdominal pain). Notably, our study shows a lower reinfection rate after a 28-day follow-up in the X-Secret group for these three symptoms compared to the Control group, particularly with odds ratios of 3.34 for the itching symptom. This suggests the enduring protective effects of vaginal-spray *Bacillus* spore probiotics against BV. Meanwhile, these three BV-associated symptoms in most of previous clinical studies did not yield significantly different results compared to our study. In addition to the potentially lower effectiveness of probiotics administered orally or via suppositories in those studies, this could also be attributed to the smaller sample sizes of these studies on symptoms, resulting in statistically insignificant differences^17–19,23–25,30,32^. Taken together, our study highlights the potential of *Bacillus* spore-based vaginal-spray probiotics, such as LiveSpo X-Secret, for rapid and effective relief of BV. LiveSpo X-Secret demonstrated efficacy in reducing six BV symptoms and preventing reinfection. While standard BV treatments like metronidazole and clindamycin are effective short-term, they often result in high recurrence rates (50-70% within six months). LiveSpo X-Secret not only significantly reduces symptoms during antibiotic therapy but also potentially lowers recurrence rates post-therapy, making it a promising alternative or adjunctive treatment for BV.

In this study, we did not assess changes in the Nugent score, as typically conducted in other studies. This is due to its accuracy being potentially compromised by the presence of *Bacillus* species in the vaginal environment. Instead, we reported the presence of BV-related *G. vaginalis* and *M. hominis*, and focused on measuring changes in the concentration of *G. vaginalis* - the pathogen detected most frequently in BV patients - using real-time PCR TaqMan probe assay, which screens for nine common pathogens and shows higher reliability. Notably, LiveSpo X-Secret demonstrated a 37-fold greater reduction in *G. vaginalis* concentration compared to the Control group just after 3 days of treatment, increasing to 225.3-fold greater by day 28. Compared to previous studies on *Lactobacillus*-based probiotics, LiveSpo X-Secret demonstrates superior results in reducing *G. vaginalis* concentration. For example, Park et al. (2023) evaluated the effectiveness of an oral probiotics MED-01, which contains five probiotic strains: *Ligilactobacillus salivarius* MG242, *Limosilactobacillus fermentum* MG901, *L. plantarum* MG989, *Lacticaseibacillus paracasei* MG4272, and *L. rhamnosus* MG4288 at a concentration of 5 × 10^9^ CFU/capsule, taken as one capsule daily, in women with BV^34^. The results showed that after three months of continuous use, the concentration of *G. vaginalis* in the probiotic intervention group decreased compared to the Control group, but the difference was not statistically significant. Our data suggests that vaginal-spray *Bacillu*s spores may provide enhanced efficacy in pathogenic inhibition due to their in-situ administration. This may allow the *Bacillus* spores to temporarily colonize and germinate, thereby exhibiting antimicrobial activity directly in the vaginal environment.

Our profiles of vaginal microbiota by 16S rRNA metagenome analysis provided significant insights and demonstrates promising results. In the X-Secret group, *Lactobacillus* genus increased significantly, while harmful genera like *Gardnerella, Sneathia*, and *Prevotella* decreased by day 28. Beneficial genera such as *Streptococcus* and *Bifidobacterium* also increased significantly in the X-Secret group. At the species level, beneficial species like *L. crispatus* and *S. salivarius* increased, whereas harmful species like *G. vaginalis* and *S. sanguinegens* decreased significantly in the X-Secret group. These changes were not observed in the Control group. Overall, LiveSpo X-Secret effectively enhances vaginal microbiota by increasing beneficial bacteria and reducing harmful bacteria, suggesting it as a promising alternative or adjunctive treatment for vaginal health. The reduction in *G. vaginalis* density measured by metagenome sequencing analysis of the V3–V4 hypervariable region of the 16S rRNA gene was found to be one-tenth to one-hundredth of that observed with real-time PCR targeting a specific *tuf* sequence for *G. vaginalis*. The real-time PCR results are likely more accurate due to the higher quantitative reliability of the technology and a sample size that includes all patients. In contrast, the 16S rRNA metagenome analysis was performed on only five representative samples. While the latter reflects the trend of changes, generalisation of the observed trends to the entire population is limited. Nevertheless, our findings of vaginal microbiota restoration, based on 16S rRNA metagenome data, align with those of Vivekanandan et al. (2024), who observed improvements in vaginal microbiota following *Lactobacillus*-based probiotic intervention via vaginal suppository administration^23^. They noted that the probiotic group exhibited increased levels of beneficial genera such as *Streptococcus* and *Bifidobacterium* by day 28, whereas these increases were not seen in the Control group. Specifically, beneficial species like *Lactobacillus delbrueckii*, *L. vaginalis*, and *L. salivarius* showed significant increases in the probiotic group. Conversely, harmful genera including *Gardnerella, Sneathia*, and *Prevotella* decreased significantly in the probiotic group by day 28, contrasting with increases observed in the Control group. At the species level, *Gardnerella vaginalis* and *Sneathia sanguinegens* exhibited notable reductions in the probiotic group, whereas these species increased in the placebo group following antibiotic treatment. In sum, the primary data of our 16S rRNA vaginal metagenomic analysis suggest the potential of *Bacillus* spores in providing enhanced and expedited benefits for vaginal health, particularly in re-establishing a healthy vaginal microbiota and reducing pathogenic bacteria. Although *Bacillus* spores are not native to the vaginal microbiota and do not lead to long-term colonization, our study captured their presence at all follow-up time points up to 28 days. This suggests their potential role in modulating microbial interactions by suppressing *G. vaginalis* and other BV-associated anaerobes, thereby supporting BV treatment and microbiota restoration. These findings highlight a novel therapeutic approach that differs from *Lactobacillus*-based probiotics, warranting further investigation.

While reporting encouraging results supporting the use of vaginal spray of *Bacillus* spore probiotics, this study also has several limitations. First, it was conducted in a specific geographic region, which may not fully reflect potential variations in BV prevalence and vaginal microbiota composition across different racial and ethnic groups. Given that BV rates and microbiota profiles can differ by race, ethnicity, and geographic locations, future studies should include participants from diverse backgrounds to ensure a more comprehensive evaluation of the efficacy of *Bacillus* spore probiotics in supporting BV treatment and preventing recurrence. Additionally, the high cost of metagenomic sequencing and the requirement to analyse multiple time points for each sample limited the sample size given the provided budget. We acknowledge this limitation in the current analysis to reinforce that the results are exploratory. Future trials with sufficient power will be needed to confirm these findings and apply proper adjustments for multiplicity across timepoint. Future research should include larger sample sizes to thoroughly evaluate the effects of the combined *B. subtilis, B. clausii*, and *B. coagulans* spore probiotics on the changes in vaginal microbiota and to incorporate covariates or employ more sophisticated models. Furthermore, this study primarily focused on evaluating the clinical and microbiological effectiveness of probiotics in supporting BV/symptom treatment during antibiotic therapy and in reducing recurrence after completing antibiotic treatment. While long-term follow-up to assess BV recurrence after completing vaginal-spray probiotic use was not within the study design, the findings suggest potential benefits of daily vaginal-spray probiotic use. These findings are hypothesis-generating and should be interpreted with caution. The sample size was selected based on prior power calculations approved by the ethics committee, and while the study provides preliminary evidence of efficacy, larger trials are needed to validate these results and assess long-term outcomes.

In conclusion, to the best of our knowledge, this is the first study to demonstrate the efficacy of vaginal-spray probiotics in improving vaginal health by reducing pathogenic *G. vaginalis* concentrations and enhancing the composition of the vaginal microbiota. The vaginal-spray probiotic LiveSpo X-Secret is both safe and effective for managing symptoms of bacterial vaginosis. It significantly reduces vaginal odor, abnormal vaginal discharge, high vaginal pH, and itching, while also lowering the reinfection rate, making it a promising option for supportive treatment and daily-use to reduce recurrence. These results extend previous findings on probiotics in gynecological health, highlighting vaginal-spray *Bacillus* spores as a robust and effective in-situ therapeutic agent with rapid and substantial improvements in vaginal microbiota composition compared to *Lactobacillus*-based oral probiotics and standard antibiotic therapy without probiotic supplements. These findings support further clinical trials with larger sample sizes to evaluate its effectiveness in treating symptoms and preventing recurrence in women with bacterial vaginosis.

## Supplementary information

Supplementary Information

Description of Additional Supplementary Files

Supplementary data 1

Supplementary Data 2

Supplementary Data 3

## Data Availability

All source data for Figs. 1–6 can be accessed from Supplementary Data 3. 16S rRNA (accession no. for *B. subtilis* ANA46, *B. clausii* ANA39, and *B. coagulans* ANA40 were MT123906.1, MT275656.1, and MT734108, respectively, in NCBI) and whole genome sequencing analysis (deposited under project’s accession number PRJNA1099787 on NCBI) (Supplementary Figs. 1–6), and (iv) sequence analysis of antibiotic resistance and toxic genes in the whole genomes (Supplementary Tables 3–5). Additional materials, including the Study Protocol and Statistical Analysis Plan, as well as other data not included in these files are available from the corresponding author (vananhbiolab@gmail.com) upon reasonable request and with approval from the Spobiotic Research Center, ANABIO R&D Ltd.
