# Supplementary material for: Vaginal-spray Bacillus spore probiotics as a potential treatment and reducing recurrence of bacterial vaginosis: randomized, double-blind, and controlled pilot study
Source: Commun Med (Lond). 2025 Nov 18;5:527. doi: 10.1038/s43856-025-01236-4 (PMC12717237; doi:10.1038/s43856-025-01236-4)
Supplement: Supplementary file 1 — Supplementary Information [file 43856_2025_1236_MOESM1_ESM.pdf]

Supplementary Information

Vaginal-spray *Bacillus* spore probiotics as a potential treatment and reducing recurrence of bacterial vaginosis: randomized, double-blind, and controlled pilot study

Table of contents

Content	Page
1. Supplementary tables	2
2. Supplementary figures	8
3. CONSORT checklist	16

1. **Supplementary tables**

Supplementary Table 1. Microbiological and biochemical characterization of *B. subtilis* ANA46, *B. clausii* ANA39, and *B. coagulans* ANA40

**Characteristics**	***B. subtilis* ANA46**	***B. clausii* ANA39**	***B. coagulans* ANA40**
Sporulation efficiency (%)	97	90	90
Heat stability of spores (°C)	80	65	60
Width size of vegetative cell (μm)	< 1 μm	< 1 μm	< 1 μm
Amylase	++	+++	++++
Caseinase	+++	++++	-
Lipase	+	++	-
Catalase	+	+	+++
Gelatinase	+++	+	++
Optimal temperature (°C)	37	35	44
Optimal pH	7.5	8.0	6.5
6.5% NaCl, 50°C	+	-	-
Aerobic/Anaerobic	++++	+++	+++
Anerobic	++++	+++	+++
Hemolysis	γ (No)	γ (No)	γ (No)
VP Test	+	-	+
Closest match*	*B. subtilis* (100%)	*B. clausii* (99.5%)	*B. coagulans* (99.4%)

-, negative; +, weak or positive; ++, average; +++, good/high; ++++, very good/very high.

**Using 16S rDNA sequence analysis in this work. The similarity score is shown in brackets.*

Supplementary Table 2. Antibiotic susceptibility of *B. subtilis* ANA46, *B. clausii* ANA39, and *B. coagulans* ANA40

**Antibiotic discs (µg)***	***B. subtilis* ANA46**	***B. clausii* ANA39**	***B. coagulans* ANA40**
Ampicillin (10)	22.01 ± 0.4 (S)	27.04 ± 1.3 (S)	28.30 ± 0.21 (S)
Chloramphenicol (30)	26.01 ± 0.5 (S)	18.47 ± 0.20 (S)	30.11 ± 0.39 (S)
Ciprofloxacin (5)	26,11 ± 0.3 (S)	30.89 ± 0.61 (S)	25.10 ± 0.31 (S)
Clindamycin (2)	20.15 ± 0.3 (S)	0 (R)	25.12 ± 1.08 (S)
Cotrimoxazole (25)	28.60 ± 0.2 (S)	34.50 ± 0.95 (S)	25.71 ± 0.50 (S)
Erythromycin (15)	20.56 ± 0.5 (S)	0 (R)	22.60 ± 0.51 (S)
Gentamicin (10)	21.21 ± 0.39 (S)	28.22 ± 0.39 (S)	24.41 ± 0.31 (S)
Kanamycin (30)	21.30 ± 0.1 (S)	24.84 ± 0.04 (S)	26.4 ± 0.03 (S)
Neomycin (30)	17.44 ± 0.1 (S)	24.91 ± 0.13 (S)	21.40 ± 0.30 (S)
Rifampicin (30)	25.10 ± 0.2 (S)	39.06 ± 0.68 (S)	39.87 ± 0.31 (S)
Streptomycin (10)	13.45 ± 0.2 (I)	6.51 ± 0.46 (R)	16.20 ± 0.40 (S)
Tetracycline (30)	15.10 ± 0.1 (I)	27.99 ± 0.14 (S)	35.50 ± 1.10 (S)
Trimethoprim (5)	30.0 6± 0.4 (S)	39.83 ± 0.72 (S)	22.81 ± 0.40 (S)
Vancomycin (30)	16.13 ± 0.2 (S)	22.41 ± 0.22 (S)	20.41 ± 0.22 (S)

**Antibiotic-impregnated discs (6 mm) with amount in µg shown in brackets.*

+Diameter of inhibition zones from three individual experiments. S, sensitive; I, intermediate resistant; R, resistant.

Supplementary Table 3. Sequence analysis of antibiotic resistance genes in *B. subtilis* ANA46 genome

Resistance gene	% Identity	Query / Template length	Contig	Position in contig	Predicted phenotype	Accession number
addK	99.76	853/855	000000F |arrow	452124..452976	Aminoglycoside resistance	M26879
mph(K)	100	921/921	000000F |arrow	87052..97982	Macrolide resistance	NC_000964
tet(L)	96.79	1377/1377	000000F |arrow	37988..39364	Tetracycline resistance	D12567

Notes: The three genes including *addK* classified in Aminoglycoside antibiotic group, *mph(K)* classified in Macrolide antibiotic group, and *tet(L)* classified in Tetracycline antibiotic group were found. They may belong to acquired antibiotic resistant genes. The presence of the three genes *addK, mph(K)* and *tet(L)* are consistent with the streptomycin, and tetracycline resistant phenotype of *B. clausii* ANA46 indicated by the diffusion disc assay.

Supplementary Table 4. Sequence analysis of antibiotic resistance genes in *B. clausii* ANA39 genome

Resistance gene	% Identity	Query / Template length	Contig	Position in contig	Predicted phenotype	Accession number
ant(4')- Ib	98.83	771 / 771	000000F |arrow	81510..82280	Aminoglycoside resistance	AJ506108
erm(34)	96.04	833 / 846	000000F |arrow	1033445..1034277	Macrolide resistance	AY234334
cat	96.79	685 / 687	000000F |arrow	2589949..2590625	Phenicol resistance	AY238971

Notes: The three genes including *ant(4')-Ib* classified in Aminoglycoside antibiotic group, *erm(34)* classified in Macrolide antibiotic group, and cat classified in Phenicol antibiotic group were found. They may belong to acquired antibiotic resistant genes. The presence of the two genes *ant(4')-Ib* and *erm(34)* are consistent with the streptomycin and erythromycin resistant phenotype of *B. clausii* ANA39 indicated by the diffusion disc assay. Although *cat* gene is available in the genome, its expression level may be low so that the strain is still sensitive to chloramphenicol. Interestingly, clindamycin resistance gene was not found in the genome of *B. clausii* ANA39, suggesting that the strain is intrinsic resistance to clindamycin.

Notes for sequence analysis of antibiotic resistance genes in *B. coagulans* ANA40 genome

The strain *B. coagulans* ANA40 does not exhibit antibiotic resistance activity, does not contain antibiotic resistance genes in both the DNA genome and plasmid, as well as does not contain any genes for intestinal toxins or food allergies.

Supplementary Table 5. Sequence analysis of toxin genes in *B. subtilis* ANA46, *B. clausii* ANA39, and *B. coagulans* ANA40 genome

No	Gene	Specific sequence amplified by PCR			Number of gene detected by whole genome sequencing		
		***B. subtilis* ANA46**	***B. clausii* ANA39**	***B. coagulans* ANA40**	***B. subtilis* ANA46**	***B. clausii* ANA39**	***B. coagulans* ANA40**
1	Hemolysin B (*hbl*B)	ND	ND	ND	0	0	0
2	Non-hemolytic enterotoxin A (*nhe*A)	ND	ND	ND	0	0	0
3	*nhe*B	ND	ND	ND	0	0	0
4	*nhe*C	ND	ND	ND	0	0	0
5	Cytotoxin K (*cyt*K)	ND	ND	ND	0	0	0

Supplementary Table 6. Antibiotic regimen for BV treatment

**Antibiotic regimen**	**Control group N = 48**	**X-secret group N = 48**	***p-value***
Oral clindamycin *n* (%)	38 (79.17)	42 (87.50)	0.2733[b]
Combined oral clindamycin with vaginal suppositories of metronidazole and chloramphenicol (Canvey) *n* (%)	10 (20.83)	6 (12.50)	

[b]*Two-sided Chi-Square test. A significance threshold of $p < 0.05$ was applied.*

Supplementary Table 7. Odds ratios for symptom improvement in the X-Secret group compared to the Control group (ITT and PP Analyses)

	ITT analysis			PP analysis		
**Symptom**	**Odds Ratio (OR)**	**95% CI (Lower – Upper)**	***P*-value**	**Odds Ratio (OR)**	**95% CI (Lower – Upper)**	***P*-value**
**Odor**	0.93	(0.44 – 1.95)	0.85	1	(0.45 – 2.24)	1
**Itching**	1.07	(0.52 – 2.2)	0.855	1.18	(0.53 – 2.64)	0.683
**Burning**	0.47	(0.1 – 1.89)	0.307	0.48	(0.06 – 2.58)	0.408
**Abdominal pain**	0.74	(0.14 – 3.49)	0.698	0.73	(0.14 – 3.51)	0.696
**Vaginal discharge**	1.08	(0.5 – 2.36)	0.843	1.19	(0.52 – 2.75)	0.673

Notes: The binary logistic regression model was used to estimate odds ratios (OR) with 95% confidence intervals and corresponding two-sided p-values (Wald test).

2. Supplementary figures

a

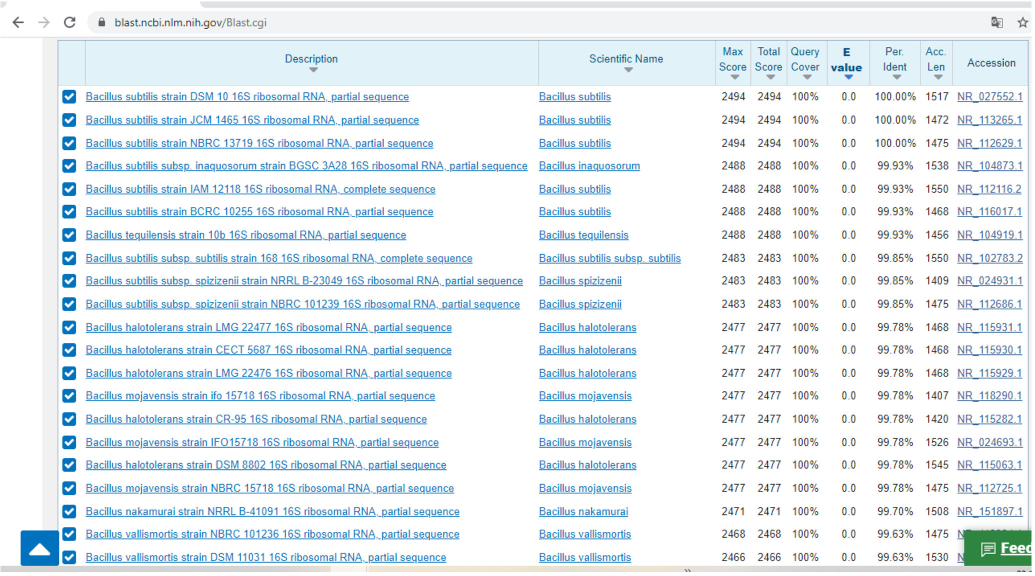

blast.ncbi.nlm.nih.gov/Blast.cgi

Description	Scientific Name	Max Score	Total Score	Query Cover	E value	Per. Ident	Acc. Len	Accession
Bacillus subtilis strain DSM 10 16S ribosomal RNA, partial sequence	Bacillus subtilis	2494	2494	100%	0.0	100.00%	1517	NR_027552.1
Bacillus subtilis strain JCM 1465 16S ribosomal RNA, partial sequence	Bacillus subtilis	2494	2494	100%	0.0	100.00%	1472	NR_113265.1
Bacillus subtilis strain NBRC 13719 16S ribosomal RNA, partial sequence	Bacillus subtilis	2494	2494	100%	0.0	100.00%	1475	NR_112629.1
Bacillus subtilis subsp. inaquosorum strain BGSC 3A28 16S ribosomal RNA, partial sequence	Bacillus inaquosorum	2488	2488	100%	0.0	99.93%	1538	NR_104873.1
Bacillus subtilis strain IAM 12118 16S ribosomal RNA, complete sequence	Bacillus subtilis	2488	2488	100%	0.0	99.93%	1550	NR_112116.2
Bacillus subtilis strain BCRC 10255 16S ribosomal RNA, partial sequence	Bacillus subtilis	2488	2488	100%	0.0	99.93%	1468	NR_116017.1
Bacillus tequilensis strain 10b 16S ribosomal RNA, partial sequence	Bacillus tequilensis	2488	2488	100%	0.0	99.93%	1456	NR_104919.1
Bacillus subtilis subsp. subtilis strain 168 16S ribosomal RNA, complete sequence	Bacillus subtilis subsp. subtilis	2483	2483	100%	0.0	99.85%	1550	NR_102783.2
Bacillus subtilis subsp. spizizenii strain NRRL B-23049 16S ribosomal RNA, partial sequence	Bacillus spizizenii	2483	2483	100%	0.0	99.85%	1409	NR_024931.1
Bacillus subtilis subsp. spizizenii strain NBRC 101239 16S ribosomal RNA, partial sequence	Bacillus spizizenii	2483	2483	100%	0.0	99.85%	1475	NR_112686.1
Bacillus halotolerans strain LMG 22477 16S ribosomal RNA, partial sequence	Bacillus halotolerans	2477	2477	100%	0.0	99.78%	1468	NR_115931.1
Bacillus halotolerans strain CECT 5687 16S ribosomal RNA, partial sequence	Bacillus halotolerans	2477	2477	100%	0.0	99.78%	1468	NR_115930.1
Bacillus halotolerans strain LMG 22476 16S ribosomal RNA, partial sequence	Bacillus halotolerans	2477	2477	100%	0.0	99.78%	1468	NR_115929.1
Bacillus mojavensis strain ifo 15718 16S ribosomal RNA, partial sequence	Bacillus mojavensis	2477	2477	100%	0.0	99.78%	1407	NR_118290.1
Bacillus halotolerans strain CR-95 16S ribosomal RNA, partial sequence	Bacillus halotolerans	2477	2477	100%	0.0	99.78%	1420	NR_115282.1
Bacillus mojavensis strain IFO15718 16S ribosomal RNA, partial sequence	Bacillus mojavensis	2477	2477	100%	0.0	99.78%	1526	NR_024693.1
Bacillus halotolerans strain DSM 8802 16S ribosomal RNA, partial sequence	Bacillus halotolerans	2477	2477	100%	0.0	99.78%	1545	NR_115063.1
Bacillus mojavensis strain NBRC 15718 16S ribosomal RNA, partial sequence	Bacillus mojavensis	2477	2477	100%	0.0	99.78%	1475	NR_112725.1
Bacillus nakamurai strain NRRL B-41091 16S ribosomal RNA, partial sequence	Bacillus nakamurai	2471	2471	100%	0.0	99.70%	1508	NR_151897.1
Bacillus vallismortis strain NBRC 101236 16S ribosomal RNA, partial sequence	Bacillus vallismortis	2468	2468	100%	0.0	99.63%	1475	N[illegible]
Bacillus vallismortis strain DSM 11031 16S ribosomal RNA, partial sequence	Bacillus vallismortis	2466	2466	100%	0.0	99.63%	1530	N[illegible]

b

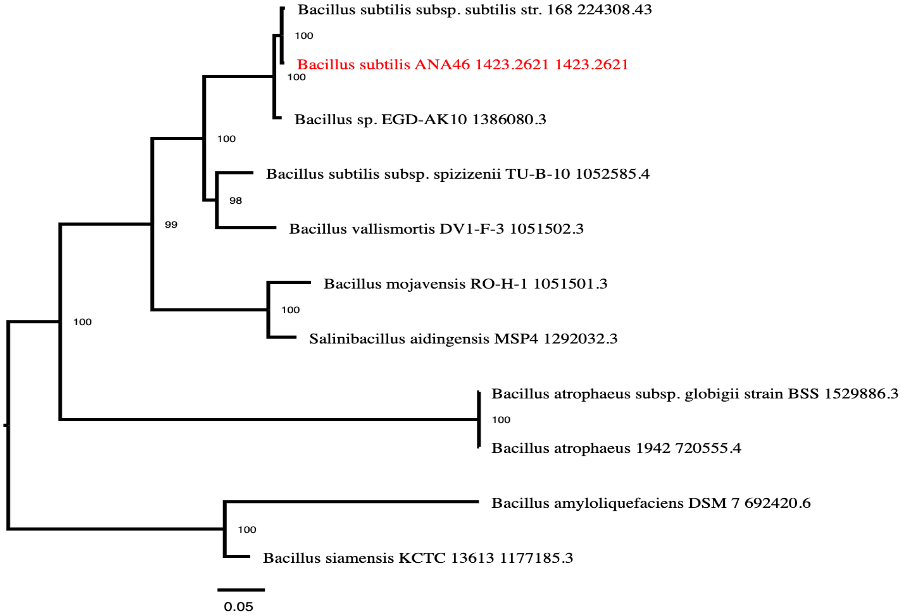

Supplementary Fig. 1 BLAST analysis (a) and Phylogenetic tree (b) of *B. subtilis* ANA46. (a) NCBI BLAST analysis of the 16S rRNA gene sequence of *B. subtilis* ANA46 showing 100% query coverage and ≥99.9% identity with top *B. subtilis* reference strains, confirming species-level identity. **(b)** Phylogenetic tree based on 16S rRNA sequences demonstrating that *B. subtilis* ANA46 (highlighted in red) clusters tightly with *B. subtilis* subsp. subtilis strain 168, supported by 100% bootstrap confidence, indicating high genetic similarity and confirming accurate taxonomic classification. Scale bar represents 0.05 nucleotide substitutions per site.

a

Descriptions | Graphic Summary | Alignments | Taxonomy

Sequences producing significant alignments Download Manage Columns Show 10

select all *10 sequences selected* GenBank Graphics Distance tree of results

Description	Max Score	Total Score	Query Cover	E value	Per. Ident	Accession
Bacillus clausii strain BRM043935 16S ribosomal RNA gene, partial sequence	2547	2547	100%	0.0	99.57%	MH305350.1
Bacillus clausii strain ENTPro, complete genome	2547	17759	100%	0.0	99.57%	CP012475.1
Bacillus clausii strain ANA38 16S ribosomal RNA gene, partial sequence	2542	2542	100%	0.0	99.50%	MT110681.1
Bacillus clausii strain ANA37 16S ribosomal RNA gene, partial sequence	2542	2542	100%	0.0	99.50%	MT110679.1
Bacillus clausii strain ANA36 16S ribosomal RNA gene, partial sequence	2542	2542	100%	0.0	99.50%	MT107136.1
Bacillus clausii strain ANA35 16S ribosomal RNA gene, partial sequence	2542	2542	100%	0.0	99.50%	MT107086.1
Bacillus clausii strain SL4-4 16S ribosomal RNA gene, partial sequence	2542	2542	100%	0.0	99.50%	MK312486.1
Bacillus rhizosphaerae strain WA12 16S ribosomal RNA gene, partial sequence	2542	2542	100%	0.0	99.50%	KT595230.1
Bacillus clausii strain E2 16S ribosomal RNA gene, partial sequence	2542	2542	100%	0.0	99.50%	EU117277.1
Bacillus clausii KSM-K16 DNA, complete genome	2542	17715	100%	0.0	99.50%	AP006627.1

b

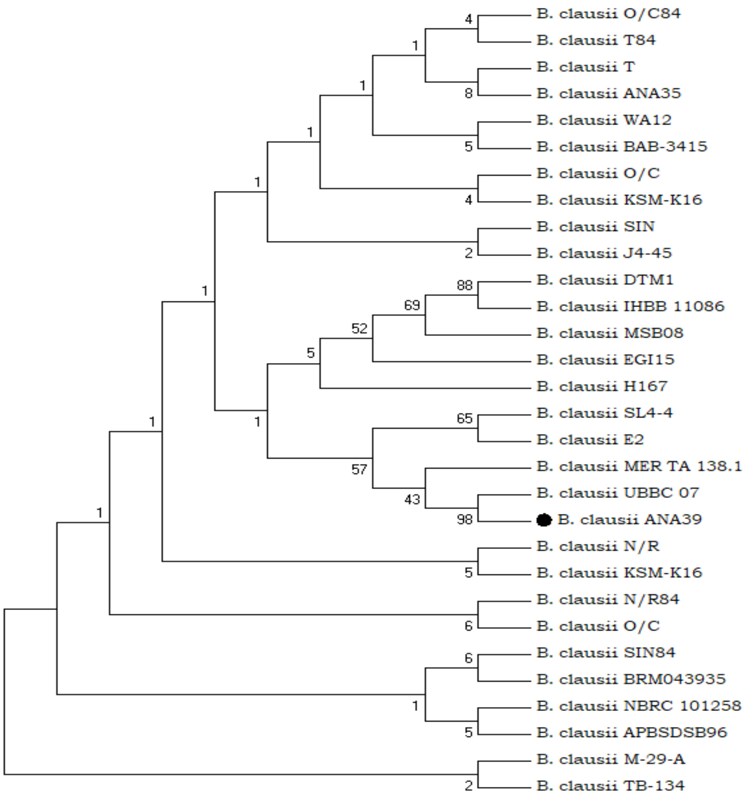

Supplementary Fig. 2 BLAST analysis (a) and Phylogenetic tree (b) of *B. clausii* ANA39. (a) NCBI BLAST results for the 16S rRNA gene sequence of *B. clausii* ANA39, showing 100% query coverage and ≥99.5% identity exclusively to top *B. clausii* reference strains, confirming species-level identity. **(b)** Phylogenetic tree based on 16S rRNA sequences of representative *B. clausii* strains. The strain ANA39 (●) clustered closely with *B. clausii* UBBC07 and MER TA138.1, supported by high bootstrap values (98%), confirming its taxonomic position within the *B. clausii* species.

a

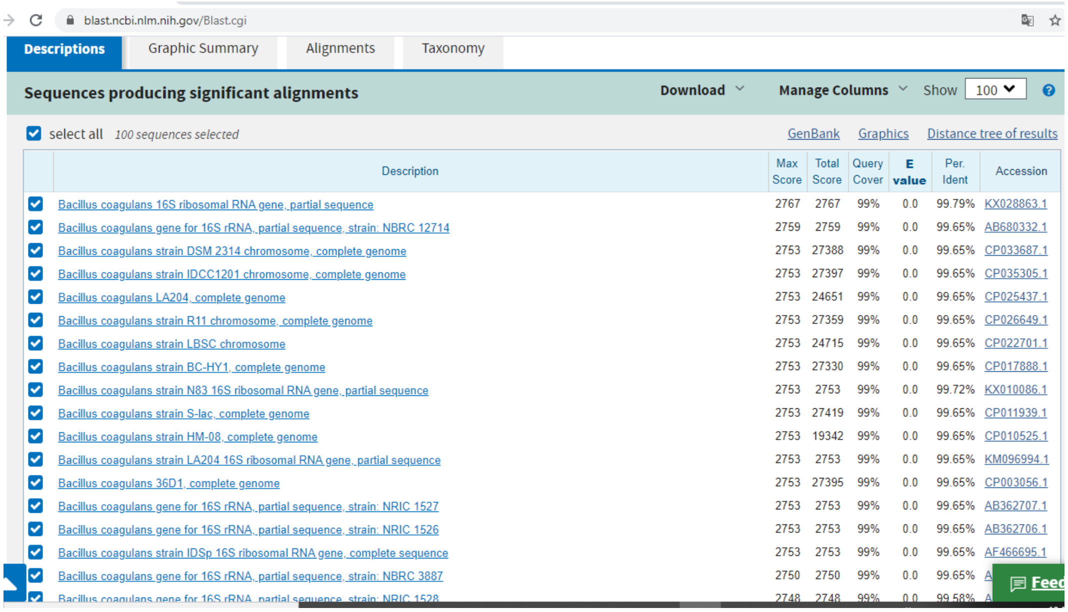

Description	Max Score	Total Score	Query Cover	E value	Per. Ident	Accession
Bacillus coagulans 16S ribosomal RNA gene, partial sequence	2767	2767	99%	0.0	99.79%	KX028863.1
Bacillus coagulans gene for 16S rRNA, partial sequence, strain: NBRC 12714	2759	2759	99%	0.0	99.65%	AB680332.1
Bacillus coagulans strain DSM 2314 chromosome, complete genome	2753	27388	99%	0.0	99.65%	CP033687.1
Bacillus coagulans strain IDCC1201 chromosome, complete genome	2753	27397	99%	0.0	99.65%	CP035305.1
Bacillus coagulans LA204, complete genome	2753	24651	99%	0.0	99.65%	CP025437.1
Bacillus coagulans strain R11 chromosome, complete genome	2753	27359	99%	0.0	99.65%	CP026649.1
Bacillus coagulans strain LBSC chromosome	2753	24715	99%	0.0	99.65%	CP022701.1
Bacillus coagulans strain BC-HY1, complete genome	2753	27330	99%	0.0	99.65%	CP017888.1
Bacillus coagulans strain N83 16S ribosomal RNA gene, partial sequence	2753	2753	99%	0.0	99.72%	KX010086.1
Bacillus coagulans strain S-lac, complete genome	2753	27419	99%	0.0	99.65%	CP011939.1
Bacillus coagulans strain HM-08, complete genome	2753	19342	99%	0.0	99.65%	CP010525.1
Bacillus coagulans strain LA204 16S ribosomal RNA gene, partial sequence	2753	2753	99%	0.0	99.65%	KM096994.1
Bacillus coagulans 36D1, complete genome	2753	27395	99%	0.0	99.65%	CP003056.1
Bacillus coagulans gene for 16S rRNA, partial sequence, strain: NRIC 1527	2753	2753	99%	0.0	99.65%	AB362707.1
Bacillus coagulans gene for 16S rRNA, partial sequence, strain: NRIC 1526	2753	2753	99%	0.0	99.65%	AB362706.1
Bacillus coagulans strain IDSp 16S ribosomal RNA gene, complete sequence	2753	2753	99%	0.0	99.65%	AF466695.1
Bacillus coagulans gene for 16S rRNA, partial sequence, strain: NBRC 3887	2750	2750	99%	0.0	99.65%	[illegible]
Bacillus coagulans gene for 16S rRNA, partial sequence, strain: NRIC 1528	2748	2748	99%	0.0	99.58%	[illegible]

b

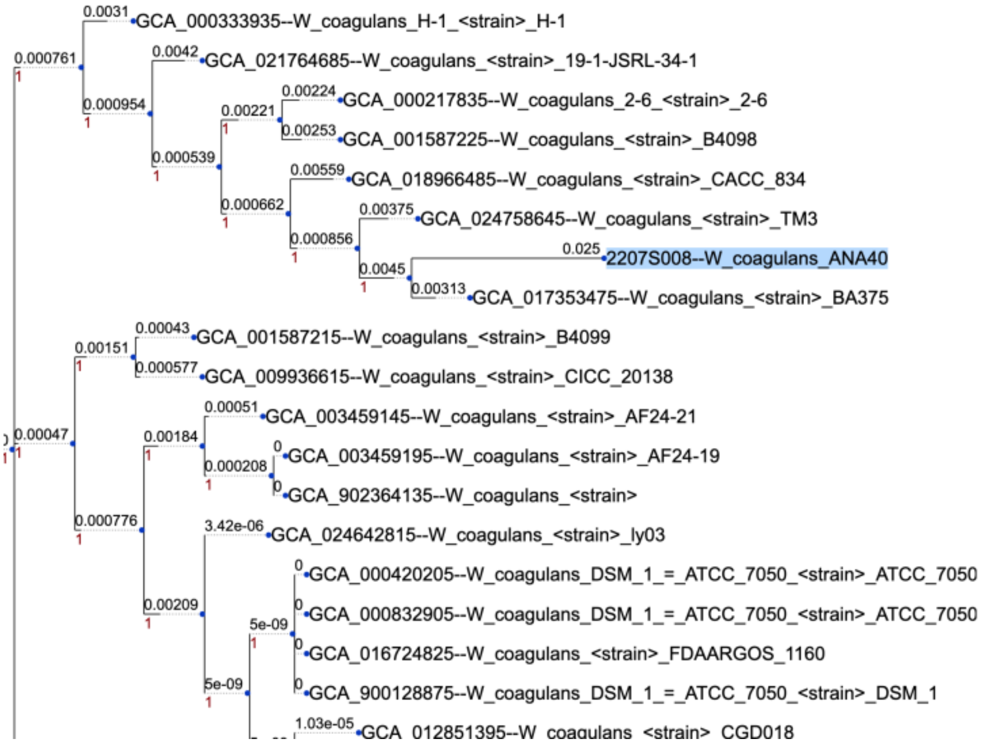

Supplementary Fig. 3 BLAST analysis (a) and Phylogenetic tree (b) of *B. coagulans* ANA40. **(a)** NCBI BLAST results for the 16S rRNA gene sequence of ANA40 showing top significant alignments exclusively to *B. coagulans* (≥99.8% identity; ≥99% query coverage across 16S rRNA and complete-genome entries). **(b)** Whole-genome phylogenetic tree of representative *B. coagulans* strains; ANA40 (highlighted in blue) clusters with TM3 and BA375, confirming species assignment. Scale bar denotes nucleotide substitutions per site.

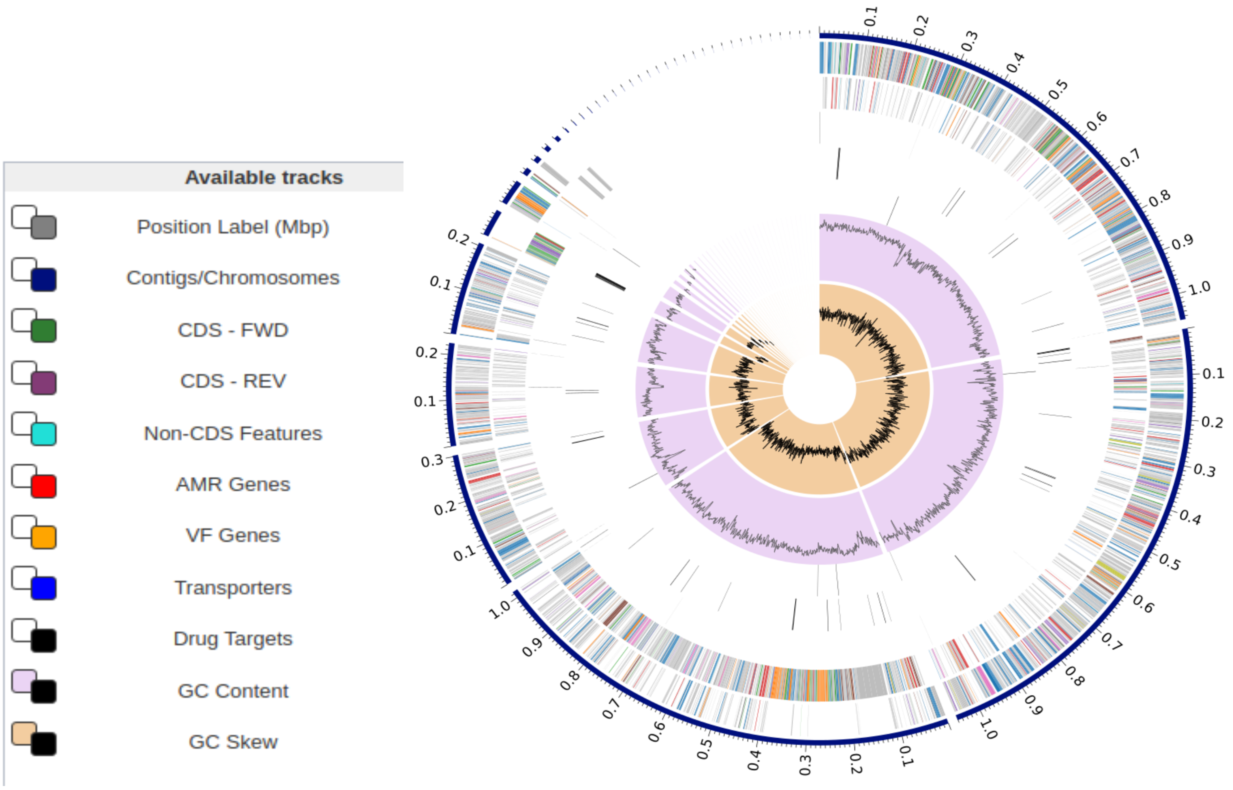

Supplemental Fig. 4 Whole genome circular mapping of *B. subtilis* ANA46. The outermost ring (blue) represents assembled contigs, followed by coding sequences (CDS) on the forward (green) and reverse (purple) strands, and non-coding features (cyan). Subsequent rings indicate antimicrobial resistance (AMR) genes (red), virulence factor (VF) genes (orange), transporters (light blue), and predicted drug targets (dark blue). The innermost rings display GC content (black) and GC skew (brown). The genome exhibits a balanced GC distribution, uniform gene density, and absence of notable AMR or virulence-associated genes, supporting the genetic safety and probiotic suitability of the *B. subtilis* ANA46 strain.

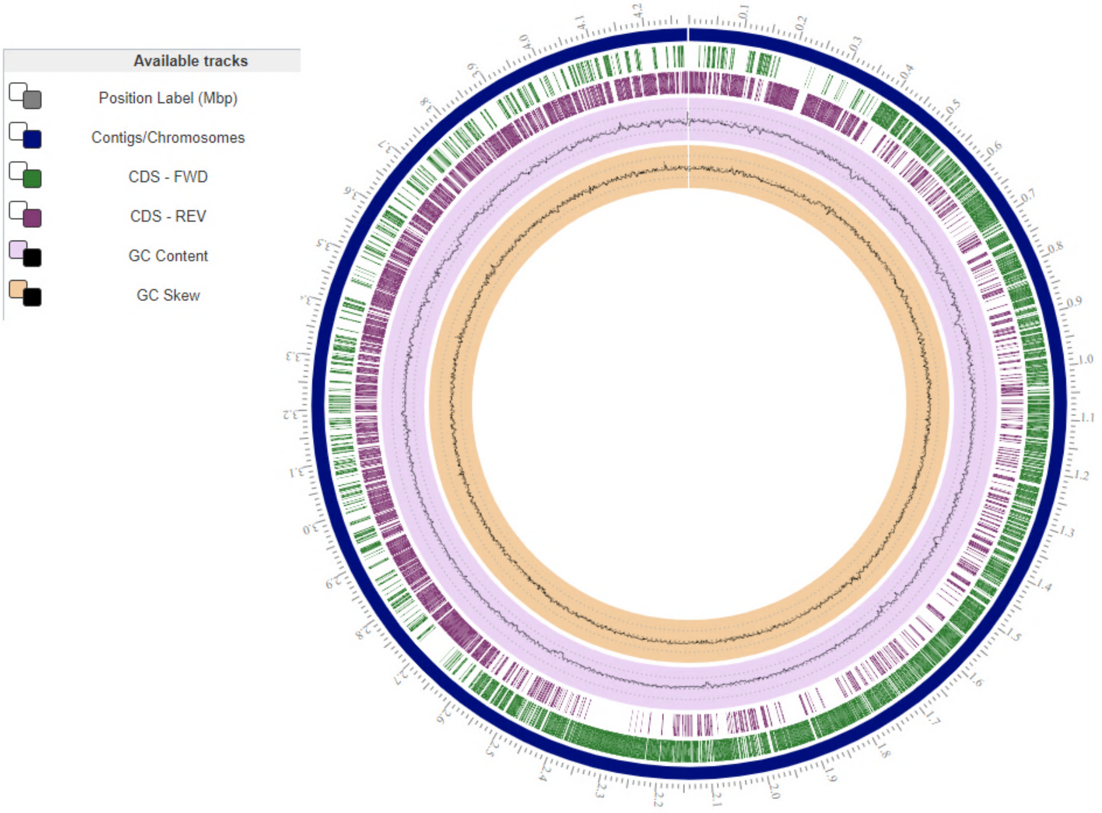

Supplementary Fig. 5 Whole genome circular mapping of *B. clausii* ANA39. The outermost ring (blue) represents assembled contigs, followed by coding sequences (CDS) on the forward (green) and reverse (purple) strands. The inner rings show GC content (black) and GC skew (brown). The map demonstrates a complete and well-organized genome architecture with balanced GC distribution and uniform gene density, confirming genomic stability and integrity of the *B. clausii* ANA39 strain used in this study.

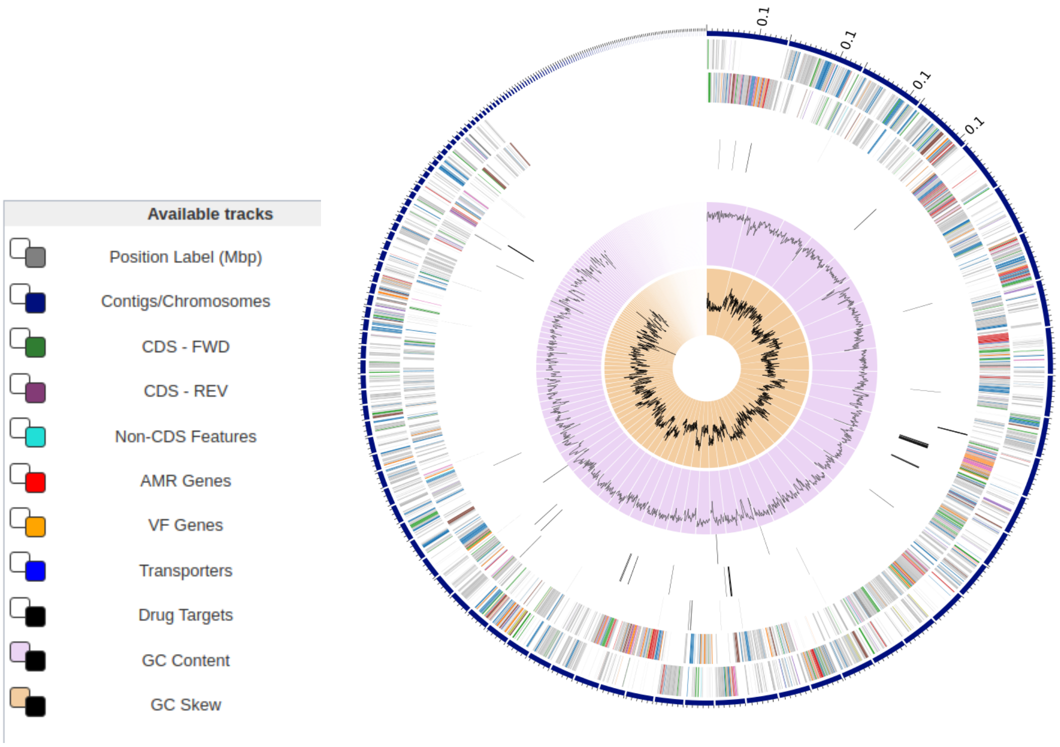

Supplementary Fig. 6 Whole genome circular mapping of *B. coagulans* ANA40. The outermost ring (blue) represents assembled contigs, followed by coding sequences (CDS) on the forward (green) and reverse (purple) strands, and non-coding features (cyan). Subsequent rings indicate antimicrobial resistance (AMR) genes (red), virulence factor (VF) genes (orange), transporters (light blue), and predicted drug targets (dark blue). The innermost rings display GC content (black) and GC skew (brown). The genome exhibits uniform gene density, balanced GC composition, and no significant AMR or virulence-associated genes, confirming the genomic stability and biosafety of the *B. coagulans* ANA40 strain

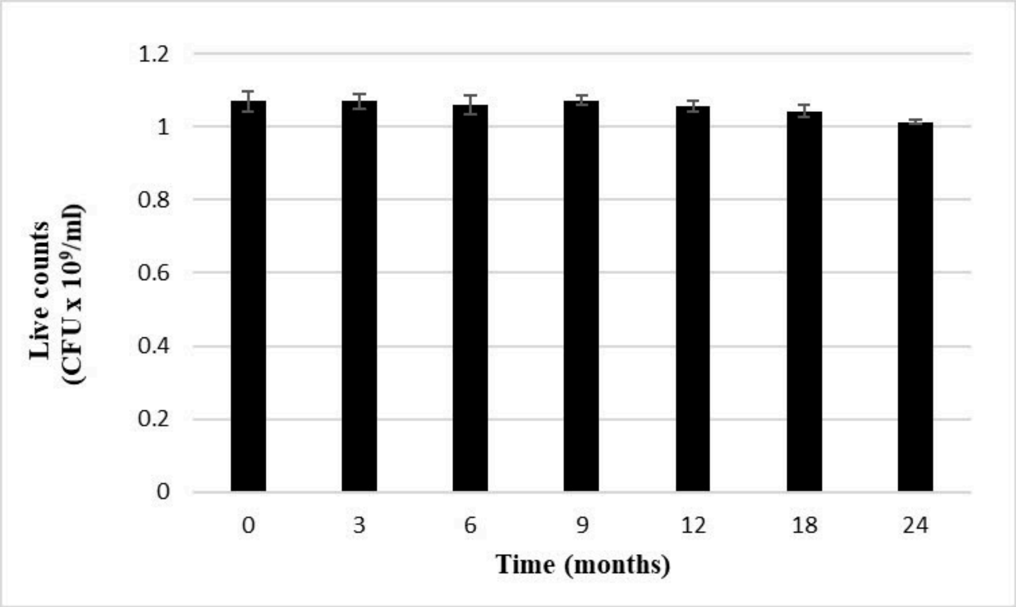

Supplementary Fig. 7 Time-dependent analysis of total viable *Bacillus* counts (*B. subtilis*, *B. clausii*, and *B. coagulans*) in LiveSpo X-SECRET during storage at room temperature. Data represent mean ± SD of three independent experiments, showing consistent viability over time.

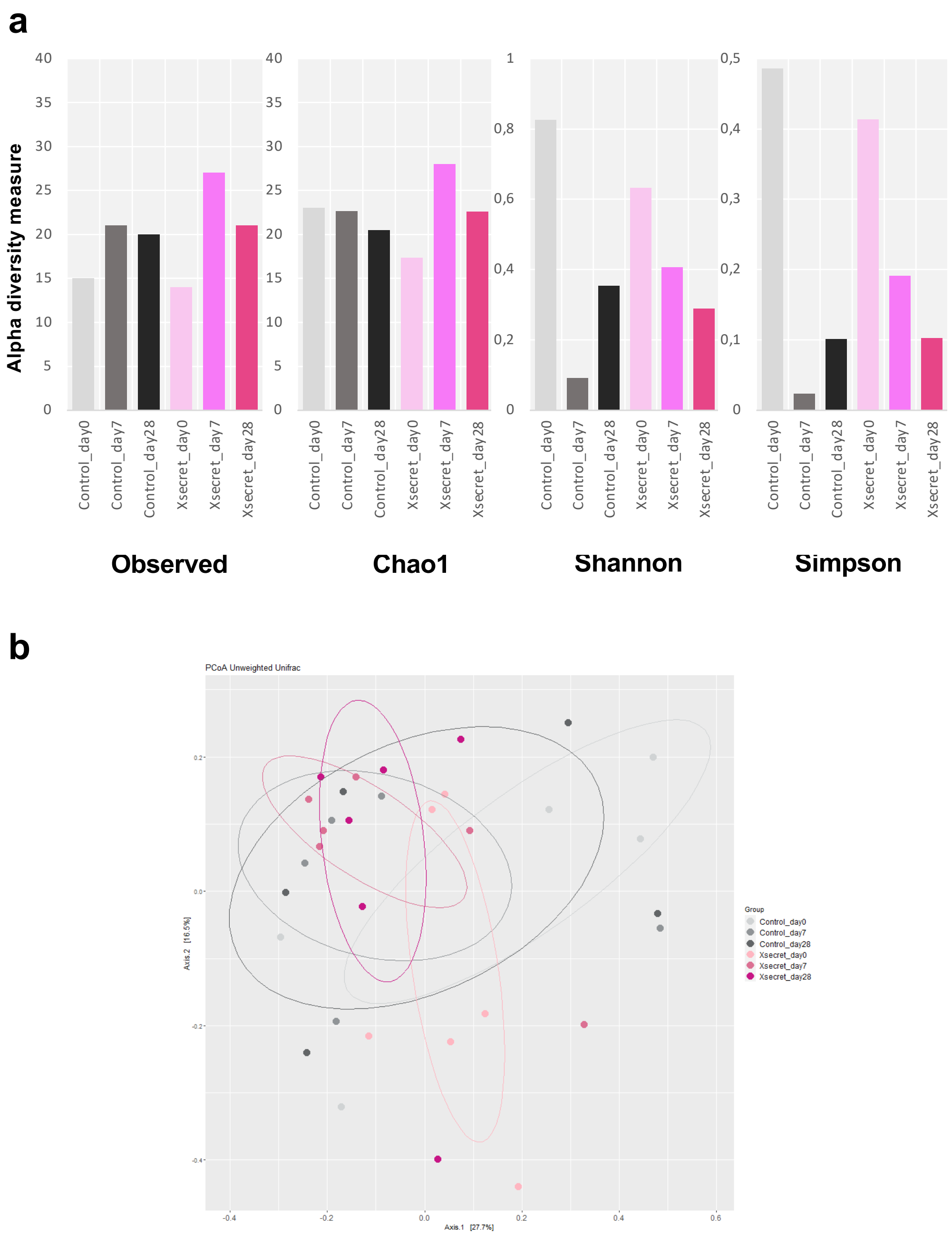

Supplementary Fig. 8 Analysis of alpha (a) and beta (b) diversity of 16S rRNA metagenome of vaginal samples between Control and X-secret groups at day 7, day 28 compared to day 0. (a) Alpha diversity indices including Observed species, Chao1, Shannon, and Simpson indices were used to evaluate within-sample diversity. Bars represent Control group at day 0 (grey), day 7 (light grey), and day 28 (black), and X-Secret group at day 0 (light pink), day 7 (pink), and day 28 (magenta). **(b)** Principal coordinate analysis (PCoA) based on unweighted UniFrac distances showing beta diversity between samples. The ellipses represent 95% confidence intervals of sample clustering.

3. CONSORT Checklist

Section/topic	No	CONSORT 2025 checklist item description	Reported on page no.
**Title and abstract**			
Title and structured abstract	1a	Identification as a randomised trial	1 (brief title)
	1b	Structured summary of the trial design, methods, results, and conclusions	2
**Open science**			
Trial registration	2	Name of trial registry, identifying number (with URL) and date of registration	1,2,7
Protocol and statistical analysis plan	3	Where the trial protocol and statistical analysis plan can be accessed	30
Data sharing	4	Where and how the individual de-identified participant data (including data dictionary), statistical code and any other materials can be accessed	30
Funding and conflicts of interest	5a	Sources of funding and other support (eg, supply of drugs), and role of funders in the design, conduct, analysis and reporting of the trial	35
	5b	Financial and other conflicts of interest of the manuscript authors	36
**Introduction**			
Background and rationale	6	Scientific background and rationale	3-6
Objectives	7	Specific objectives related to benefits and harms	6
**Methods**			
Patient and public involvement	8	Details of patient or public involvement in the design, conduct and reporting of the trial	8-10, Fig.1
Trial design	9	Description of trial design including type of trial (eg, parallel group, crossover), allocation ratio, and framework (eg, superiority, equivalence, non-inferiority, exploratory)	8-9
Changes to trial protocol	10	Important changes to the trial after it commenced including any outcomes or analyses that were not prespecified, with reason	Not applicable (No changes to

			trial methods or eligibility criteria after trial commencement)
Trial setting	11	Settings (eg, community, hospital) and locations (eg, countries, sites) where the trial was conducted	6,8
Eligibility criteria	12a	Eligibility criteria for participants	8
	12b	If applicable, eligibility criteria for sites and for individuals delivering the interventions (eg, surgeons, physiotherapists)	Not applicable
Intervention and comparator	13	Intervention and comparator with sufficient details to allow replication. If relevant, where additional materials describing the intervention and comparator (eg, intervention manual) can be accessed	9-13
Outcomes	14	Prespecified primary and secondary outcomes, including the specific measurement variable (eg, systolic blood pressure), analysis metric (eg, change from baseline, final value, time to event), method of aggregation (eg, median, proportion), and time point for each outcome	10-14
Harms	15	How harms were defined and assessed (eg, systematically, non-systematically)	Not applicable
Sample size	16a	How sample size was determined, including all assumptions supporting the sample size calculation	9
	16b	Explanation of any interim analyses and stopping guidelines	Not applicable (No interim analyses or stopping guidelines were pre-specified)
Randomisation:			
Sequence generation	17a	Who generated the random allocation sequence and the method used	9

	17b	Type of randomisation and details of any restriction (eg, stratification, blocking and block size)	9
Allocation concealment mechanism	18	Mechanism used to implement the random allocation sequence (eg, central computer/telephone; sequentially numbered, opaque, sealed containers), describing any steps to conceal the sequence until interventions were assigned	8,9
Implementation	19	Whether the personnel who enrolled and those who assigned participants to the interventions had access to the random allocation sequence	9
Blinding	20a	Who was blinded after assignment to interventions (eg, participants, care providers, outcome assessors, data analysts)	9
	20b	If blinded, how blinding was achieved and description of the similarity of interventions	9
Statistical methods	21a	Statistical methods used to compare groups for primary and secondary outcomes, including harms	12-14
	21b	Definition of who is included in each analysis (eg, all randomised participants), and in which group	11-14
	21c	How missing data were handled in the analysis	Not applicable (missing data was described in the Statistical Analysis Plan)
	21d	Methods for any additional analyses (eg, subgroup and sensitivity analyses), distinguishing prespecified from post hoc	Not applicable (No subgroup or adjusted analyses were planned or conducted)
**Results**			

Participant flow, including flow diagram	22a	For each group, the numbers of participants who were randomly assigned, received intended intervention, and were analysed for the primary outcome	14-15, Fig,1
	22b	For each group, losses and exclusions after randomisation, together with reasons	14-15, Fig.1
Recruitment	23a	Dates defining the periods of recruitment and follow-up for outcomes of benefits and harms	14
	23b	If relevant, why the trial ended or was stopped	Fig.1
Intervention and comparator delivery	24a	Intervention and comparator as they were actually administered (eg, where appropriate, who delivered the intervention/comparator, how participants adhered, whether they were delivered as intended (fidelity))	14-24
	24b	Concomitant care received during the trial for each group	14,15
Baseline data	25	A table showing baseline demographic and clinical characteristics for each group	Table 1 (39-40)
Numbers analysed, outcomes and estimation	26	For each primary and secondary outcome, by group:<br>● the number of participants included in the analysis<br>● the number of participants with available data at the outcome time point<br>● result for each group, and the estimated effect size and its precision (such as 95% confidence interval)<br>● for binary outcomes, presentation of both absolute and relative effect size	Fig. 1-6 (37-38)
Harms	27	All harms or unintended events in each group	15,16
Ancillary analyses	28	Any other analyses performed, including subgroup and sensitivity analyses, distinguishing pre-specified from post hoc	Not-applicable (No subgroup or sensitivity analyses were performed beyond the predefined outcomes)
**Discussion**			

Interpretation	29	Interpretation consistent with results, balancing benefits and harms, and considering other relevant evidence	24-29
Limitations	30	Trial limitations, addressing sources of potential bias, imprecision, generalisability, and, if relevant, multiplicity of analyses	28, 29

Citation: Hopewell S, Chan AW, Collins GS, Hróbjartsson A, Moher D, Schulz KF, et al. CONSORT 2025 Statement: updated guideline for reporting randomised trials. BMJ. 2025; 388:e081123. https://dx.doi.org/10.1136/bmj-2024-081123
© 2025 Hopewell et al. This is an Open Access article distributed under the terms of the Creative Commons Attribution License (https://creativecommons.org/licenses/by/4.0/), which permits unrestricted use, distribution, and reproduction in any medium, provided the original work is properly cited.

*We strongly recommend reading this statement in conjunction with the CONSORT 2025 Explanation and Elaboration and/or the CONSORT 2025 Expanded Checklist for important clarifications on all the items. We also recommend reading relevant CONSORT extensions. See www.consort-spirit.org.
