# Supplementary material for: Vaginal-spray Bacillus spore probiotics as a potential treatment and reducing recurrence of bacterial vaginosis: randomized, double-blind, and controlled pilot study
Source: Commun Med (Lond). 2025 Nov 18;5:527. doi: 10.1038/s43856-025-01236-4 (PMC12717237; doi:10.1038/s43856-025-01236-4)
Supplement: Supplementary file 2 — Description of Additional Supplementary Files [file 43856_2025_1236_MOESM2_ESM.docx]

**Description of Additional Supplementary Files**

File name: Supplementary Data 1

Description: Rabbit sub-acute toxicity report for LiveSpo X-Secret.

File name: Supplementary Data 2

Description: Vaginal mucosal irritation report for LiveSpo X-Secret.

File name: Supplementary Data 3

Description: Source Data for all main tables and figures
