# Supplementary material for: Vaginal-spray Bacillus spore probiotics as a potential treatment and reducing recurrence of bacterial vaginosis: randomized, double-blind, and controlled pilot study
Source: Commun Med (Lond). 2025 Nov 18;5:527. doi: 10.1038/s43856-025-01236-4 (PMC12717237; doi:10.1038/s43856-025-01236-4)
Supplement: Supplementary file 3 — Supplementary data 1 [file 43856_2025_1236_MOESM3_ESM.pdf]

NATIONAL INSTITUTE OF DRUG QUALITY CONTROL

LABORATORY OF PHARMACOLOGY

TEST RESULTS

SUB-ACUTE TOXICITY

(Test results are valid for the test samples only)

GENERAL INFORMATION

Sample name:	LiveSpo® X-SECRET
Manufacturer:	LiveSpo Pharma Co., Ltd
Batch No.:	H623X
Manufacturing date:	17/06/2024
Expiration date:	16/06/2026
Sample sender:	LiveSpo Pharma Co., Ltd
Test criteria:	Sub-acute toxicity
Test documents:	1. Methods for determining the toxicity of the drug - Medical Publisher, 2014<br>2. Guidelines for preclinical testing of traditional and herbal medicines (issued under Decision No. 141/QĐ-K2ĐT dated 27/10/2015 by the Ministry of Health)<br>3. Standard procedure for sub-acute toxicity testing (VKN/TQKT-DL/04)
Formulations on the label:	*B. subtilis, B. clausii,* and *B. coagulans,* concentration ≥ 1 billion CFU/ml for LiveSpo® X-SECRET probiotic products
Sample description:	Light brown-yellow solution in 360° rotating bottles, labeled with product name, volume, manufacturer, manufacturing and expiry dates, and usage instructions.
Address:	Tam Hiep, Thanh Tri, Hanoi
Headquarter:	48 Hai Ba Trung, Hanoi

Translated by Nguyen Thi Van Anh, corresponding author

TEST RESULTS

Implementation period: From 21/08/2024 to 20/09/2024

1. Experimental animals

- Species and Breed: Mature, healthy New Zealand female rabbits that are non-pregnant, non-lactating, and have not undergone any prior experiments, with a body weight of approximately 1.8–2.2 kg.
- Quantity: 21 rabbits randomly divided into 3 experimental groups (1 control group and 2 sample-treated groups), with 7 rabbits in each group.
- Source: Breeding Department - Laboratory of Pharmacology – National Institute of Drug Quality Control
- Care conditions: Each rabbit is housed individually in a controlled environment with appropriate temperature and humidity, and provided with food and water as needed. All procedures involving the experimental animals comply with the animal care and use guidelines established by the Laboratory of Pharmacology - National Institute for Drug Quality Control.

2. Procedure

2.1. Preparation of test samples

Selection of Experimental dosages: Based on the maximum vaginal spray dosage intended for human use of 9 sprays (0.72 ml of test sample suspension) per person per day (equivalent to 7.2 x 10^8 probiotic spores per person per day) and using a conversion factor of 3.1 between rabbits and humans, two experimental vaginal spray dosages for rabbits were chosen:

- Clinical dose: Equivalent to the intended human dosage: 0.558 sprays (0.045 ml of test sample suspension per kg of rabbit per day, corresponding to 4.45 x 10^7 probiotic spores per kg of rabbit per day).
- High dose: 5 folds of the intended human dosage: 2.790 sprays (0.223 ml of test sample suspension per kg of rabbit per day, corresponding to 2.23 x 10^8 probiotic spores per kg of rabbit per day).

Handling and preparation of test samples:

- Control sample: Physiological saline (0.9% NaCl).

- Test sample: Used as is, without modifications.

2.2. Experiment

The experiment was conducted on 21 rabbits, divided into three groups of 7 rabbits each. The experimental arrangement and dosage levels are summarized in Table 1.

Table 1. Sub-acute experimental dosage levels on rabbits

Group	Sample administered	Number of vaginal sprays per rabbit (*)	Dosage (ml of test sample/kg of rabbit)
Control group	07	2.800 sprays/kg of rabbit	---
Low-dose group	07	0.558 sprays/kg of rabbit/day	0.045 ml/kg of rabbit/day, corresponding to 4.45×10^7 probiotic spores/kg of rabbit/day
High-dose group	07	2.790 sprays/kg of rabbit/day	0.223 ml/kg of rabbit/day, corresponding to 2.23×10^8 probiotic spores/kg of rabbit/day.

(*) The number of sprays will be rounded up to the nearest whole number per rabbit. The maximum volume per spray, when the bottle is full, is 0.08 ml/spray; shake well before spraying.

2.3. Monitoring schedule and assessment

- Daily monitoring: Observe the rabbits daily for food and water consumption, general condition and activity, stool and urine condition, and any abnormal signs (if present).
- Weight measurement: Record the rabbits' weight at 0, 7, 14, 21, and 28 days during the sample spraying period.
- Hematological and biochemical tests: Measure hematological indicators related to blood-forming function (red blood cell count, white blood cell count, platelet count, hemoglobin, hematocrit), liver function indicators (AST, ALT, total bilirubin, cholesterol, albumin), kidney function indicators (creatinine, urea), and glucose levels at 0, 14, and 28 days, during sample spraying. Compare results between the test and control groups using statistical methods.

- Post-experiment analysis: After the experiment, euthanize the rabbits for macroscopic observation of the heart, liver, kidneys, lungs, stomach, intestines, and vagina.
- Histopathological examination: Randomly select 3 rabbits per group and prepare histopathological slides of the liver, kidneys, and vagina for microscopic evaluation of these tissues immediately after stopping the sample spraying.

2.4. Statistical analysis and data presentation

Experimental data are presented as the mean ± standard deviation (mean ± SD) and analyzed statistically using the Student's t-test to compare differences in the same parameter between the control and test groups.

3. Results

3.1. Rabbit condition

During the experimental period, all rabbits remained active, ate and drank well, had bright eyes, smooth fur, and dry feces. No abnormalities were observed in their physical condition, diet, or movement.

Table 2. Results of mice weight monitoring

**Group (*n* = 7)**	**Rabbit weight** *(kg)*					***P****
	Before Experiment (m_0)	After 7 days (m_1)	After 14 days (m_2)	After 21 days (m_3)	After 28 days (m_4)	
**Control (C)**	2.00 ± 0.12	2.09 ± 0.12	2.17 ± 0.16	2.28 ± 0.16	2.39 ± 0.18	$P_{b-a} < 0.001$
% compared to before experiment		104.6 %	108.5 %	114.1 %	119.4 %	
**Test 1 (T1)**	2.05 ± 0.07	2.20 ± 0.13	2.24 ± 0.16	2.35 ± 0.15	2.43 ± 0.15	$P_{b-a} < 0.001$<br>$P_{b(T1-C)} > 0.05$<br>$P_{a(T1-C)} > 0.05$
% compared to before experiment		107.6 %	109.1 %	114.5 %	118.4 %	
**Test 2 (T2)**	2.02 ± 0.09	2.17 ± 0.07	2.22 ± 0.10	2.34 ± 0.14	2.45 ± 0.11	$P_{b-a} < 0.001$

% compared to before experiment		107.4 %	109.7 %	115.8 %	121.1 %	$P_{b(T2-C)} > 0.05$<br>$P_{a(T2-C)} > 0.05$

**Two-sided Student's t-test with an adjusted significance level of 0.05/2 for two test groups and of 0.05/4 for four time points in the same group.*

Monitoring of rabbit weight during the experiment showed:

- Before the experiment (before spraying the test sample): The average weight of rabbits in the test groups before being subjected to the experiment showed no significant difference compared to the control group ($P_{\text{before (T1-C)}} > 0.05$; $P_{\text{before (T2-C)}} > 0.05$).
- After 28 days of spraying the test sample: Rabbits in the control group and the two test groups gained weight at each evaluation time point. There was a significant difference in the weight of the rabbits when comparing the results after 28 days of testing with before testing within each group ($P_{\text{before-after}} < 0.05$). No significant difference in average weight was observed between the test groups and the control group ($P_{\text{after (T1-C)}} > 0.05$; $P_{\text{after (T2-C)}} > 0.05$).

3.2. Results of monitoring hematological indicators related to hematopoietic function

a. Before the experiment (before spraying the test sample)

Table 3. Hematological indicators before the experiment

**Parameter**	**Control (*n* = 7)**	**T1 group (*n* = 7)**	***P* *(T1-C)***	**T2 group (*n* = 7)**	***P* *(T2-C)***
**Red blood cells** *(×10^12/l)*	5.7 ± 0.3	5.7 ± 0.2	> 0.05	5.8 ± 0.7	> 0.05
**White blood cells** *(× 10^9/l)*	8.9 ± 2.8	8.1 ± 1.5	> 0.05	8.4 ± 2.5	> 0.05
**Platelets** *(× 10^9/l)*	357.7 ± 91.7	366.1 ± 79.6	> 0.05	369.9 ± 55.7	> 0.05
**Hematocrit** *(%)*	38.3 ± 1.7	37.8 ± 1.8	> 0.05	37.6 ± 3.6	> 0.05
**Hemoglobin** *(g/dl)*	12.8 ± 0.7	12.7 ± 0.5	> 0.05	12.7 ± 1.1	> 0.05

**Two-sided Student's t-test with an adjusted significance level of 0.05/2 for two test groups.*

b. After 14 days of spraying the test sample

Table 4. Hematological indicators after 14 days of spraying the test sample

Indicators	Control (n = 7)	T1 group (n = 7)	*$P_{(T1-C)}$	T2 group (n = 7)	*$P_{(T2-C)}$
**Red blood cells** *(× 10^{12}/l)*	6.1 ± 0.9	6.4 ± 1.5	> 0.05	6.4 ± 1.0	> 0.05
**White blood cells** *(× 10^{9}/l)*	8.3 ± 2.3	8.0 ± 2.3	> 0.05	8.2 ± 2.1	> 0.05
**Platelets** *(× 10^{9}/l)*	358.6 ± 36.8	391.7 ± 61.8	> 0.05	413.4 ± 108.1	> 0.05
**Hematocrit** *(%)*	38.1 ± 3.0	39.5 ± 3.0	> 0.05	36.7 ± 2.5	> 0.05
**Hemoglobin** *(g/dl)*	12.0 ± 1.0	12.2 ± 1.3	> 0.05	11.7 ± 0.9	> 0.05

**Two-sided Student's t-test with an adjusted significance level of 0.05/2 for two test groups.*

c. After 14 days of spraying the test sample

Table 5. Hematological indicators after 28 days of spraying the test sample

Indicators	Control (n = 7)	T1 group (n = 7)	*$P_{(T1-C)}$	T2 group (n = 7)	*$P_{(T2-C)}$
**Red blood cells** *(× 10^{12}/l)*	5.5 ± 0.4	5.7 ± 0.2	> 0.05	5.6 ± 0.5	> 0.05
**White blood cells** *(× 10^{9}/l)*	7.9 ± 1.7	8.1 ± 2.1	> 0.05	6.5 ± 1.3	> 0.05
**Platelets** *(× 10^{9}/l)*	408.6 ± 79.5	357.1 ± 64.0	> 0.05	403.1 ± 76.1	> 0.05
**Hematocrit** *(%)*	38.3 ± 2.0	39.9 ± 1.1	> 0.05	37.9 ± 2.3	> 0.05
**Hemoglobin** *(g/dl)*	12.6 ± 0.8	13.1 ± 0.4	> 0.05	12.5 ± 1.1	> 0.05

**Two-sided Student's t-test with an adjusted significance level of 0.05/2 for two test groups.*

Comments:

The results of the hematological tests indicate that:

- Before spraying the sample: There was no significant difference in hematological

parameters between the control group and the two experimental groups ($P_{before\ TN\ (T-C)}$ > 0.05).

- After 14 days and 28 days of spraying the sample: There was no significant difference in hematological parameters between the control group and the two experimental groups ($P_{after\ 14\ days\ (T-C)} > 0.05$; $P_{after\ 28\ days\ (T-C)} > 0.05$).

3.3. Indicators related to liver function

a. Before the experiment (before spraying the sample)

Table 6. Indicators related to liver function before the experiment

Indicators	Control (*n* = 7)	Group T1 (*n* = 7)	*$P_{(T1-C)}$	Group T2 (*n* = 7)	*$P_{(T2-C)}$
**AST** *(U/l)*	29.7 ± 7.3	26.2 ± 7.5	> 0.05	30.8 ± 8.5	> 0.05
**ALT** *(U/l)*	68.7 ± 16.4	68.6 ± 15.3	> 0.05	67.0 ± 13.4	> 0.05
**Total Bilirubin** *(mmol/l)*	1.0 ± 0.3	0.8 ± 0.1	> 0.05	1.0 ± 0.3	> 0.05
**Albumin** *(g/l)*	39.1 ± 2.6	37.3 ± 1.9	> 0.05	41.1 ± 2.8	> 0.05
**Cholesterol** *(mmol/l)*	2.2 ± 0.3	2.7 ± 0.7	> 0.05	2.6 ± 0.6	> 0.05

**Two-sided Student's t-test with an adjusted significance level of 0.05/2 for two test groups.*

b. After 14 days of spraying the test sample

Table 7. Indicators related to liver function after 14 days of sample administration

Indicators	Control (*n* = 7)	Group T1 (*n* = 7)	$P_{(T1-C)}$	Group T2 (*n* = 7)	$P_{(T2-C)}$
**AST** *(U/l)*	33.3 ± 5.0	29.6 ± 5.8	> 0.05	30.9 ± 3.7	> 0.05
**ALT** *(U/l)*	65.3 ± 9.7	64.4 ± 13.8	> 0.05	64.1 ± 15.5	> 0.05
**Total Bilirubin** *(mmol/l)*	1.0 ± 0.3	0.9 ± 0.1	> 0.05	1.0 ± 0.3	> 0.05

**Albumin** *(g/l)*	38.0 ± 1.9	38.6 ± 2.7	> 0.05	39.1 ± 4.6	> 0.05
**Cholesterol** *(mmol/l)*	3.1 ± 0.5	2.9 ± 0.8	> 0.05	3.2 ± 0.8	> 0.05

**Two-sided Student's t-test with an adjusted significance level of 0.05/2 for two test groups.*

c. After 28 days of spraying the test sample

Table 8. Indicators related to liver function after 28 days of sample administration

Indicators	Control ($n = 7$)	Group T1 ($n = 7$)	$*P_{(T1-C)}$	Group T2 ($n = 7$)	$*P_{(T2-C)}$
**AST** *(U/l)*	34.0 ± 9.7	32.0 ± 9.9	> 0.05	28.5 ± 8.5	> 0.05
**ALT** *(U/l)*	61.7 ± 15.8	58.1 ± 9.9	> 0.05	56.0 ± 10.1	> 0.05
**Total Bilirubin** *(mmol/l)*	1.2 ± 0.2	1.2 ± 0.2	> 0.05	1.1 ± 0.3	> 0.05
**Albumin** *(g/dl)*	42.4 ± 2.1	42.1 ± 1.8	> 0.05	42.5 ± 2.3	> 0.05
**Cholesterol** *(mmol/l)*	1.8 ± 0.2	1.9 ± 0.4	> 0.05	2.2 ± 0.5	> 0.05

**Two-sided Student's t-test with an adjusted significance level of 0.05/2 for two test groups.*

Comments:

The results of testing some liver function indicators show:

- Before the sample administration: There were no significant differences in the liver function-related indicators between the control group and the two experimental groups ($P_{\text{before TN (T-C)}} > 0.05$).
- After 14 days of sample administration and after 28 days of sample administration: There were no significant differences in liver function-related indicators between the control group and the two experimental groups ($P_{\text{after 14 days (T-C)}} > 0.05$; $P_{\text{after 28 days (T-C)}} > 0.05$).

3.4. Indicators related to kidney function

a. Before the experiment (before spraying the test sample)

Table 9. Indicators related to kidney function before the experiment

Indicator	Control (n = 7)	Group T1 (n = 7)	*$P_{(T1-C)}$	Group T2 (n = 7)	*$P_{(T2-C)}$
**Ure** *(mmol/l)*	4.5 ± 0.5	4.7 ± 0.7	> 0.05	5.1 ± 0.8	> 0.05
**Creatinin** *(µmol/l)*	102.4 ± 14.2	103.9 ± 17.5	> 0.05	110.2 ± 15.1	> 0.05

**Two-sided Student's t-test with an adjusted significance level of 0.05/2 for two test groups.*

b. After 14 days of spraying the test sample

Table 10. Indicators related to kidney function after 14 days of sample administration

Indicator	Control (n = 7)	Group T1 (n = 7)	*$P_{(T1-C)}$	Group T2 (n = 7)	*$P_{(T2-C)}$
**Ure** *(mmol/l)*	5.5 ± 0.5	5.1 ± 1.0	> 0.05	5.4 ± 1.1	> 0.05
**Creatinin** *(µmol/l)*	108.0 ± 19.1	109.7 ± 14.3	> 0.05	111.9 ± 12.7	> 0.05

**Two-sided Student's t-test with an adjusted significance level of 0.05/2 for two test groups.*

c. After 28 days of spraying the test sample

Table 11. Indicators related to kidney function after 28 days of sample administration

Indicator	Control (n = 7)	Group T1 (n = 7)	*$P_{(T1-C)}$	Group T2 (n = 7)	*$P_{(T2-C)}$
**Ure** *(mmol/l)*	5.2 ± 0.8	5.0 ± 0.9	> 0.05	5.4 ± 1.2	> 0.05
**Creatinin** *(µmol/l)*	110.1 ± 17.1	110.3 ± 14.8	> 0.05	109.3 ± 15.8	> 0.05

**Two-sided Student's t-test with an adjusted significance level of 0.05/2 for two test groups.*

Comments:

The results of urea and creatinine tests indicate:

- Before spraying the test sample: There was no significant difference in kidney function parameters between the control group and the two test groups ($P_{before\ test\ (T-C)} > 0.05$).
- After 14 days of spraying the test sample and after 28 days of spraying the test

sample: There was no significant difference in kidney function parameters between the control group and the two test groups ($P_{after\ 14\ days\ (T-C)} > 0.05$; $P_{after\ 28\ days\ (T-C)} > 0.05$).

3.5. Blood glucose index monitoring results

a. Before the experiment (before spraying the test sample)

Table 12. Glucose index before the experiment

Indicator	Control (*n* = 7)	Group T1 (*n* = 7)	*$P_{(T1-C)}$	Group T2 (*n* = 7)	*$P_{(T2-C)}$
**Glucose** *(mmol/l)*	6.0 ± 1.0	5.8 ± 1.0	> 0.05	6.1 ± 0.6	> 0.05

**Two-sided Student's t-test with an adjusted significance level of 0.05/2 for two test groups and of 0.05/4 for four time points in the same group.*

b. After 14 days of spraying the test sample

Table 13. Glucose index after 14 days of spraying the test sample

Indicator	Control (*n* = 7)	Group T1 (*n* = 7)	*$P_{(T1-C)}$	Group T2 (*n* = 7)	*$P_{(T2-C)}$
**Glucose** *(mmol/l)*	5.6 ± 1.0	5.6 ± 0.8	> 0.05	5.5 ± 0.9	> 0.05

**Two-sided Student's t-test with an adjusted significance level of 0.05/2 for two test groups.*

c. After 28 days of spraying the test sample

Table 14. Glucose index after 28 days of spraying the test sample

Indicator	Control (*n* = 7)	Group T1 (*n* = 7)	*$P_{(T1-C)}$	Group T2 (*n* = 7)	*$P_{(T2-C)}$
**Glucose** *(mmol/l)*	5.4 ± 0.6	5.3 ± 0.5	> 0.05	5.1 ± 0.7	> 0.05

**Two-sided Student's t-test with an adjusted significance level of 0.05/2 for two test groups.*

Comments:

The glucose test results indicate:

- Before spraying the test sample: There was no significant difference in glucose levels between the control group and the two test groups ($P_{before\ test\ (T-C)} > 0.05$).
- After 14 days and 28 days of spraying the test sample: There was no significant difference in glucose levels between the control group and the two test groups ($P_{after\ 14\ days(T-C)} > 0.05$; $P_{after\ 28\ days(T-C)} > 0.05$).

3.6. Macroscopic observations

Macroscopic observations of the internal organs of all test rabbits revealed no abnormalities in the external appearance or coloration of the heart, liver, kidneys, lungs, stomach, intestines, or vagina in the test groups compared to the control group after the experiment.

Table 15. Macroscopic images of internal organs

**Macroscopic images**		
Control	***Test 1 (Low-dose)***	***Test 2 (high-dose)***
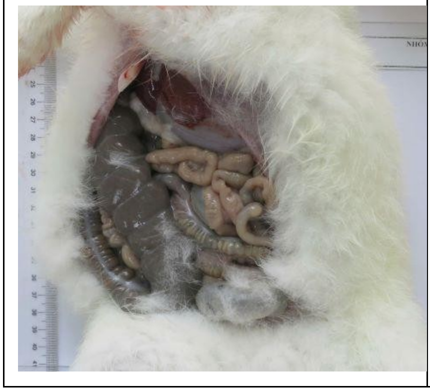	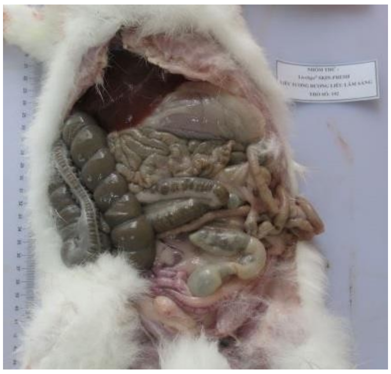	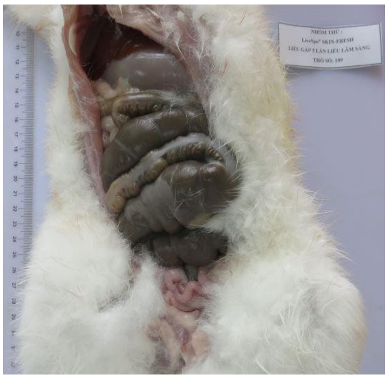

Table 16. Images of the heart, liver, spleen, kidneys, and vagina

**Images of the heart, liver, spleen, kidneys, and vagina**		
Control	***Test 1 (Low-dose)***	***Test 2 (high-dose)***
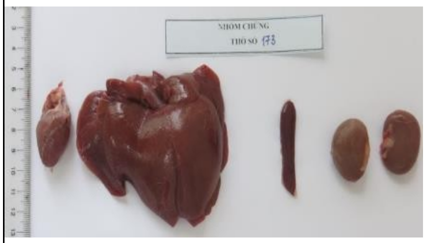	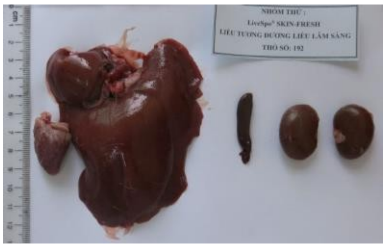	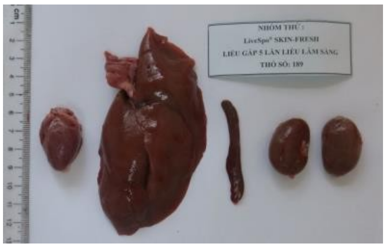
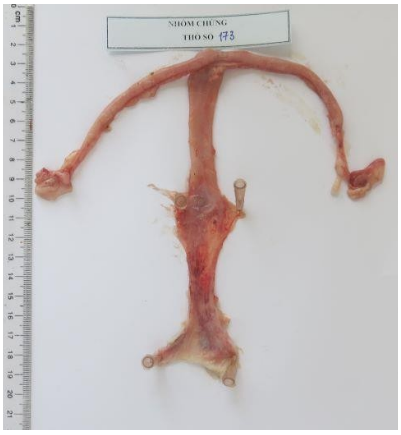	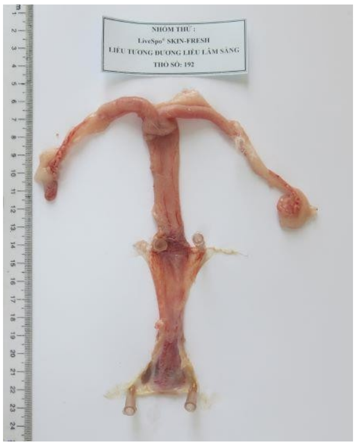	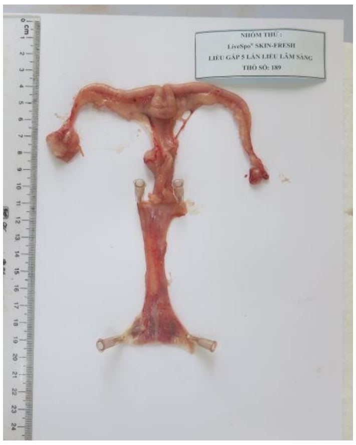

Translated by Nguyen Thi Van Anh, corresponding author

3.7. Microscopic observations

- Liver, kidney, and vaginal tissue samples were fixed in 10% formalin, stained with Hematoxylin and Eosin (HE) solution, and observed under an optical microscope.
- The microscopic observation results, conducted by the Department of Pathophysiology – Military Hospital 103, indicated that the liver, kidneys, and vagina of all test rabbits showed no damage, and the structural features were within normal limits. No abnormal symptoms related to the test sample were observed at either dose level compared to the control group.

The detailed microscopic analysis was as follows:

Liver:

- Hepatocytes: Normal.
- Sinusoids: Normal.
- Central vein: Normal.
- Portal area: Normal.
- Interstitial tissue: Normal.

Conclusion: Liver tissue morphology was normal, there was no apparent injury.

Kidneys:

- Glomeruli (capillaries, mesangium, Bowman's capsule): Normal.
- Renal tubules: Normal.
- Renal pelvis and calyces: Normal.
- Interstitial tissue: Normal.

Conclusion: Kidney tissue is normal and there was no apparent injury.

Vagina:

- Epithelium: Mild degeneration
- Leukocyte infiltration: Very mild
- Congested blood vessels: None
- Edema: None

Conclusion: Vaginal tissue is normal and there was no apparent injury.

Table 17. The histopathological anatomy images under microscope of liver, kidney, and vaginal (HE staining, 400x magnification)

No.	*Group*	*Liver tissue image*	*Kidney tissue image*	*Vaginal tissue image*
1	***Control***	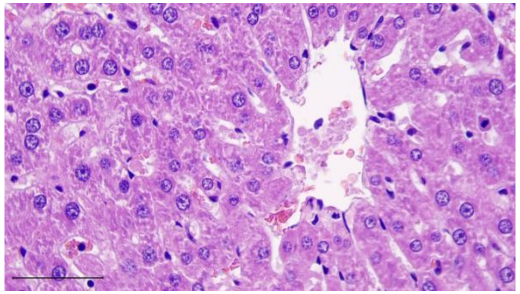 Normal and unscathed liver tissue morphology.	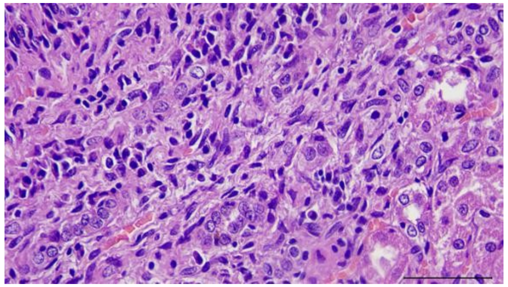 Normal and unscathed kidney tissue morphology.	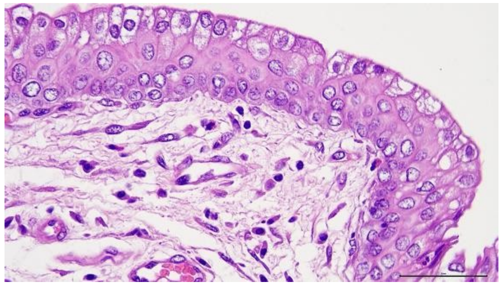 Normal and unscathed vaginal tissue morphology.
2	***Test 1 (low-dose)***	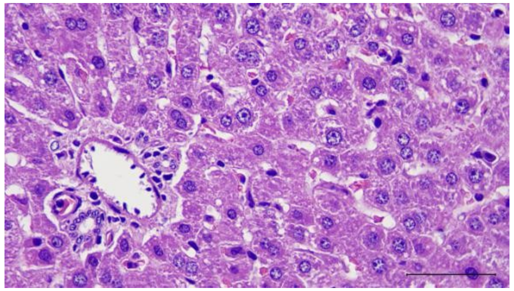 Normal and unscathed liver tissue morphology.	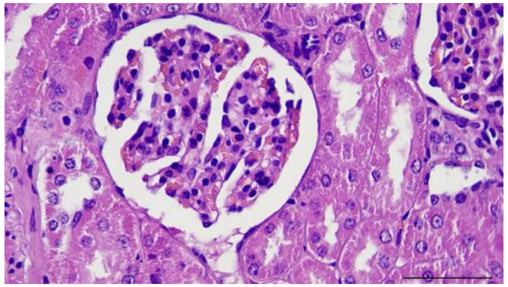 Normal and unscathed kidney tissue morphology.	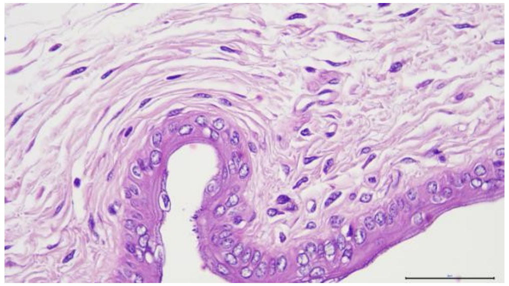 Normal and unscathed vaginal tissue morphology.

3	***Test 2 (high-dose)***	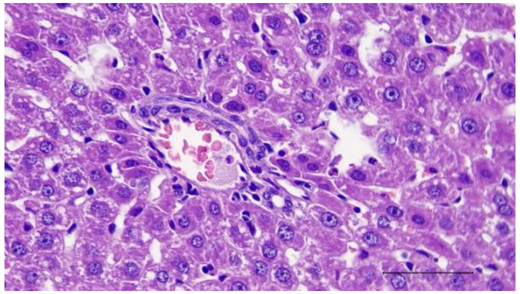<br>Normal and unscathed liver tissue morphology.	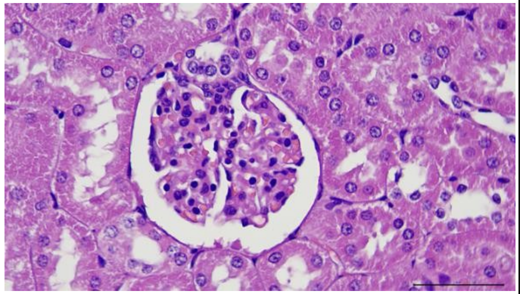<br>Normal and unscathed kidney tissue morphology.	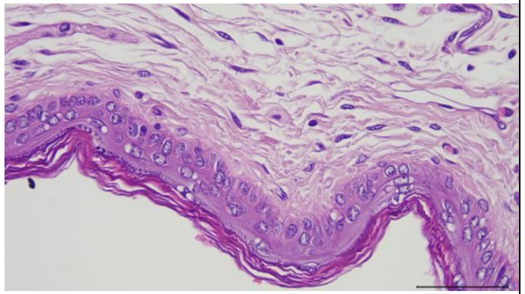<br>Normal and unscathed vaginal tissue morphology.

4. Conclusion

The results on sub-acute toxicity on rabbits of the sample LiveSpo® X-SECRET performed at National Institute of Drug Quality Control are as follows:

After continuously administering the test sample suspension to the rabbits for 28 days at two different dose levels 0,558 sprays/kg rabbit/day (corresponding to the maximum human dose of 9 sprays /person/day or equivalent to 7.2 x 10^8 CFU of *Bacillus subtilis*, *Bacillus clausii*, and *Bacillus coagulans*/person/day), 2.790 sprays /kg rabbit/day (5 folds of the intended human dose, equivalent to 3.6 x 10^9 CFU of *Bacillus subtilis*, *Bacillus clausii*, and *Bacillus coagulans*/person/day), the test sample had no effect on the body weight, physical condition, or activity of the test rabbits. The rabbits remained healthy and gained weight.

Regarding biochemical indicators for liver and kidney function (AST, ALT enzyme activity, albumin, total bilirubin, cholesterol, urea, creatinine), glucose, and hematological parameters (red blood cells, hemoglobin, hematocrit, white blood cells, platelets), there were no significant differences before the experiment, after 14 days, and after 28 days of spraying the test sample between the two test groups and the control group.

No abnormalities were observed in the heart, liver, kidneys, lungs, spleen, digestive system, or vagina of the test rabbits during macroscopic observation. Additionally, no histopathological damage was observed in the liver, kidneys, or vagina upon microscopic examination between the two test groups and the control group.

Hanoi, October 22, 2025

Certified by the Head of Organization (signed and stamped)	Laboratory of Pharmacology (Signed)
**Assoc. Prof. Doan Cao Son**	**MSc. Tran Thi Thanh Hue**

Translated by Nguyen Thi Van Anh, corresponding author

VIỆN KIỂM NGHIỆM THUỐC TRUNG ƯƠNG
KHOA DƯỢC LÝ

KẾT QUẢ THỬ NGHIỆM ĐỘC TÍNH BÁN TRƯỜNG DIỄN

(Kết quả thử nghiệm chỉ có giá trị với mẫu đem thử)

THÔNG TIN CHUNG

Tên mẫu:	LiveSpo® X-SECRET
Nhà sản xuất:	Công ty TNHH LiveSpo Pharma
Lô sản xuất:	H623X
Ngày sản xuất:	17/06/2024
Hạn dùng:	16/06/2026
Nơi gửi mẫu:	Công ty TNHH LiveSpo Pharma
Chỉ tiêu thử:	Độc tính bán trường diễn
Tài liệu thử:	1. Phương pháp xác định độc tính của thuốc – Nhà xuất bản Y học 2014<br>2. Hướng dẫn thử nghiệm tiền lâm sàng thuốc Đông Y, thuốc từ dược liệu (Ban hành kèm theo Quyết định số 141/QĐ-K2ĐT ngày 27/10/2015 của Bộ Y tế)<br>3. Thường quy kỹ thuật Thử nghiệm độc tính bán trường diễn của Viện Kiểm nghiệm thuốc Trung ương ***VKN/TQKT-DL/04.***
Công thức bào chế trên nhãn:	*B. subtilis, B. clausii* và *B. coagulans* nồng độ ≥ 1 tỷ CFU/ml cho sản phẩm probiotic LiveSpo® X-SECRET.
Tình trạng mẫu khi mở niêm phong để thử nghiệm:	Mẫu thử dạng hỗn dịch màu vàng nâu nhạt, được đóng trong bình xoay 360 độ có dán nhãn, trên nhãn ghi tên mẫu, thể tích, nơi sản xuất, ngày sản xuất, hạn dùng, hướng dẫn sử dụng.
Nơi thực hiện:	Khoa Dược lý - Viện Kiểm nghiệm thuốc Trung Ương
Địa chỉ	Tam Hiệp, Thanh Trì, Hà Nội
Trụ sở	48 Hai Bà Trưng, Hà Nội

KẾT QUẢ THỬ NGHIỆM

Thời gian thực hiện: Từ 21/08/2024 đến 20/09/2024

1. Động vật thí nghiệm

- Loài, giống: Thỏ cái Newzealand trưởng thành, khỏe mạnh, thỏ cái không mang thai hoặc cho con bú, chưa trải qua bất kỳ thử nghiệm nào trước đó, cân nặng khoảng 1,8 – 2,2 kg.

- Số lượng: 21 con được chia ngẫu nhiên thành 3 nhóm thử nghiệm (1 nhóm chứng và 2 nhóm dùng mẫu thử), mỗi nhóm 07 con.

- Nguồn gốc: Bộ phận chăn nuôi - Khoa Dược lý - Viện Kiểm nghiệm thuốc Trung Ương.

- Điều kiện chăm sóc: Thỏ được nuôi mỗi con một lồng trong phòng nuôi có kiểm soát nhiệt độ và độ ẩm thích hợp với thức ăn và nước uống theo nhu cầu. Tất cả các thao tác trên động vật thí nghiệm đều được tuân theo các quy trình về chăm sóc và sử dụng động vật thí nghiệm của Khoa Dược lý – Viện Kiểm nghiệm thuốc Trung Ương.

2. Tiến hành

2.1. Chuẩn bị mẫu thử

- Lựa chọn mức liều thử nghiệm: Dựa trên liều xịt âm đạo tối đa dự kiến dùng trên người là 9 nhát (0,72 ml hỗn dịch mẫu thử)/người/ngày (tương đương $\geq 7,2 \times 10^8$ bào tử lợi khuẩn/người/ngày), và sử dụng hệ số chuyển đổi liều giữa thỏ và người là 3,1 để lựa chọn 2 mức liều thử nghiệm xịt vào âm đạo thỏ là:

+ Liều lâm sàng tương ứng với mức liều dự kiến cho người: 0,558 nhát xịt (0,045 ml hỗn dịch mẫu thử/kg thỏ/ngày, tương đương với $\geq 4,45 \times 10^7$ bào tử lợi khuẩn/kg thỏ/ngày).

+ Liều cao gấp 5 lần liều dự kiến cho người: 2,790 nhát xịt (0,223 ml hỗn dịch mẫu thử/kg thỏ/ngày, tương đương với $\geq 2,23 \times 10^8$ bào tử lợi khuẩn/kg thỏ/ngày).

- Cách xử lý và chuẩn bị mẫu thử:

+ *Mẫu đối chứng*: Dung dịch nước muối sinh lý (NaCl 0,9 %)

+ *Mẫu thử:* Dùng nguyên mẫu

2.2. Bố trí thí nghiệm

Thí nghiệm được tiến hành trên 21 thỏ, chia thành 3 nhóm: mỗi nhóm 07 con. Bố trí thí nghiệm và thử với các mức liều theo Bảng 1.

Bảng 1. Các mức liều thử nghiệm bán trường diễn trên thỏ

Nhóm	Số thỏ thí nghiệm	Số nhát xịt âm đạo/thỏ (*)	Liều dùng (ml mẫu thử/kg thỏ)
Chứng	07	2,800 nhát/kg thỏ	---
Thử 1	07	0,558 nhát/kg thỏ/ngày	0,045 ml/kg thỏ/ngày, tương đương với ≥ 4,45 x 10^7 bào tử lợi khuẩn/kg thỏ/ngày
Thử 2	07	2,790 nhát/kg thỏ/ngày	0,223 ml/kg thỏ/ngày, tương đương với ≥ 2,23 x 10^8 bào tử lợi khuẩn/kg thỏ/ngày

(*) số nhát xịt sẽ được làm tròn lên số nguyên lớn hơn gần nhất tính trên mỗi thỏ

Thể tích tối đa của 1 nhát xịt khi đầy lọ là 0,08 ml/nhát xịt, lắc đều trước khi xịt.

2.3. Theo dõi và đánh giá

- Theo dõi thỏ hàng ngày về mức độ tiêu thụ thức ăn, nước uống, thể trạng và vận động, tình trạng phân, nước tiểu, các biểu hiện bất thường (nếu có) của thỏ.

- Xác định cân nặng của thỏ tại các thời điểm 0, 7, 14, 21, 28 ngày khi xịt mẫu thử.

- Xét nghiệm các chỉ số huyết học liên quan tới chức năng tạo máu (số lượng hồng cầu, bạch cầu, tiểu cầu, hemoglobin, hematocrit), các chỉ số liên quan tới chức năng gan (AST, ALT, bilirubin toàn phần, cholesterol, albumin), các chỉ số liên quan tới chức năng thận (creatinin, urê), chỉ số glucose tại các thời điểm 0, 14, 28 ngày khi xịt mẫu thử. So sánh kết quả của nhóm thử và nhóm chứng theo phương pháp thống kê.

- Sau thử nghiệm động vật được mổ để quan sát đại thể các tổ chức tim, gan, thận, phổi, dạ dày, ruột, âm đạo của tất cả các thỏ.

- Lấy ngẫu nhiên 03 thỏ/nhóm, tiến hành làm tiêu bản giải phẫu mô bệnh học gan, thận, âm đạo để đánh giá vi thể các tổ chức trên ngay sau khi dừng xịt mẫu thử.

2.4. Trình bày và xử lý số liệu

Số liệu thực nghiệm được trình bày dưới dạng giá trị trung bình cộng trừ độ lệch chuẩn (mean ± SD) và được xử lý thống kê bằng trắc nghiệm Student để so sánh sự khác nhau của cùng một chỉ số giữa nhóm chứng và nhóm thử.

3. Kết quả

3.1. Tình trạng thỏ

Trong thời gian thử nghiệm, tất cả các thỏ đều hoạt động bình thường, ăn uống tốt, mắt sáng, lông mượt, phân khô. Không có biểu hiện bất thường về thể trạng, ăn uống cũng như vận động.

Bảng 2. Kết quả theo dõi cân nặng của thỏ

Nhóm (n = 7)	**Cân nặng thỏ** *(kg)*					P
	TrướcTN (m_0)	*Sau 7 ngày (m_1)*	*Sau 14 ngày (m_2)*	*Sau 21 ngày(m_3)*	*Sau 28 ngày (m_4)*	
**Chứng (C)**	2,00 ± 0,12	2,09 ± 0,12	2,17 ± 0,16	2,28 ± 0,16	2,39 ± 0,18	$P_{trước-sau} < 0,001$
% so với trước thử nghiệm		104,6 %	108,5 %	114,1 %	119,4 %	
**Thử 1 (T1)**	2,05 ± 0,07	2,20 ± 0,13	2,24 ± 0,16	2,35 ± 0,15	2,43 ± 0,15	$P_{trước-sau} < 0,001$<br>$P_{trước(T1-C)} > 0,05$<br>$P_{sau(T1-C)} > 0,05$
% so với trước thử nghiệm		107,6 %	109,1 %	114,5 %	118,4 %	
**Thử 2 (T2)**	2,02 ± 0,09	2,17 ± 0,07	2,22 ± 0,10	2,34 ± 0,14	2,45 ± 0,11	$P_{trước-sau} < 0,001$<br>$P_{trước(T2-C)} > 0,05$<br>$P_{sau(T2-C)} > 0,05$
% so với trước thử nghiệm		107,4 %	109,7 %	115,8 %	121,1 %	

Theo dõi cân nặng thỏ trong quá trình thử nghiệm cho thấy:

- Trước thử nghiệm (trước khi xịt mẫu thử): Cân nặng trung bình của thỏ ở các nhóm thử trước khi đưa vào thử nghiệm không có sự khác biệt so với nhóm chứng ($P_{trước\ (T1-C)} > 0,05$; $P_{trước\ (T2-C)} > 0,05$).

- Sau 28 ngày xịt mẫu thử: Thỏ ở nhóm chứng và hai nhóm thử đều tăng cân ở mỗi thời điểm đánh giá. Có sự khác biệt có ý nghĩa về cân nặng của thỏ khi so sánh sau 28 ngày thử nghiệm với trước thử nghiệm trong mỗi nhóm ($P_{trước-\ sau} < 0,05$). Không có sự khác biệt có ý nghĩa về cân nặng trung bình giữa nhóm thử so với nhóm chứng ($P_{sau\ (T1-C)} > 0,05$; $P_{sau\ (T2-C)} > 0,05$).

3.2. Kết quả theo dõi các chỉ số huyết học liên quan tới chức năng tạo máu

a. Trước thử nghiệm (trước khi xịt mẫu thử)

Bảng 3. Các chỉ số huyết học trước khi dùng mẫu thử

Chỉ tiêu	Nhóm chứng (n = 7)	Nhóm T1 (n = 7)	$P_{(T1-C)}$	Nhóm T2 (n = 7)	$P_{(T2-C)}$
**Hồng cầu** ($\times 10^{12}/l$)	5,7 ± 0,3	5,7 ± 0,2	> 0,05	5,8 ± 0,7	> 0,05
**Bạch cầu** ($\times 10^{9}/l$)	8,9 ± 2,8	8,1 ± 1,5	> 0,05	8,4 ± 2,5	> 0,05
**Tiểu cầu** ($\times 10^{9}/l$)	357,7 ± 91,7	366,1 ± 79,6	> 0,05	369,9 ± 55,7	> 0,05
**Hematocrit** (%)	38,3 ± 1,7	37,8 ± 1,8	> 0,05	37,6 ± 3,6	> 0,05
**Hemoglobin** (g/dl)	12,8 ± 0,7	12,7 ± 0,5	> 0,05	12,7 ± 1,1	> 0,05

b. Thời điểm sau 14 ngày xịt mẫu thử

Bảng 4. Các chỉ số huyết học sau 14 ngày dùng mẫu thử

Chỉ tiêu	Nhóm chứng (n = 7)	Nhóm T1 (n = 7)	$P_{(T1-C)}$	Nhóm T2 (n = 7)	$P_{(T2-C)}$
**Hồng cầu** ($\times 10^{12}/l$)	6,1 ± 0,9	6,4 ± 1,5	> 0,05	6,4 ± 1,0	> 0,05
**Bạch cầu** ($\times 10^{9}/l$)	8,3 ± 2,3	8,0 ± 2,3	> 0,05	8,2 ± 2,1	> 0,05
**Tiểu cầu** ($\times 10^{9}/l$)	358,6 ± 36,8	391,7 ± 61,8	> 0,05	413,4 ± 108,1	> 0,05
**Hematocrit** (%)	38,1 ± 3,0	39,5 ± 3,0	> 0,05	36,7 ± 2,5	> 0,05
**Hemoglobin** (g/dl)	12,0 ± 1,0	12,2 ± 1,3	> 0,05	11,7 ± 0,9	> 0,05

c. Thời điểm sau 28 ngày xịt mẫu thử

Bảng 5. Các chỉ số huyết học sau 28 ngày dùng mẫu thử

Chỉ tiêu	Nhóm chứng (n = 7)	Nhóm T1 (n = 7)	$P_{(T1-C)}$	Nhóm T2 (n = 7)	$P_{(T2-C)}$
**Hồng cầu** *($\times 10^{12}/l$)*	5,5 ± 0,4	5,7 ± 0,2	> 0,05	5,6 ± 0,5	> 0,05
**Bạch cầu** *($\times 10^{9}/l$)*	7,9 ± 1,7	8,1 ± 2,1	> 0,05	6,5 ± 1,3	> 0,05
**Tiểu cầu** *($\times 10^{9}/l$)*	408,6 ± 79,5	357,1 ± 64,0	> 0,05	403,1 ± 76,1	> 0,05
**Hematocrit** *(%)*	38,3 ± 2,0	39,9 ± 1,1	> 0,05	37,9 ± 2,3	> 0,05
**Hemoglobin** *(g/dl)*	12,6 ± 0,8	13,1 ± 0,4	> 0,05	12,5 ± 1,1	> 0,05

Nhận xét

Kết quả xét nghiệm một số chỉ số huyết học cho thấy:

- Trước khi xịt mẫu thử: Không có sự khác biệt có ý nghĩa về chỉ số huyết học giữa nhóm chứng và 2 nhóm thử ($P_{trước\ TN\ (T-C)} > 0,05$).

- Sau 14 ngày xịt mẫu thử và sau 28 ngày xịt mẫu thử: Không có sự khác biệt có ý nghĩa về chỉ số huyết học giữa nhóm chứng và 2 nhóm thử ($P_{sau\ 14ngày\ (T-C)} > 0,05$; $P_{sau\ 28\ ngày\ (T-C)} > 0,05$).

3.3. Kết quả theo dõi các chỉ số liên quan tới chức năng gan

a. Trước thử nghiệm: (trước khi xịt mẫu thử)

Bảng 6. Các chỉ số liên quan chức năng gan trước khi dùng mẫu thử

Chỉ tiêu	Nhóm chứng (n = 7)	Nhóm T1 (n = 7)	$P_{(T1-C)}$	Nhóm T2 (n = 7)	$P_{(T2-C)}$
**AST** *(U/l)*	29,7 ± 7,3	26,2 ± 7,5	> 0,05	30,8 ± 8,5	> 0,05
**ALT** *(U/l)*	68,7 ± 16,4	68,6 ± 15,3	> 0,05	67,0 ± 13,4	> 0,05
**Bilirubin toàn phần** *(mmol/l)*	1,0 ± 0,3	0,8 ± 0,1	> 0,05	1,0 ± 0,3	> 0,05
**Albumin** *(g/l)*	39,1 ± 2,6	37,3 ± 1,9	> 0,05	41,1 ± 2,8	> 0,05
**Cholesterol** *(mmol/l)*	2,2 ± 0,3	2,7 ± 0,7	> 0,05	2,6 ± 0,6	> 0,05

b. Thời điểm sau 14 ngày xịt mẫu thử

Bảng 7. Các chỉ số liên quan chức năng gan sau 14 ngày dùng mẫu thử

Chỉ tiêu	Nhóm chứng (n = 7)	Nhóm T1 (n = 7)	$P_{(T1-C)}$	Nhóm T2 (n = 7)	$P_{(T2-C)}$
**AST** *(U/l)*	33,3 ± 5,0	29,6 ± 5,8	> 0,05	30,9 ± 3,7	> 0,05
**ALT** *(U/l)*	65,3 ± 9,7	64,4 ± 13,8	> 0,05	64,1 ± 15,5	> 0,05
**Bilirubin toàn phần** *(mmol/l)*	1,0 ± 0,3	0,9 ± 0,1	> 0,05	1,0 ± 0,3	> 0,05
**Albumin** *(g/l)*	38,0 ± 1,9	38,6 ± 2,7	> 0,05	39,1 ± 4,6	> 0,05
**Cholesterol** *(mmol/l)*	3,1 ± 0,5	2,9 ± 0,8	> 0,05	3,2 ± 0,8	> 0,05

c. Thời điểm sau 28 ngày xịt mẫu thử

Bảng 8. Các chỉ số liên quan chức năng gan sau 28 ngày dùng mẫu thử

Chỉ tiêu	Nhóm chứng (*n* = 7)	Nhóm T1 (*n* = 7)	$P_{(T1-C)}$	Nhóm T2 (*n* = 7)	$P_{(T2-C)}$
**AST** *(U/l)*	34,0 ± 9,7	32,0 ± 9,9	> 0,05	28,5 ± 8,5	> 0,05
**ALT** *(U/l)*	61,7 ± 15,8	58,1 ± 9,9	> 0,05	56,0 ± 10,1	> 0,05
**Bilirubin toàn phần** *(mmol/l)*	1,2 ± 0,2	1,2 ± 0,2	> 0,05	1,1 ± 0,3	> 0,05
**Albumin** *(g/dl)*	42,4 ± 2,1	42,1 ± 1,8	> 0,05	42,5 ± 2,3	> 0,05
**Cholesterol** *(mmol/l)*	1,8 ± 0,2	1,9 ± 0,4	> 0,05	2,2 ± 0,5	> 0,05

Nhận xét

Kết quả xét nghiệm một số chỉ số chức năng gan cho thấy:

- Trước khi xịt mẫu thử: Không có sự khác biệt có ý nghĩa về các chỉ số liên quan đến chức năng gan giữa nhóm chứng và 2 nhóm thử ($P_{trước\ TN\ (T-C)} > 0,05$).

- Sau 14 ngày xịt mẫu thử và sau 28 ngày xịt mẫu thử: Không có sự khác biệt có ý nghĩa về các chỉ số liên quan đến chức năng gan giữa nhóm chứng và 2 nhóm thử ($P_{sau\ 14\ ngày\ (T-C)} > 0,05$; $P_{sau\ 28\ ngày\ (T-C)} > 0,05$).

3.4. Kết quả theo dõi các chỉ số liên quan tới chức năng thận

a. Trước thử nghiệm: (trước khi xịt mẫu thử)

Bảng 9. Các chỉ số liên quan chức năng thận trước khi dùng mẫu thử

Chỉ tiêu	Nhóm chứng (*n* = 7)	Nhóm T1 (*n* = 7)	$P_{(T1-C)}$	Nhóm T2 (*n* = 7)	$P_{(T2-C)}$
**Urê** *(mmol/l)*	4,5 ± 0,5	4,7 ± 0,7	> 0,05	5,1 ± 0,8	> 0,05
**Creatinin** *(µmol/l)*	102,4 ± 14,2	103,9 ± 17,5	> 0,05	110,2 ± 15,1	> 0,05

b. Thời điểm sau 14 ngày xịt mẫu thử

Bảng 10. Các chỉ số liên quan chức năng thận sau 14 ngày dùng mẫu thử

Chỉ tiêu	Nhóm chứng (n = 7)	Nhóm T1 (n = 7)	$P_{(T1-C)}$	Nhóm T2 (n = 7)	$P_{(T2-C)}$
**Urê** *(mmol/l)*	5,5 ± 0,5	5,1 ± 1,0	> 0,05	5,4 ± 1,1	> 0,05
**Creatinin** *(µmol/l)*	108,0 ± 19,1	109,7 ± 14,3	> 0,05	111,9 ± 12,7	> 0,05

c. Thời điểm sau 28 ngày xịt mẫu thử

Bảng 11. Các chỉ số liên quan chức năng thận sau 28 ngày dùng mẫu thử

Chỉ tiêu	Nhóm chứng (n = 7)	Nhóm T1 (n = 7)	$P_{(T1-C)}$	Nhóm T2 (n = 7)	$P_{(T2-C)}$
**Urê** *(mmol/l)*	5,2 ± 0,8	5,0 ± 0,9	> 0,05	5,4 ± 1,2	> 0,05
**Creatinin** *(µmol/l)*	110,1 ± 17,1	110,3 ± 14,8	> 0,05	109,3 ± 15,8	> 0,05

Nhận xét

Kết quả xét nghiệm urê và creatinin cho thấy:

- Trước khi xịt mẫu thử: Không có sự khác biệt có ý nghĩa về các chỉ số liên quan đến chức năng thận giữa nhóm chứng và 2 nhóm thử ($P_{trướcTN\ (T-C)}$ **> 0,05**).

- Sau 14 ngày xịt mẫu thử và sau 28 ngày xịt mẫu thử: Không có sự khác biệt có ý nghĩa về các chỉ số liên quan đến chức năng thận giữa nhóm chứng và 2 nhóm thử ($P_{sau\ 14\ ngày(T-C)}$> 0,05; $P_{sau\ 28\ ngày(T-C)}$> 0,05).

3.5. Kết quả theo dõi chỉ số glucose trong huyết tương

a. Trước thử nghiệm (trước khi xịt mẫu thử)

Bảng 12. Chỉ số glucose trước khi dùng mẫu thử

Chỉ tiêu	Nhóm chứng (n = 7)	Nhóm T1 (n = 7)	$P_{(T1-C)}$	Nhóm T2 (n = 7)	$P_{(T2-C)}$
**Glucose** *(mmol/l)*	6,0 ± 1,0	5,8 ± 1,0	> 0,05	6,1 ± 0,6	> 0,05

b. Thời điểm sau 14 ngày xịt mẫu thử

Bảng 13. Chỉ số glucose sau 14 ngày dùng mẫu thử

Chỉ tiêu	Nhóm chứng (n = 7)	Nhóm T1 (n = 7)	$P_{(T1-C)}$	Nhóm T2 (n = 7)	$P_{(T2-C)}$
**Glucose** *(mmol/l)*	5,6 ± 1,0	5,6 ± 0,8	> 0,05	5,5 ± 0,9	> 0,05

c. Thời điểm sau 28 ngày xịt mẫu thử

Bảng 14. Chỉ số glucose sau 28 ngày dùng mẫu thử

Chỉ tiêu	Nhóm chứng (n = 7)	Nhóm T1 (n = 7)	$P_{(T1-C)}$	Nhóm T2 (n = 7)	$P_{(T2-C)}$
**Glucose** *(mmol/l)*	5,4 ± 0,6	5,3 ± 0,5	> 0,05	5,1 ± 0,7	> 0,05

Nhận xét

Kết quả xét nghiệm glucose cho thấy:

- Trước khi xịt mẫu thử: Không có sự khác biệt có ý nghĩa về chỉ số glucose giữa nhóm chứng và 2 nhóm thử ($P_{trướcTN\ (T-C)} > 0,05$).

- Sau 14 ngày xịt mẫu thử và sau 28 ngày xịt mẫu thử: Không có sự khác biệt có ý nghĩa về các chỉ số glucose giữa nhóm chứng và 2 nhóm thử ($P_{sau\ 14\ ngày(T-C)} >$ 0,05; $P_{sau\ 28\ ngày(T-C)} >$ 0,05).

3.6. Quan sát đại thể

Kết quả quan sát đại thể các cơ quan nội tạng của tất cả các thỏ thử nghiệm cho thấy: Không có biểu hiện khác thường về hình dạng bên ngoài, màu sắc của các tổ chức tim, gan, thận, phổi, dạ dày, ruột, âm đạo của các thỏ nhóm thử so với nhóm chứng sau thử nghiệm.

Bảng 15. Hình ảnh đại thể các cơ quan nội tạng

**Hình ảnh đại thể**		
Nhóm Chứng	***Nhóm Thử 1 (liều thấp)***	***Nhóm Thử 2 (liều cao)***
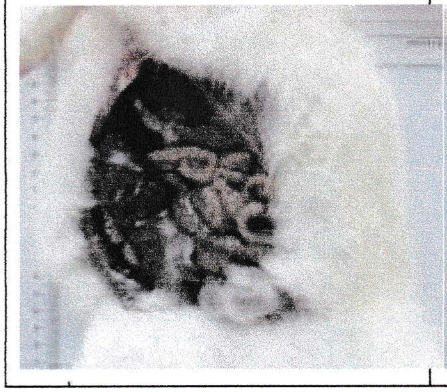	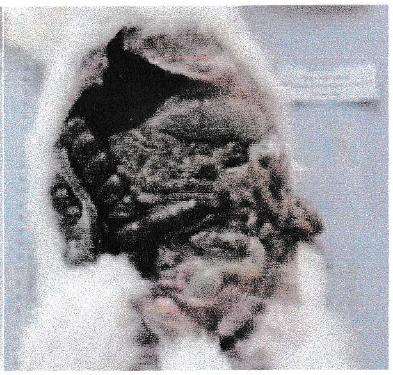	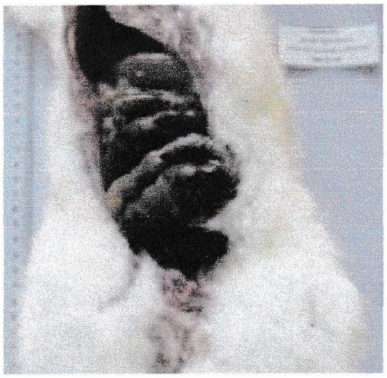

Bảng 16. Hình ảnh tim, gan, lách, thận và âm đạo

**Hình ảnh tim, gan, lách, thận và âm đạo**		
Nhóm Chứng	***Nhóm Thử 1 (liều thấp)***	***Nhóm Thử 2 (liều cao)***
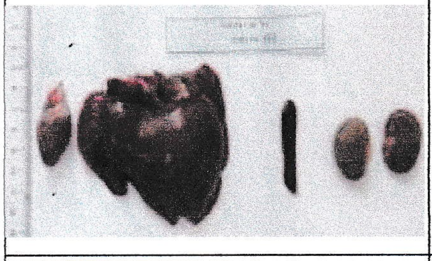	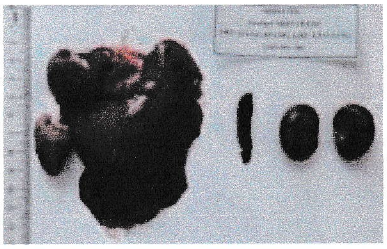	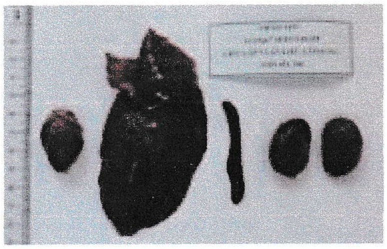
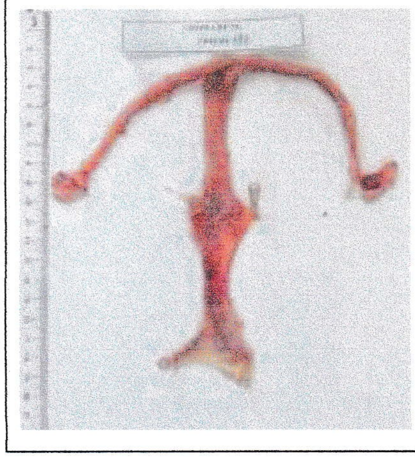	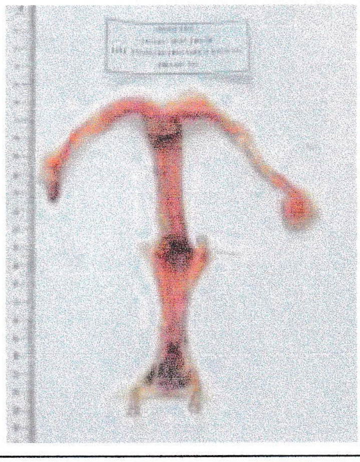	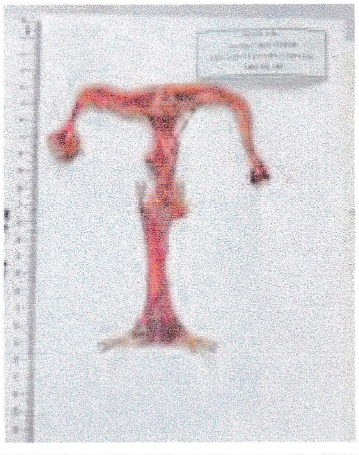

3.7. Quan sát vi thể

Tiêu bản gan, thận, âm đạo được cố định bằng Formalin 10%, nhuộm bằng dung dịch nhuộm Hematoxylin eosin (HE) và quan sát dưới kính hiển vi quang học.

Kết quả quan sát vi thể do Khoa Giải phẫu sinh lý bệnh – Bệnh viện Quân Y 103 thực hiện cho thấy: Các thỏ thí nghiệm đều có gan, thận, âm đạo không bị tổn thương, hình ảnh cấu trúc trong giới hạn bình thường. Không có các triệu chứng bất thường liên quan đến mẫu thử với 2 mức liều khác nhau so với nhóm chứng.

Cụ thể như sau:

Gan:

- Tế bào gan: Bình thường.
- Mao mạch nan hoa: Bình thường.
- Tĩnh mạch trung tâm: Bình thường.
- Khoảng cửa: Bình thường.
- Mô kẽ: Bình thường.

Kết luận: Mô gan bình thường, không thấy tổn thương.

Thận:

- Tiểu cầu thận (mao mạch, gian mạch, bao Baomann): Bình thường.
- Ống thận: Bình thường.
- Đài bể thận: Bình thường.
- Mô kẽ: Bình thường.

Kết luận: Mô thận bình thường, không thấy tổn thương

Âm đạo:

- Biểu mô: Thoái hóa nhẹ
- Thâm nhiễm bạch cầu: Rất nhẹ
- Mạch máu sung huyết: Không
- Dấu hiệu phù nề: Không có

Kết luận: Mô âm đạo bình thường, không nhận thấy tổn thương.

Bảng 17. Hình ảnh giải phẫu mô bệnh học gan và thận

(Hình ảnh nhuộm HE, Độ phóng đại 400 lần)

Nhóm	***Hình ảnh mô gan***	***Hình ảnh mô thận***	***Hình ảnh mô âm đạo***
Chứng	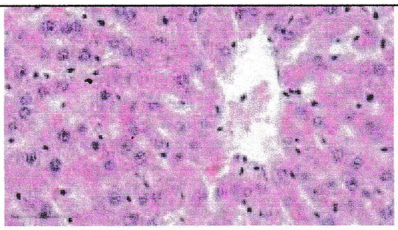 Mô gan bình thường, không thấy tổn thương.	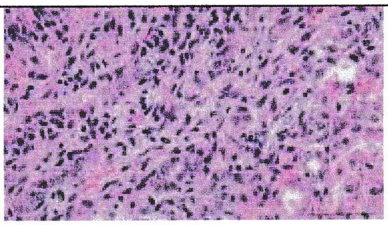 Mô thận bình thường, không thấy tổn thương.	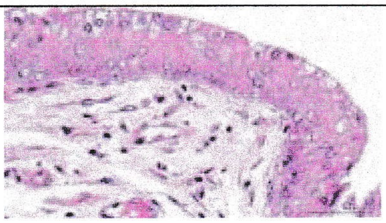 Mô âm đạo bình thường, không thấy tổn thương.
Thử 1 (liều thấp)	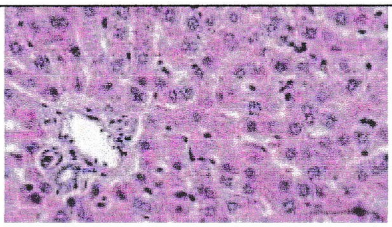 Mô gan bình thường, không thấy tổn thương.	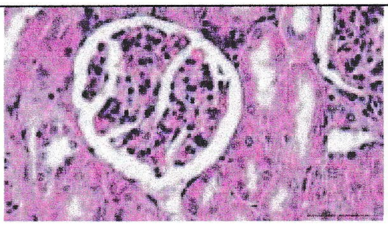 Mô thận bình thường, không thấy tổn thương.	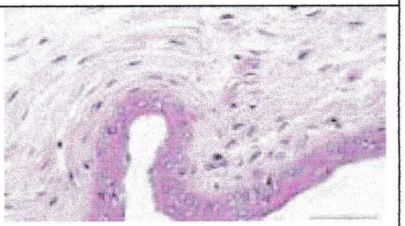 Mô âm đạo bình thường, không thấy tổn thương.
Thử 2 (liều cao)	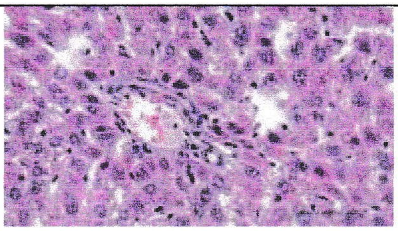 Mô gan bình thường, không thấy tổn thương.	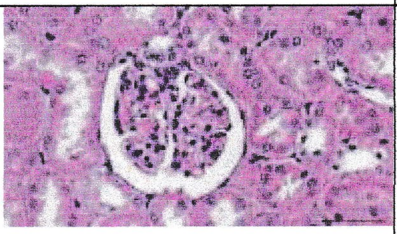 Mô thận bình thường, không thấy tổn thương.	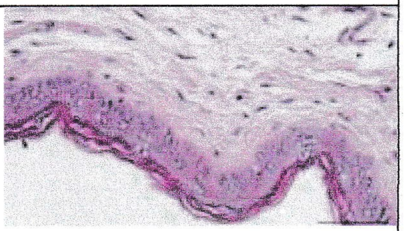 Mô âm đạo bình thường, không thấy tổn thương.

4. Kết luận

Mẫu thử LiveSpo® X-SECRET gửi tới yêu cầu thử độc tính bán trường diễn trên thỏ có kết quả như sau:

Sau khi cho thỏ dùng hỗn dịch mẫu thử liên tục trong 28 ngày với 2 mức liều khác nhau là 0,558 nhát/kg thỏ/ngày (tương ứng với mức liều tối đa dùng cho người là 9 nhát/người/ngày hay tương đương với ≥ $7,2 \times 10^8$ CFU *Bacillus subtilis, Bacillus clausii* và *Bacillus coagulans*/người/ngày) và 2,790 nhát/kg thỏ/ngày (cao gấp 5 lần so với liều dùng tối đa ngoại suy cho người, tương đương với ≥ $3,6 \times 10^9$ CFU *Bacillus subtilis, Bacillus clausii* và *Bacillus coagulans*/người/ngày), mẫu thử không ảnh

hưởng đến cân nặng, thể trạng, hoạt động của thỏ thử nghiệm. Thỏ khỏe mạnh, tăng cân.

Về các chỉ số sinh hóa đánh giá chức năng gan, thận (hoạt độ enzyme AST, ALT, albumin, bilirubin toàn phần, cholesterol, urê, creatinin), glucose và các chỉ số huyết học (hồng cầu, hemoglobin, hematocrit, bạch cầu, tiểu cầu) không có sự khác biệt có ý nghĩa ở trước thử nghiệm, sau 14 ngày và 28 ngày xịt mẫu thử giữa hai nhóm thử nghiệm so với nhóm chứng.

Không nhận thấy bất thường ở các tổ chức tim, gan, thận, phổi, lách và hệ tiêu hóa và âm đạo của thỏ thí nghiệm khi quan sát đại thể cũng như không nhận thấy tổn thương mô bệnh học của gan, thận và âm đạo khi quan sát vi thể giữa hai nhóm thử nghiệm và nhóm chứng.

Hà Nội, ngày 22 tháng 10 năm 2024

Xác nhận của cơ quan	Đại diện nhóm thực hiện
	Khoa Dược lý
VIỆN TRƯỞNG	
BỘ Y TẾ – VIỆN KIỂM NGHIỆM THUỐC TRUNG ƯƠNG	
PGS.TS. Đoàn Cao Sơn	Th.S. Trần Thị Thanh Huế
