# Supplementary material for: Vaginal-spray Bacillus spore probiotics as a potential treatment and reducing recurrence of bacterial vaginosis: randomized, double-blind, and controlled pilot study
Source: Commun Med (Lond). 2025 Nov 18;5:527. doi: 10.1038/s43856-025-01236-4 (PMC12717237; doi:10.1038/s43856-025-01236-4)
Supplement: Supplementary file 4 — Supplementary Data 2 [file 43856_2025_1236_MOESM4_ESM.pdf]

NATIONAL INSTITUTE OF DRLTG QUALITY CONTROL

LABORATORY OF PHARNIACOLOGY

TEST RESULTS

VAGINAL MUCOSAL IRRITATION TEST

(Test results are valid for the test samples only)

A. GENERAL INFORMATION

Sample Name:	LiveSpo® X-SECRET
Manufacturer:	LiveSpo Pharma Co., Ltd.
Batch No.:	H623X
Manufacture date:	17/06/2024
Expiry date:	16/06/2026
Sample sender::	LiveSpo Pharma Co., Ltd.
Test criteria::	Vaginal mucosal irritation test
Test documents:	ISO 10993-23:2021. Biological evaluation of medical devices. Tests for irritation
Labeled formulation:	LiveSpo® X-SECRET probiotic product contains *Bacillus subtilis*, *Bacillus clausii*, and *Bacillus coagulans* at a concentration of >1 billion CFU/ml.
Sample description:	Light brown-yellow solution in 360° rotating bottles, labeled with product name, volume, manufacturer, manufacturing and expiry dates, and usage instructions.
Testing department:	Laboratory of Pharmacology – National Institute of Drug Quality Control
Test conductors:	Nguyen Thi Hong Vinh<br>Nguyen Duc Long<br>Truong Thi Hang

B. TESTING INFORMATION

Implementation period: From 15/09/2024 to 20/09/2024

1. Experimental animals

- Species, sex: Healthy female New Zealand white rabbits, weighing no less than 2 kg, with a fully developed reproductive system, not previously bred, and not pregnant.
- Quantity: 6 rabbits.
    - Test group: 3 rabbits
    - Control group: 3 rabbits
- Source: Laboratory of Pharmacology – National Institute of Drug Quality Control.

- Care conditions: Rabbits were housed individually in cages under controlled temperature and humidity, with food and water provided ad libitum. All handling and care of the test animals adhered to the care and use procedures of the Laboratory of Pharmacology – National Institute of Drug Quality Control.

Prior to testing, the rabbits' vaginas were examined to exclude those with abnormal signs such as swelling, infection, irritation, or lesions. Testing was scheduled to avoid the estrous cycle to prevent false-positive results.

2. Experiment

2.1. Preparation of the test samples

- Sample preparation: The test product was used in its original form (contained in a spray bottle with a nozzle approximately 6 cm in length).
- Animal preparation: Rabbits were restrained using a specialized device, with their hind legs held to expose the genital area.
- Test dose: 1.0 ml of the test product per rabbit per day (equivalent to >1 x 10^9 probiotic spores per rabbit per day).

2.2. Procedure:

Hold the rabbit's tail to expose the vaginal area. Gently insert the spray nozzle deep into the rabbit's vagina and administer 14 sprays, equivalent to 1 ml of the test product (>1 x 10^9 probiotic spores). Carefully withdraw the spray nozzle to avoid causing irritation. Due to variations in vaginal size, some of the test product may leak out during or after administration. Gently remove any excess product using a soft gauze. Repeat the procedure every 24 ± 2 hours for 5 consecutive days.

2.3. Monitoring and assessment

After administration, the rabbits were returned to individual cages and cared for under normal conditions.

* Observation and evaluation

- *External observation*
  - Inspect and record any external signs on the vaginal area, perineum, and between the vagina and anus, such as discharge, erythema, or edema, immediately before and 24 ± 2 hours after each application.
  - Rabbits exhibiting excessive discharge, severe erythema, edema, or a combination of both, making further application difficult, will be humanely euthanized for histopathological examination.
- *Gross observation*
  - At 24 ± 2 hours after the final dose, euthanize the rabbits humanely and collect the entire vaginal tissue. Longitudinally open the vagina and examine for signs of irritation, epithelial damage in soft tissues, and necrosis.
  - Compare the vaginal tissues of the test and control groups. Document and describe any gross differences observed between the groups.
- *Microscopic observation*

Prepare histological slides of the rabbits' vaginal tissues. Perform microscopic observations and evaluations on all rabbits in both the test and control groups.

Table 1. Overall assessment scoring

No.	Reaction	Point ladder
**Epithelium**		
1	Normal, intact	0
2	Cellular degeneration or flattening	1
3	Dysplasia	2
4	Focal erosion	3
5	Extensive erosion	4

**Leukocyte infiltration (Observed in high-power fields)**		
1	No infiltration	0
2	Very mild infiltration (fewer than 25)	1
3	Mild infiltration (26–50)	2
4	Moderate infiltration (51–100)	3
5	Severe infiltration (more than 100)	4
**Congestion**		
1	No congestion	0
2	Very mild congestion	1
3	Mild congestion	2
4	Moderate obstruction	3
5	Severe congestion with vascular destruction	4
**Edema**		
1	No edema	0
2	Very mild edema	1
3	Mild edema	2
4	Moderate edema	3
5	Severe edema	4

Definitions:

- Average reaction score of the Test group: This is calculated by summing the reaction scores of all rabbits in the test group and dividing by the number of rabbits in the group. The maximum reaction score is 16. The reaction score for the control group is calculated in the same manner as the test group.
- If the total reaction score of any rabbit in the control group exceeds 9, it indicates that the act of sample administration may have caused damage to the rabbit. In such

cases, the experiment may need to be repeated if other rabbits in both the control and test groups also show similarly high scores.

- Irritation score of the Test sample: This is determined by subtracting the average reaction score of the control group from the average reaction score of the test group.

Based on the reaction score of the test sample, the level of irritation is determined according to the criteria specified in Table 2.

Table 2. Evaluation of vaginal mucosal irritation levels in rabbits

No.	Irritation Score	Description of the reaction
1	0	No irritation
2	1 – 4	Very mild irritation
3	5 – 8	Mild irritation
4	9 – 11	Moderate irritation
5	12 – 16	Severe irritation

3. Results

3.1. Observation of external manifestations

The observations of the rabbits' vaginal area after exposure to the test sample are presented in Table 3

Table 3. Observed external manifestations on rabbits' vaginal area

**Time point**	**Control group**			**Test group**		
	Rabbit No. 1- 176 (2.124 kg)	Rabbit No. 2- 200 (2.235 kg)	Rabbit No. 3- 201 (2.304 kg)	Rabbit No. 1- 181 (2.268 kg)	Rabbit No. 2- 199 (2.175 kg)	Rabbit No. 3- 184 (2.304 kg)
Before testing	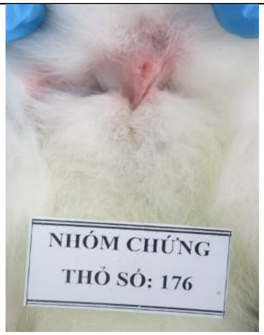	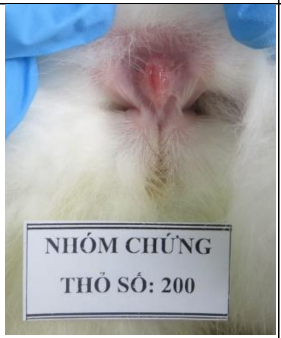	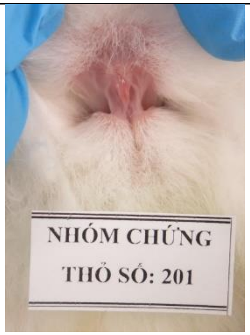	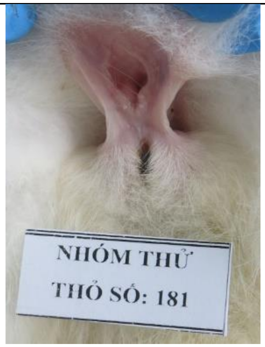	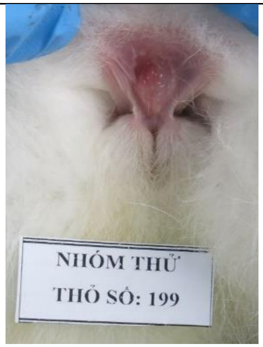	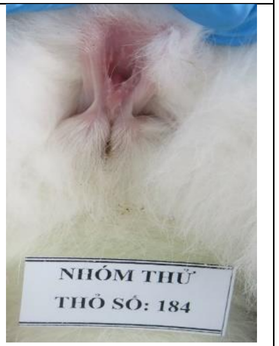
	The vaginal mucosa was pale pink, with no erythema, edema, discharge, or lesions.					
After the first application	No abnormal manifestations	No abnormal manifestations	No abnormal manifestations	No abnormal manifestations	No abnormal manifestations	No abnormal manifestations
Before the second application	No abnormal manifestations	No abnormal manifestations	No abnormal manifestations	No abnormal manifestations	No abnormal manifestations	No abnormal manifestations
After the second application	No abnormal manifestations	No abnormal manifestations	No abnormal manifestations	No abnormal manifestations	No abnormal manifestations	No abnormal manifestations

Before the third application	No abnormal manifestations	No abnormal manifestations	No abnormal manifestations	No abnormal manifestations	No abnormal manifestations	No abnormal manifestations
After the third application	No abnormal manifestations	No abnormal manifestations	No abnormal manifestations	No abnormal manifestations	No abnormal manifestations	No abnormal manifestations
Before the fourth application	No abnormal manifestations	No abnormal manifestations	No abnormal manifestations	No abnormal manifestations	No abnormal manifestations	No abnormal manifestations
After the fourth application	No abnormal manifestations	No abnormal manifestations	No abnormal manifestations	No abnormal manifestations	No abnormal manifestations	No abnormal manifestations
Before the fifth application	No abnormal manifestations	No abnormal manifestations	No abnormal manifestations	No abnormal manifestations	No abnormal manifestations	No abnormal manifestations
After the fifth application	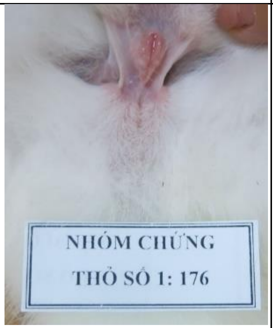	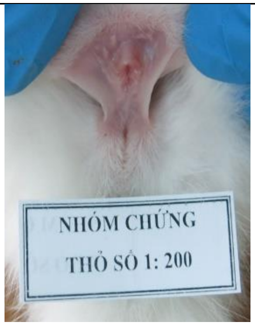	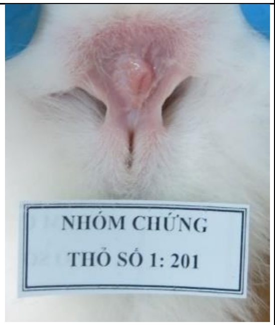	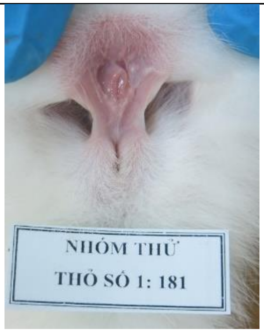	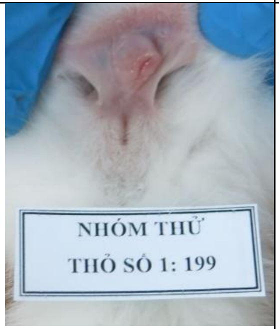	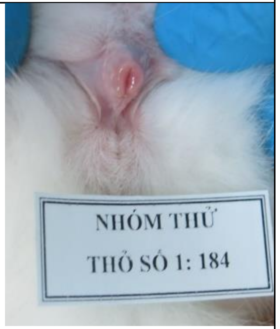
	The vaginal mucosa was pale pink, with no erythema, edema, or lesions.					

3.2. Gross observation results

Gross observations revealed: Control Group: Rabbit No. 200 exhibited mild congestion, and Rabbit No. 176 showed very mild congestion and edema. Test Group: Rabbit No. 184 and Rabbit No. 199 exhibited mild congestion but no edema. The remaining rabbits in both

the control and test groups had smooth mucosal surfaces, pale pink coloration, and no signs of congestion, edema, or surface lesions. A comparison of gross observations between the test and control groups showed no mucosal damage or necrosis in either group. The degree of congestion in the test group was mild and comparable to that in the control group, with no significant differences..

Gross images of the vaginal mucosa in rabbits are presented in Table 4.

Table 4. Gross observation images of vaginal mucosa

Group	Rabbit 1	Rabbit 2	Rabbit 3
**Control group**	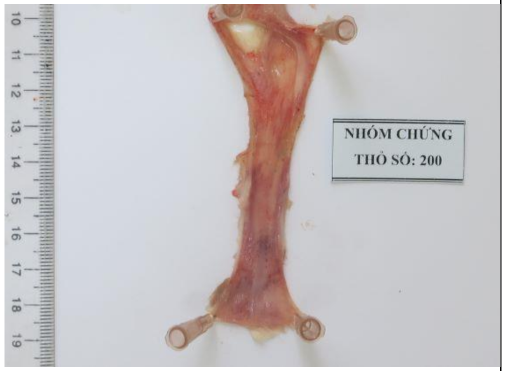	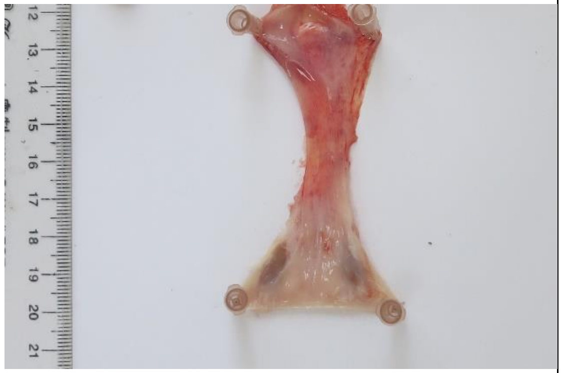	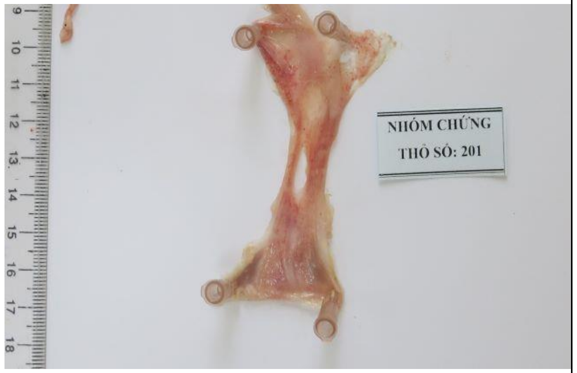

**Test group**	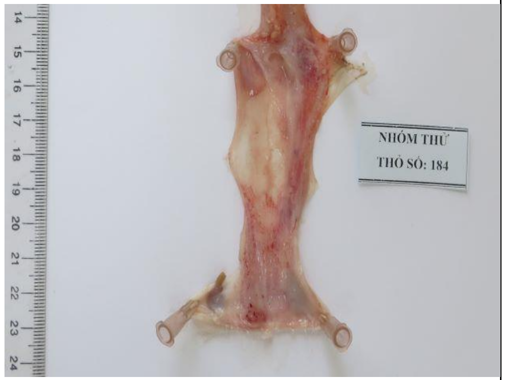	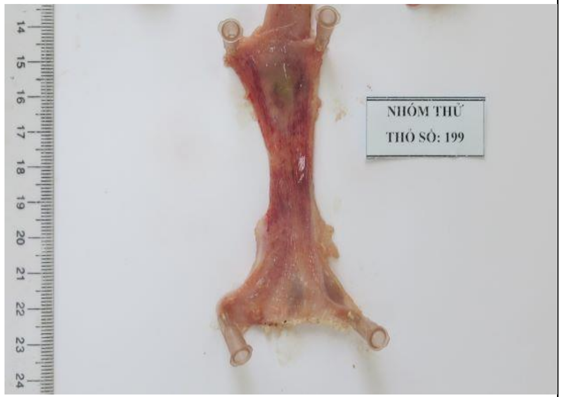	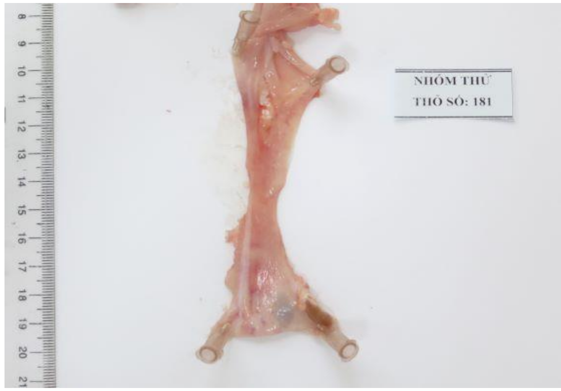

Translated by Nguyen Thi Van Anh, corresponding author

3.3. Microscopic observation results

The vaginal specimens were fixed in 10% Formalin, stained with Hematoxylin and Eosin (HE) staining solution, and observed under a light microscope.

The microscopic observation results were conducted by the Department of Pathological Anatomy – Military Medical Hospital 103. The microscopic observation scores of the rabbit vaginal mucosa after exposure to the test sample are presented in Table 5.

Table 5. Microscopic observation scores of rabbit vaginal mucosa

Group	Rabbit	Epithelium	Leukocyte infiltration	Congestion	Edema	Total score
**Control**	Rabbit No. 200	1	1	1	0	3
	Rabbit No. 176	1	1	1	1	4
	Rabbit No. 201	1	1	0	0	2
	**Average**					**3**
**Test**	Rabbit No. 184	1	2	1	0	4
	Rabbit No. 199	1	2	1	0	4
	Rabbit No. 181	1	1	0	0	2
	**Average**					**3.3**
**Irritation Score**						**0.3**

Microscopic images of the rabbit vaginal tissue are shown in Table 6.

Table 6. Histopathological Images of Rabbit Vaginal Mucosa

No.	Control Group	Test Group
1	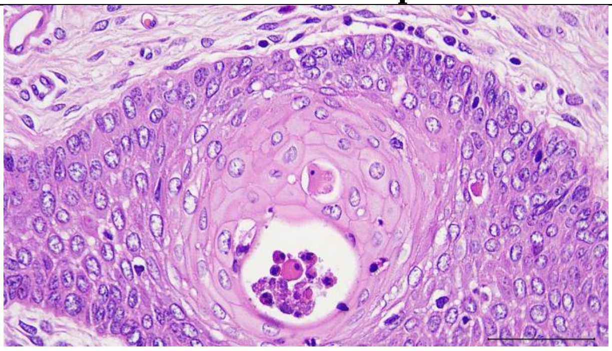<br>**Rabbit No. 200:** Mild epithelial cell degeneration, very mild leukocyte infiltration, very mild vascular congestion, no edema in the stroma	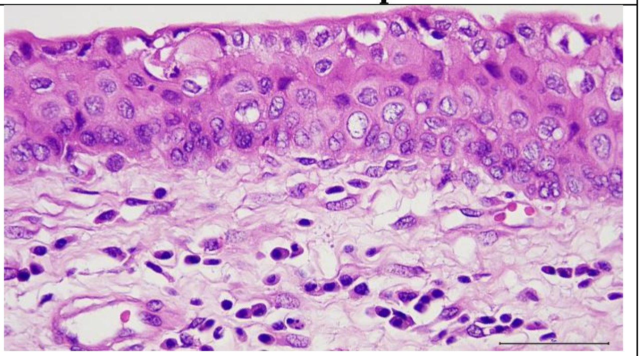<br>**Rabbit No. 184:** Mild epithelial cell degeneration, mild leukocyte infiltration, vascular congestion, no edema in the stroma
2	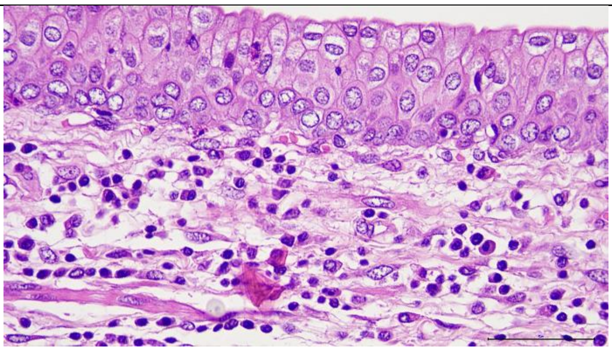<br>**Rabbit No. 176:** Mild epithelial cell degeneration, very mild leukocyte infiltration, very mild vascular congestion, very mild edema in the stroma	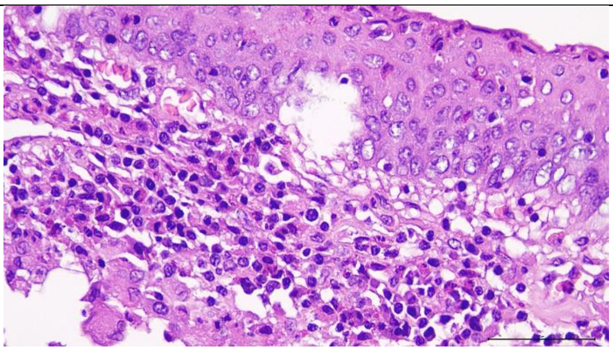<br>**Rabbit No. 199:** Mild epithelial cell degeneration, mild leukocyte infiltration, vascular congestion, no edema in the stroma
3	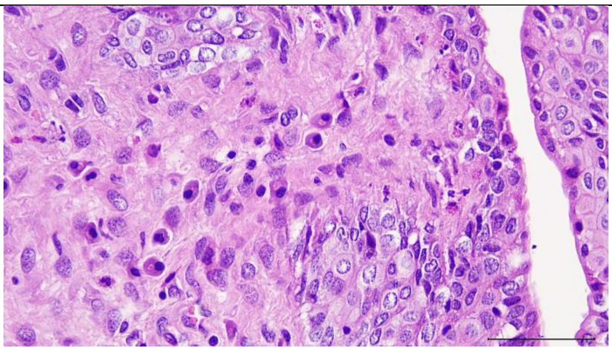<br>**Rabbit No. 201:** Mild epithelial cell degeneration, very mild leukocyte infiltration, no vascular congestion, no edema in the stroma	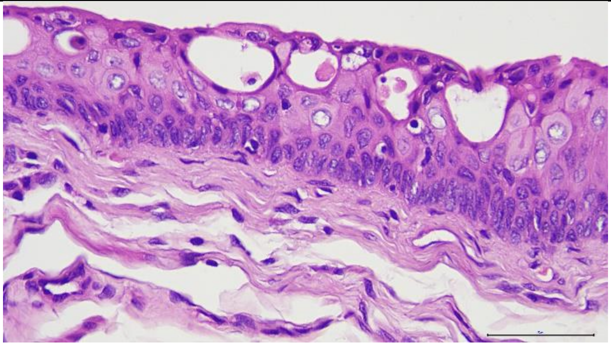<br>**Rabbit No. 181:** Mild epithelial cell degeneration, very mild leukocyte infiltration, no vascular congestion, no edema in the stroma

3.4. Classification

Based on the classification guidelines for oral mucosal irritation level according to ISO 10993-23: 2021, and the results obtained from the experiment, the test sample is classified as non-irritating to the vaginal mucosa.

C. CONCLUSION

The test sample LiveSpo® X-SECRET, submitted for vaginal irritation testing in rabbits according to ISO 10993-23:2021 showed the following results:

The test sample was applied to the vaginal mucosa of rabbits at a dose of 1 sample/rabbit/day (equivalent to >1 × 10^9 CFU *Bacillus subtilis*, *Bacillus clausii*, and *Bacillus coagulans*/rabbit/day) for five consecutive days, with a single application each day at a fixed time. Experimental results demonstrated that the test sample did not cause vaginal irritation.

Hanoi, October 22, 2024

Certified by the Head of Organization	Laboratory of pharmacology
**(signed and stamped)**	**(Signed)**
**Assoc. Prof. Doan Cao Son**	**MSc. Tran Thi Thanh Hue**

Translated by Nguyen Thi Van Anh, corresponding author

VIỆN KIỂM NGHIỆM THUỐC TW

KHOA DƯỢC LÝ

BÁO CÁO KẾT QUẢ THỬ NGHIỆM KÍCH ỨNG NIÊM MẠC ÂM ĐẠO

(Kết quả thử nghiệm chỉ có giá trị với mẫu đem thử)

A. THÔNG TIN CHUNG

Tên mẫu:	LiveSpo® X-SECRET
Nhà sản xuất:	Công ty TNHH LiveSpo Pharma
Lô sản xuất:	H623X
Ngày sản xuất:	17/06/2024
Hạn dùng:	16/06/2026
Nơi gửi mẫu:	Công ty TNHH LiveSpo Pharma
Chỉ tiêu thử:	Kích ứng âm đạo
Tài liệu thử:	Đánh giá tác dụng sinh học của thiết bị y tế theo ISO 10993-23-2021. Thử nghiệm kích ứng<br>ISO 10993-23: 2021. Biological evaluation of medical devices. Tests for irritation
Công thức bào chế trên nhãn:	*B. subtilis, B. clausii và B. coagulans* nồng độ $\geq$ 1 tỷ CFU/ml cho sản phẩm probiotic LiveSpo® X-SECRET.
Tình trạng mẫu khi mở niêm phong để thử nghiệm:	Mẫu thử dạng hỗn dịch màu vàng nâu nhạt, được đóng trong bình xoay 360 độ có dán nhãn, trên nhãn ghi tên mẫu, thể tích, nơi sản xuất, ngày sản xuất, hạn dùng, hướng dẫn sử dụng.
Khoa thử nghiệm:	Khoa Dược lý - Viện Kiểm nghiệm thuốc Trung Ương
Người thực hiện:	Nguyễn Thị Hồng Vinh<br>Nguyễn Đức Long<br>Trương Thị Hằng

B. THÔNG TIN THỬ NGHIỆM

Thời gian thực hiện: Từ 15/09/2024 đến 20/09/2024

1. Động vật thí nghiệm

- Thỏ trắng Newzealand khỏe mạnh, giống cái, cân nặng không dưới 2 kg, hệ sinh sản phát triển, thỏ chưa sinh đẻ và đang không có thai.
- Số lượng: 06 con.
    - Nhóm thử: 03 con
    - Nhóm chứng: 03 con
- Nguồn gốc: Khoa Dược lý - Viện Kiểm nghiệm thuốc Trung ương.
- Điều kiện chăm sóc: Thỏ được nuôi mỗi con một lồng trong phòng nuôi có kiểm soát nhiệt độ và độ ẩm thích hợp với thức ăn và nước uống theo nhu cầu. Tất cả các thao tác trên động vật thí nghiệm đều được tuân theo các quy trình về chăm sóc và sử dụng động vật thí nghiệm của Khoa Dược lý – Viện Kiểm nghiệm thuốc Trung Ương.

Trước thử nghiệm thỏ được kiểm tra âm đạo để loại bỏ các thỏ có biểu hiện bất thường như sưng, viêm nhiễm, kích ứng, tổn thương.... Chú ý thời điểm tiến hành thử nghiệm không trùng với chu kỳ động dục của thỏ do có thể gây kết quả dương tính giả.

2. Phương pháp thử nghiệm

- Chuẩn bị mẫu thử: Dùng nguyên mẫu (mẫu thử được đựng trong bình xịt có vòi xịt dài khoảng 6 cm).

- Chuẩn bị động vật thử nghiệm: cố định thỏ bằng dụng cụ chuyên biệt, giữ 2 chân sau để bộc lộ bộ phận sinh dục của thỏ.

- Mức liều thử nghiệm: 1,0 ml mẫu thử/thỏ/ngày (tương đương $\geq 1 \times 10^9$ bào tử lợi khuẩn/thỏ/ngày) trong 5 ngày liên tiếp.

- Tiến hành: Nắm đuôi thỏ để bộc lộ âm đạo, nhẹ nhàng luồn vòi xịt vào sâu trong âm đạo thỏ. Xịt 14 nhát tương đương với 1 ml mẫu thử (tương đương $\geq 1 \times 10^9$ bào tử lợi khuẩn) vào âm đạo thỏ, nhẹ nhàng rút vòi xịt ra khỏi âm đạo thỏ. Trong quá trình thực hiện cần nhẹ nhàng để tránh gây kích ứng âm đạo thỏ. Do sự khác nhau về kích thước âm đạo của thỏ mà trong quá trình xịt hoặc sau khi xịt, mẫu thử có thể bị chảy ra ngoài, nhẹ

nhàng loại bỏ mẫu thử chảy ra bằng gạc mềm. Tiến hành tương tự như trên với chu kỳ (24 ± 2) giờ trong 5 ngày liên tiếp.

- Sau khi xịt thuốc xong, đưa thỏ về chuồng nhốt riêng, chăm sóc thỏ theo điều kiện bình thường.

- Quan sát và đánh giá

+ Quan sát các biểu hiện bên ngoài

- Quan sát và ghi lại các biểu hiện phía ngoài của âm đạo, phần giữa âm đạo và hậu môn về các dấu hiệu như chảy dịch, ban đỏ, phù nề ở thời điểm ngay trước và (24 ± 2) giờ sau mỗi lần dùng mẫu thử.
- Động vật có biểu hiện chảy dịch quá mức hoặc ban đỏ hay phù nề hoặc cả ban đỏ và phù nề dẫn tới khó trong việc dùng mẫu thì sẽ được gây chết nhân đạo và kiểm tra giải phẫu mô bệnh học.

+ Quan sát đại thể

- (24 ± 2) giờ sau khi dùng liều cuối cùng, gây chết nhân đạo cho động vật thí nghiệm, mổ lấy toàn bộ âm đạo, mở dọc âm đạo và kiểm tra các dấu hiệu kích ứng, tổn thương ở lớp biểu mô của mô mềm và dấu hiệu hoại tử
- So sánh âm đạo của thỏ ở nhóm thử và nhóm chứng: Ghi và mô tả các quan sát đại thể về mô âm đạo của mỗi động vật, chú ý sự khác biệt giữa nhóm thử và nhóm chứng.

+ Quan sát vi thể: Làm tiêu bản vi thể âm đạo thỏ

Tiến hành quan sát và đánh giá trên tất cả thỏ ở nhóm thử và nhóm chứng

Điểm phản ứng được đánh giá theo các tiêu chí nêu trong Bảng 1.

Bảng 1. Hệ thống chấm điểm mô bệnh học

Số TT	Phản ứng	Thang điểm
**Biểu mô**		
1	Bình thường, nguyên vẹn	0
2	Thoái hóa tế bào hoặc làm dẹt tế bào	1
3	Dị sản	2
4	Trợt điểm	3
5	Trợt lan rộng	4
**Thâm nhiễm bạch cầu (Quan sát trên vi trường ở độ phóng đại lớn)**		
1	Không có sự thâm nhiễm	0
2	Thâm nhiễm rất nhẹ (dưới 25)	1
3	Thâm nhiễm nhẹ (26 – 50)	2
4	Thâm nhiễm vừa phải (51 - 100)	3
5	Thâm nhiễm nặng (lớn hơn 100)	4
**Sung huyết**		
1	Không sung huyết	0
2	Sung huyết rất nhẹ	1
3	Sung huyết nhẹ	2
4	Tắc nghẽn vừa phải	3
5	Sung huyết nặng, phá hủy mạch máu	4
**Phù nề**		
1	Không phù nề	0
2	Phù nề rất nhẹ	1
3	Phù nề nhẹ	2
4	Phù nề vừa phải	3
5	Phù nề nặng	4

+ Điểm phản ứng trung bình của nhóm thử: Được tính bằng tổng điểm phản ứng của các thỏ trong nhóm thử chia cho số lượng thỏ của nhóm thử. Điểm phản ứng tối đa là 16. Cách tính điểm phản ứng của nhóm chứng tương tự như nhóm thử

+ Nếu tổng điểm phản ứng ở một thỏ của nhóm chứng lớn hơn 9 cho thấy có khả năng động tác đưa mẫu có thể gây tổn thương cho thỏ và có thể phải tiến hành lại thử nghiệm nếu những thỏ khác ở nhóm chứng và nhóm thử cũng có diểm cao tương tự.

- Điểm kích ứng của mẫu thử: Được tính bằng cách lấy điểm phản ứng trung bình của nhóm thử trừ đi điểm phản ứng trung bình nhóm chứng.

- Dựa trên điểm phản ứng của mẫu thử, xác định mức độ kích ứng của mẫu thử theo quy định ở Bảng 2.

Bảng 2. Bảng đánh giá khả năng gây kích ứng niêm mạc âm đạo thỏ

**STT**	**Điểm kích ứng**	**Loại phản ứng**
1	0	Không kích ứng
2	1 – 4	Kích ứng rất nhẹ
3	5 – 8	Kích ứng nhẹ
4	9 – 11	Kích ứng vừa phải
5	12 – 16	Kích ứng nặng

3. Kết quả

3.1. Quan sát các biểu hiện bên ngoài

Các biểu hiện trên âm đạo thỏ sau khi tiếp xúc với mẫu thử được trình bày ở Bảng 3

Bảng 3. Các biểu hiện quan sát được trên âm đạo thỏ

**Thời điểm**	**Nhóm chứng**			**Nhóm thử**		
	Thỏ số 1 - 176 (2,268 kg)	Thỏ số 2 - 200 (2,175 kg)	Thỏ số 3 - 201 (2,304 kg)	Thỏ số 1 - 181 (2,124 kg)	Thỏ số 2 - 199 (2,235 kg)	Thỏ số 3 - 184 (2,304 kg)
Trước thử nghiệm	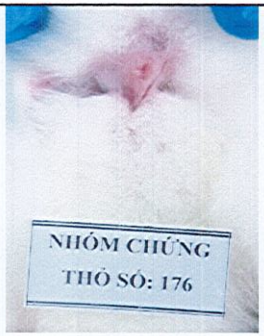	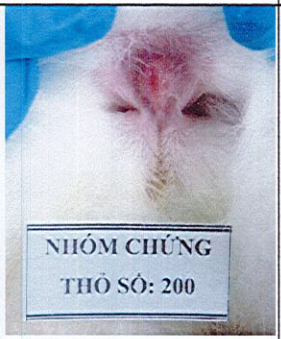	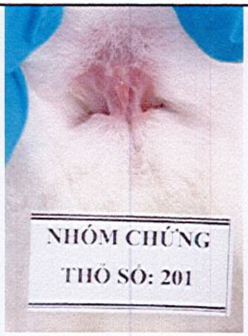	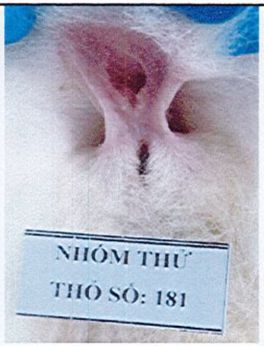	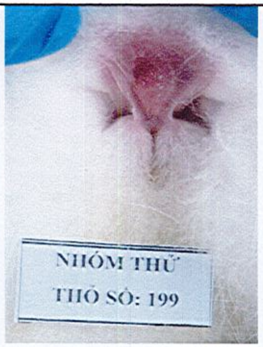	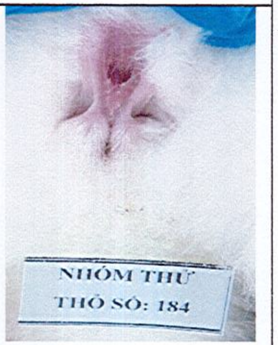
	Niêm mạc cửa âm đạo hồng nhạt, không có ban đỏ và phù nề, không chảy dịch, không có tổn thương					
Sau khi dùng mẫu thử lần 1	Không có biểu hiện bất thường	Không có biểu hiện bất thường	Không có biểu hiện bất thường	Không có biểu hiện bất thường	Không có biểu hiện bất thường	Không có biểu hiện bất thường
Trước khi dùng mẫu thử lần 2	Không có biểu hiện bất thường	Không có biểu hiện bất thường	Không có biểu hiện bất thường	Không có biểu hiện bất thường	Không có biểu hiện bất thường	Không có biểu hiện bất thường
Sau khi dùng mẫu thử lần 2	Không có biểu hiện bất thường	Không có biểu hiện bất thường	Không có biểu hiện bất thường	Không có biểu hiện bất thường	Không có biểu hiện bất thường	Không có biểu hiện bất thường

Trước khi dùng mẫu thử lần 3	Không có biểu hiện bất thường	Không có biểu hiện bất thường	Không có biểu hiện bất thường	Không có biểu hiện bất thường	Không có biểu hiện bất thường	Không có biểu hiện bất thường
Sau khi dùng mẫu thử lần 3	Không có biểu hiện bất thường	Không có biểu hiện bất thường	Không có biểu hiện bất thường	Không có biểu hiện bất thường	Không có biểu hiện bất thường	Không có biểu hiện bất thường
Trước khi dùng mẫu thử lần 4	Không có biểu hiện bất thường	Không có biểu hiện bất thường	Không có biểu hiện bất thường	Không có biểu hiện bất thường	Không có biểu hiện bất thường	Không có biểu hiện bất thường
Sau khi dùng mẫu thử lần 4	Không có biểu hiện bất thường	Không có biểu hiện bất thường	Không có biểu hiện bất thường	Không có biểu hiện bất thường	Không có biểu hiện bất thường	Không có biểu hiện bất thường
Trước khi dùng mẫu thử lần 5	Không có biểu hiện bất thường	Không có biểu hiện bất thường	Không có biểu hiện bất thường	Không có biểu hiện bất thường	Không có biểu hiện bất thường	Không có biểu hiện bất thường
Sau khi dùng mẫu thử lần 5	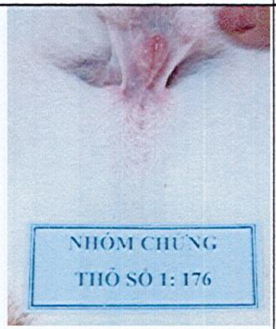	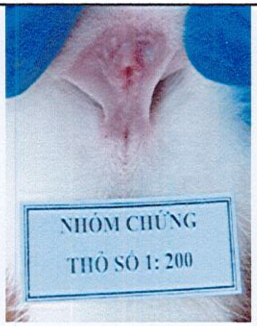	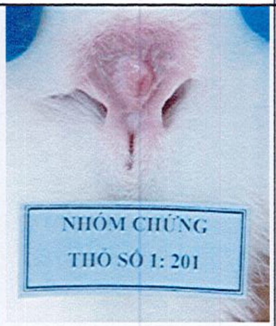	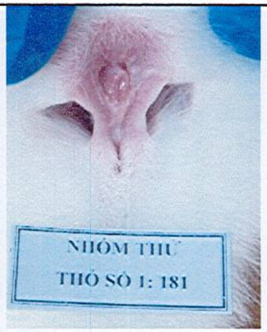	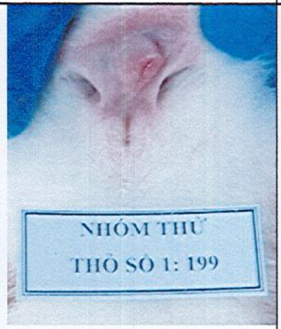	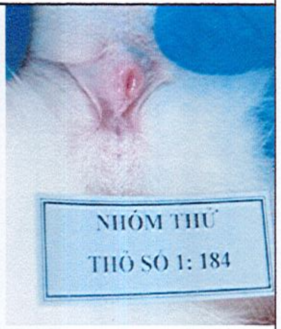
	Niêm mạc cửa âm đạo hồng nhạt, không có ban đỏ và phù nề, không tổn thương					

3.2. Kết quả quan đại thể

Quan sát đại thể cho thấy: Ở nhóm chứng có thỏ số 200 bị xung huyết nhẹ, thỏ 176 bị xung huyết và phù rất nhẹ, ở nhóm thử có thỏ 184 và thỏ 199 bị xung huyết nhẹ, nhưng không bị phù nề. Các thỏ chứng và thử còn lại có bề mặt niêm mạc nhẵn, màu hồng nhạt, không bị

xung huyết, phù nề và tổn thương bề mặt. So sánh quan sát đại thể giữa nhóm thử và nhóm chứng nhận thấy: bề mặt niêm mạc âm đạo ở cả hai nhóm đều không có tổn thương và không có hoại tử, mức độ xung huyết ở nhóm thử so với nhóm chứng là nhẹ và không khác biệt.

Hình ảnh quan sát đại thể âm đạo thỏ được trình bày ở bảng 4

Bảng 4. Hình ảnh quan sát đại thể âm đạo

Nhóm	Thỏ 1	Thỏ 2	Thỏ 3
**Chứng**	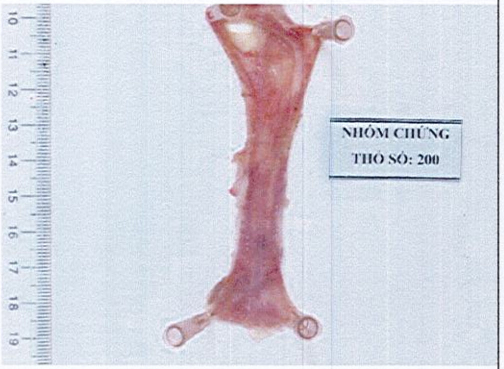	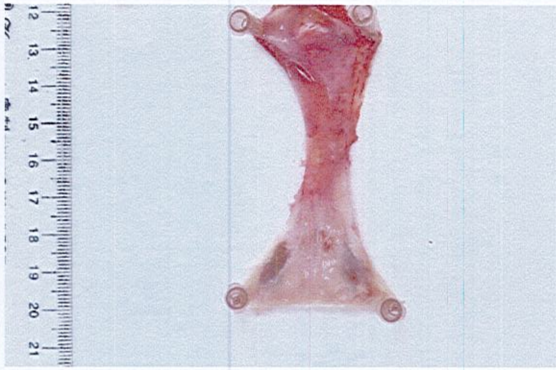	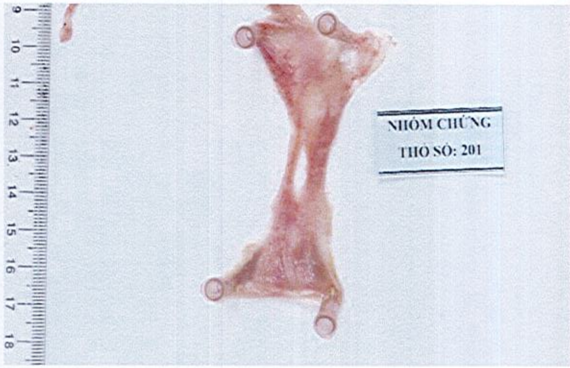
**Thử**	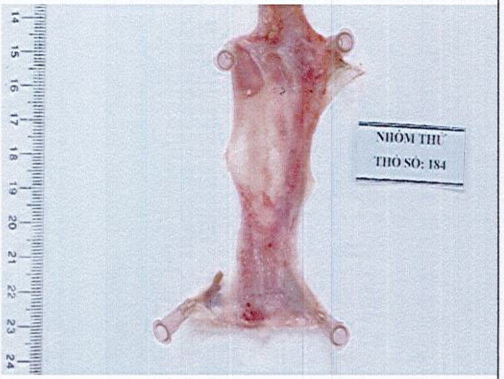	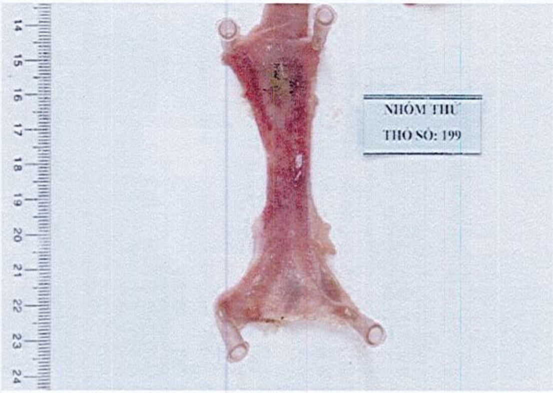	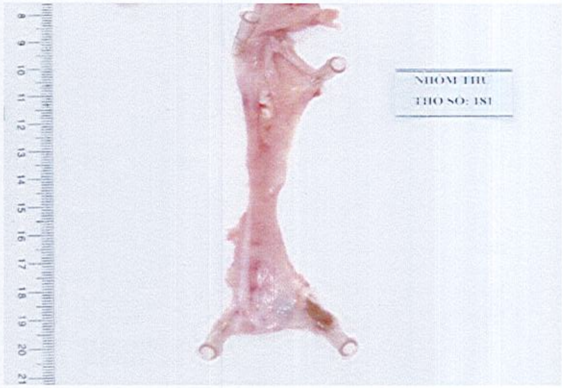

3.3. Kết quả quan sát vi thể

Tiêu bản âm đạo được cố định bằng Formalin 10%, nhuộm bằng dung dịch nhuộm Hematoxylin Eosin (HE) quan sát dưới kính hiển vi quang học.

Kết quả quan sát vi thể do Khoa Giải phẫu sinh lý bệnh – Bệnh viện Quân Y 103 thực hiện. Kết quả điểm quan sát vi thể âm đạo thỏ sau khi tiếp xúc mẫu thử được trình bày trong Bảng 5.

Hình ảnh vi thể mô âm đạo thỏ được trình bày ở Bảng 6

Bảng 5. Điểm quan sát vi thể âm đạo thỏ

Nhóm	Thỏ	Biểu mô	Thâm nhiễm bạch cầu	Sung huyết	Phù nề	Tổng điểm
Chứng	Thỏ số 200	1	1	1	0	3
	Thỏ số 176	1	1	1	1	4
	Thỏ số 201	1	1	0	0	2
	**Trung bình**					**3**
Thử	Thỏ số 184	1	2	1	0	4
	Thỏ số 199	1	2	1	0	4
	Thỏ số 181	1	1	0	0	2
	**Trung bình**					**3,3**
**Điểm kích ứng**						**0,3**

Bảng 6. Hình ảnh giải phẫu mô bệnh học âm đạo

STT	Nhóm chứng	Nhóm thử
1	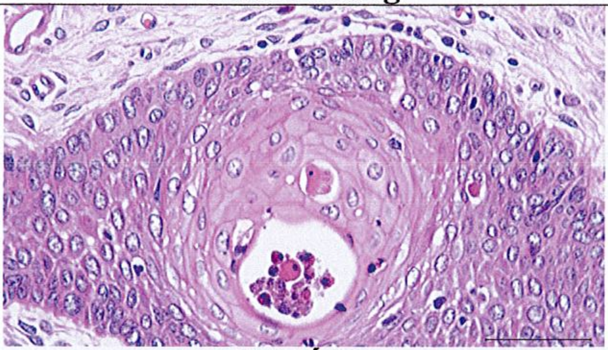<br>**Thỏ số 200**<br>Thoái hóa nhẹ tế bào biểu mô, thâm nhiễm bạch cầu mức độ rất nhẹ, mạch máu sung huyết rất nhẹ, mô đệm không phù nề	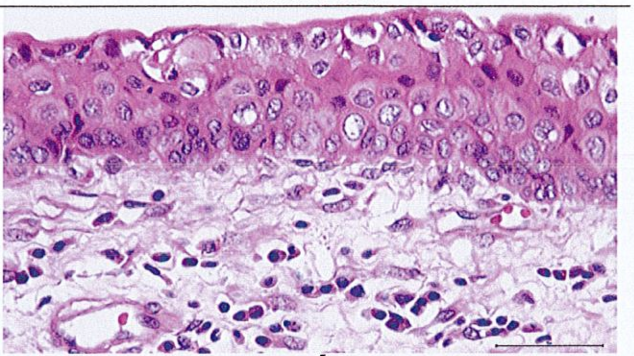<br>**Thỏ số 184**<br>Thoái hóa nhẹ tế bào biểu mô, thâm nhiễm bạch cầu nhẹ, mạch máu sung huyết và mô đệm không phù nề
2	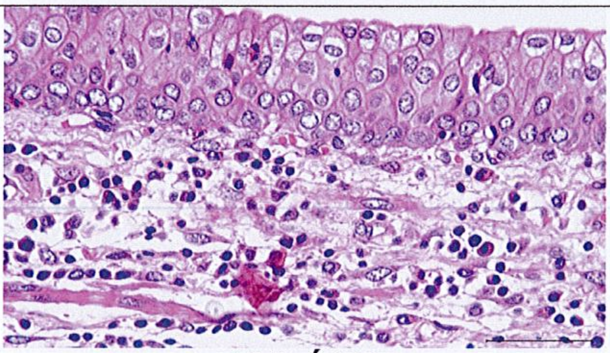<br>**Thỏ số 176**<br>Thoái hóa nhẹ tế bào biểu mô, thâm nhiễm bạch cầu mức độ rất nhẹ, mạch máu sung huyết rất nhẹ, mô đệm phù nề rất nhẹ	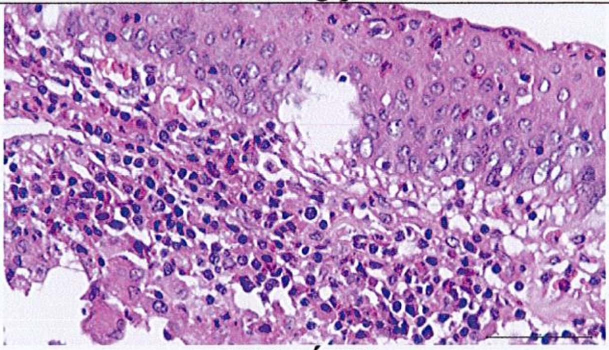<br>**Thỏ số 199**<br>Thoái hóa nhẹ tế bào biểu mô, thâm nhiễm bạch cầu nhẹ, mạch máu sung huyết và mô đệm không phù nề
3	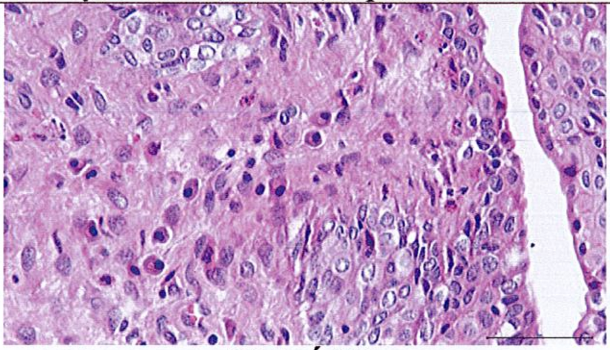<br>**Thỏ số 201**<br>Thoái hóa nhẹ tế bào biểu mô, thâm nhiễm bạch cầu rất nhẹ, mạch máu không sung huyết, mô đệm không phù nề	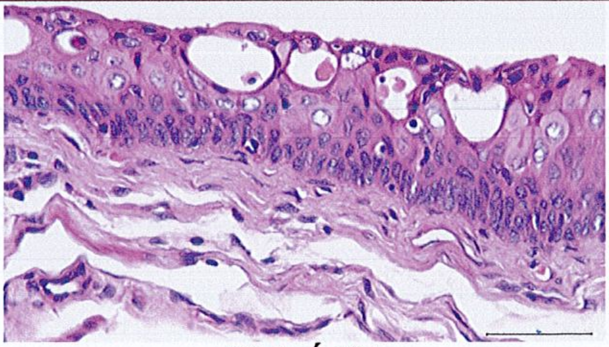<br>**Thỏ số 181**<br>Thoái hóa nhẹ tế bào biểu mô, thâm nhiễm bạch cầu rất nhẹ, mạch máu không sung huyết, mô đệm không phù nề

3.4. Phân loại

Căn cứ vào hướng dẫn phân loại mức độ kích ứng niêm mạc âm đạo của ISO 10993-23: 2021, với kết quả thu được trên thực nghiệm, mẫu thử được phân loại vào mức độ không gây kích ứng âm đạo.

C. KẾT LUẬN

Mẫu thử LiveSpo® X-SECRET gửi tới yêu cầu thử kích ứng âm đạo trên thỏ theo ISO 10993-23:2021 có kết quả như sau:

Mẫu thử được tiếp xúc với niêm mạc âm đạo thỏ với mức liều 1,0 mẫu thử/thỏ/ngày (tương đương ≥ 1 x 10^9 CFU *Bacillus subtilis, Bacillus clausii* và *Bacillus coagulans*/thỏ/ngày), dùng liên tiếp trong 5 ngày, mỗi ngày tiếp xúc mẫu thử 1 lần vào một khoảng giờ cố định. Kết quả thực nghiệm cho thấy mẫu thử không gây kích ứng âm đạo.

**Xác nhận của cơ quan**	*Hà Nội, ngày 22 tháng 10 năm 2024*
**Viện trưởng**	**Khoa Dược lý**
BỘ Y TẾ VIỆN KIỂM NGHIỆM THUỐC TRUNG ƯƠNG	
**PGS.TS. Đoàn Cao Sơn**	**ThS. Trần Thị Thanh Huế**
